# Skin-inspired phototherapeutic cryogel ameliorates infected wound healing by orchestrating mechanotransduction and immunomodulation

**DOI:** 10.1016/j.bioactmat.2025.11.028

**Published:** 2025-11-29

**Authors:** Sayan Deb Dutta, Jeong Man An, Md Moniruzzaman, Rumi Acharya, Youjin Seol, Hojin Kim, Aayushi Randhawa, Jong-Sung Kim, Yong-kyu Lee, Ki-Taek Lim

**Affiliations:** a Department of Biosystems Engineering, Kangwon National University, Chuncheon-si, 24341, Republic of Korea; b Institute of Forest Science, Kangwon National University, Chuncheon-si, 24341, Republic of Korea; c School of Medicine, The University of California Davis, Sacramento, 95817, United States; d Department of Bioengineering, Hanyang University, Seoul, 04763, Republic of Korea; e Department of Chemical and Biological Engineering, Gachon University, Seongnam-si, 13120, Republic of Korea; f Interdisciplinary Program in Smart Agriculture, Kangwon National University, Chuncheon-si, 24341, Republic of Korea; g Department of Chemical and Biological Engineering, Korea National University of Transportation, Chungju-si, 27470, Republic of Korea

**Keywords:** Chronic wound healing, Oxidative stress, Metal-organic framework, DNA cryogel, Photobiomodulation

## Abstract

Skin rehabilitation in clinics is seriously threatened by chronic wounds developed from drug-resistant bacteria, such as methicillin-resistant *Staphylococcus aureus* (*MRSA*), which frequently exhibit delayed healing owing to the hyperactive pro-inflammatory response, oxidative stress amplification, and fibrosis induction. Inspired by the skin epidermis, herein, we developed a deoxyribonucleic acid (DNA)/gelatin methacrylate (GelMA)-based soft cryogel platform that incorporates metal-organic framework (MOF) decorated plasmonic Ti_3_C_2_T_x_ (MXene) as a photosensitizer to prevent *MRSA* infection and promote scarless wound healing. By leveraging the bioactive and near-infrared (NIR) responsive property of the cryogel, mild phototherapy resulted in robust bactericidal performance and functional tissue regeneration while attenuating oxidative stress and maintaining hydrophilicity. Additionally, the transcriptome study verified that photobiomodulation by cryogel increased signature biomarkers that activate cytokeratin and zinc finger proteins owing to the Zn^2+^ ion adsorption to keratinocytes, highlighting its significant role in wound remodeling. Consequently, *in vivo* studies further disclose that cryogel-induced photobiomodulation promotes angiogenesis, thick epidermis generation, superior granulation, hair follicle growth, and anti-inflammatory activation without scarring, comparable to native skin. These findings underscore the remarkable potential of the fabricated cryogel as an innovative wound dressing material, providing an irresistible solution for managing infected skin wounds.

## Highlights

• DNA-based cryogel with MXene combats MRSA and supports chronic wound healing.
• NIR-triggered mild phototherapy ensures strong antibacterial and healing effects.
• Cryogel reduces oxidative stress and retains skin hydration for better recovery.
• Zn^2+^ ions enhance keratinocyte activity for improved wound remodeling.
• In vivo results show scarless healing with hair growth and tissue regeneration.

## Graphical abstract

**Figure:**
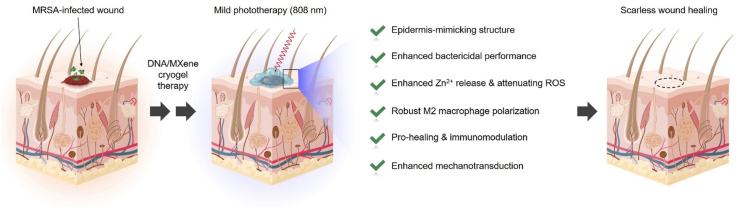

## 1 Introduction

Chronic wounds (i.e., non-healing wounds) present a potential threat to humanity and impose a significant socio-economic burden due to the rapid emergence of drug-resistant microorganisms (DRMs) [[1], [2], [3]] The annual economic cost of all chronic non-healing wounds in the United States alone is estimated to exceed $50 billion [1] The four primary phases of healing that normal wounds undergo are coagulation, inflammation, proliferation, and maturation. Additionally, chronic wound healing has an extended inflammatory phase caused by the accumulation of DRMs in the wound bed and the formation of biofilms, resulting in a stall in healing (typically lasting from five weeks to several months) and often leading to fibrotic scar formation [4] According to a meta-analysis, most chronic wounds harbor approximately 70 % biofilms even following appropriate treatment. Specifically, methicillin-resistant *Staphylococcus aureus* (*MRSA*) infections are prevalent in clinics treating chronic skin wounds and rank among the most infectious human pathogens identified since 1961 [5]. Furthermore, MRSA-infected wounds are known to secrete high levels of inflammatory cytokines. At the same time, the bacteria release metabolites such as lactic acid, malic acid, and acetic acid, resulting in a wound pH of 4–6. Consequently, most commercial wound dressings containing antibacterial agents fail to eradicate *MRSA* due to their inactivation at this much lower pH [2,6]. Recent studies on chronic wounds indicate that pro-inflammatory and anti-inflammatory signals compete, leading to a redox imbalance that disrupts routine wound healing [7,8]. This imbalance hinders wound closure by maintaining it in a persistent inflammatory state. Reactive oxygen species (ROS) are released by invading neutrophils and macrophages to combat microbial colonization during ongoing inflammation. Chronic wounds with elevated ROS levels have a detrimental effect on healing, as they can damage cells, tissues, and the extracellular matrix (ECM), as well as activate inflammatory cytokines and latent extracellular proteases like matrix metalloproteinases (MMPs) [1,8].

To address these issues, advanced combat strategies for MRSA-infected chronic wounds require new multidisciplinary and stimuli-responsive dressings that prevent bacterial infection and accelerate the healing process without scarring. Such a multifunctional bioactive platform acts as a barrier against pathogen bacteria and promotes thick epidermis formation to minimize moisture loss and electrolyte imbalance and maintain tissue homeostasis [8] Recently, several efforts have focused on fabricating skin ECM-mimicking and antibiotic hydrogel-based platforms for delivering therapeutic agents, such as peptides [2,9] proteins [10] genes [11] drugs [12] metal ions [13] and exosomes [14] to halt the progression of chronic wounds. As previously discussed, conventional drugs often fail to stop DRM invasion due to their low stability, bioactivity, tough, tear resistant, and delivery efficiency from hydrogels, creating a significant bottleneck for clinical application By integrating double-network structures, nanocomposites, and dynamic reversible bonds, hydrogels achieve mechanical robustness and autonomous repair, extending their durability for wound healing and mimic the native skin microenvironment. Their high water content and biocompatibility provide a moist environment that promotes wound healing, drug delivery, and tissue regeneration [15]. For example, researchers are using various micro/nanoparticles incorporated into hydrogels, which can exhibit higher antimicrobial performance under specific stimuli such as light [16] temperature [17] electric fields [18] pH [12,19] glucose [19] and others. In this context, near-infrared (NIR)-responsive two-dimensional (2D) phototherapeutic agents (PAs) could serve as an ideal alternative to conventional antimicrobial agents, due to their higher photothermal conversion efficiency (PCE) and hyperthermia-induced delivery of therapeutic agents for chronic wound healing [16,20] Increasing evidence indicates that Ti_3_C_2_T_x_ (MXene) and MXene-based 2D carbides demonstrate superior PCE because of their semiconductor nature, higher surface plasmonic resonance (SPR), ability to generate electron-holes, and enhanced electron-photon coupling effect (LUMO → HOMO) under near-infrared (NIR) light stimulation [21,22] Additionally, hetero-atom doping on the MXene primarily enhances the PCE through conjugation or hyperconjugation effects, resulting in a rapid temperature increase [22] Previously, MXene-integrated hydrogels exhibited excellent antibacterial performance and wound healing properties; however, most hydrogels do not possess skin ECM-mimicking properties, leading to a slow healing rate [23,24] In particular, while most MXene-based hydrogels have shown remarkable *in vivo* wound healing, the long-term regenerative performance, skin re-epithelization, inflammatory responses, scarring potential, and underlying mechanisms are not yet well described.

**Scheme 1:**
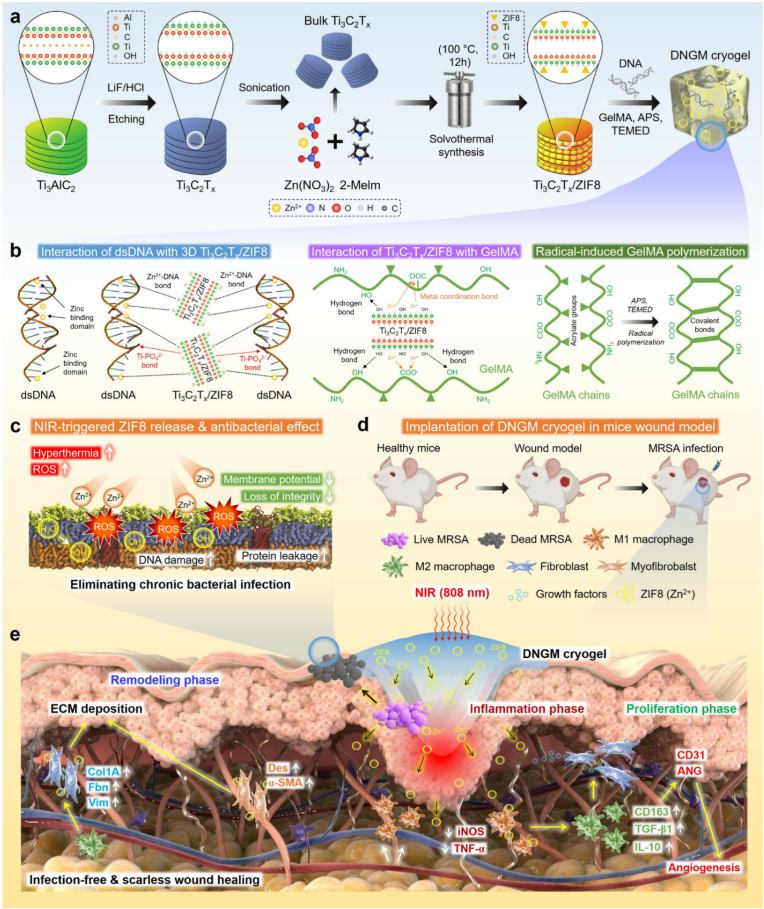
Schematic illustration of the phototherapeutic DNA cryogel fabrication process incorporating MXene@ZIF8 for chronic wound healing. **(a, b)** Fabrication process and crosslinking mechanisms of the cryogel and **(c, d)** its skin regenerative potential under photobiomodulation.

Inspired by the skin epidermis, we report a novel phototherapeutic deoxyribonucleic acid/gelatin methacrylate/Ti_3_C_2_T_x_/zeolite imidazolium framework-8 (=DNGM) cryogel platform, mimicking the structural components of native extracellular matrix (ECM) to combat *MRSA* biofilms and facilitate guided wound healing (Scheme 1). The designed soft and elastic cryogel possesses ‘*all-in-all*’ potential compared to other DNA-based hydrogels reported thus far. Utilizing the bioactive DN [15,25] and GelMA [26], the Ti_3_C_2_T_x_/zeolite imidazolium framework-8 (=MXene@ZIF8) is introduced to enhance the mechanical and viscoelastic properties through dynamic bond formation (*e*.*g*., DNA/Zn^2+^, hydrogen bonds, metal coordination, and covalent bonds). The biomimetic porous scaffold network provides a protective and photothermal interface that mimics the barrier function of native skin, effectively restricting bacterial infiltration while enabling NIR-induced heat dissipation. Meanwhile, the anisotropic skin-like porosity (lamellar pores obtained through directional freezing at low temperature) supports keratinocytes infiltration, Zn^2+^-mediated angiogenesis, and immunomodulation. The gradient in crosslinking density and pore size thus couples the physical mimicry of skin architecture with coordinated antibacterial and regenerative responses. Furthermore, the *in vivo* results show that the DNGM cryogel, when combined with mild phototherapy (1.0 W cm^−2^, 808 nm), can effectively eliminate persistent *MRSA* infections and support wound healing by promoting thick epidermis, granulation tissue, angiogenesis, hair follicle formation, and anti-inflammatory activity, underscoring its potential for restoring skin function.

## 2 Results and discussion

### 2.1 Characterization of the nanocomposite cryogel

**Fig. 1:**
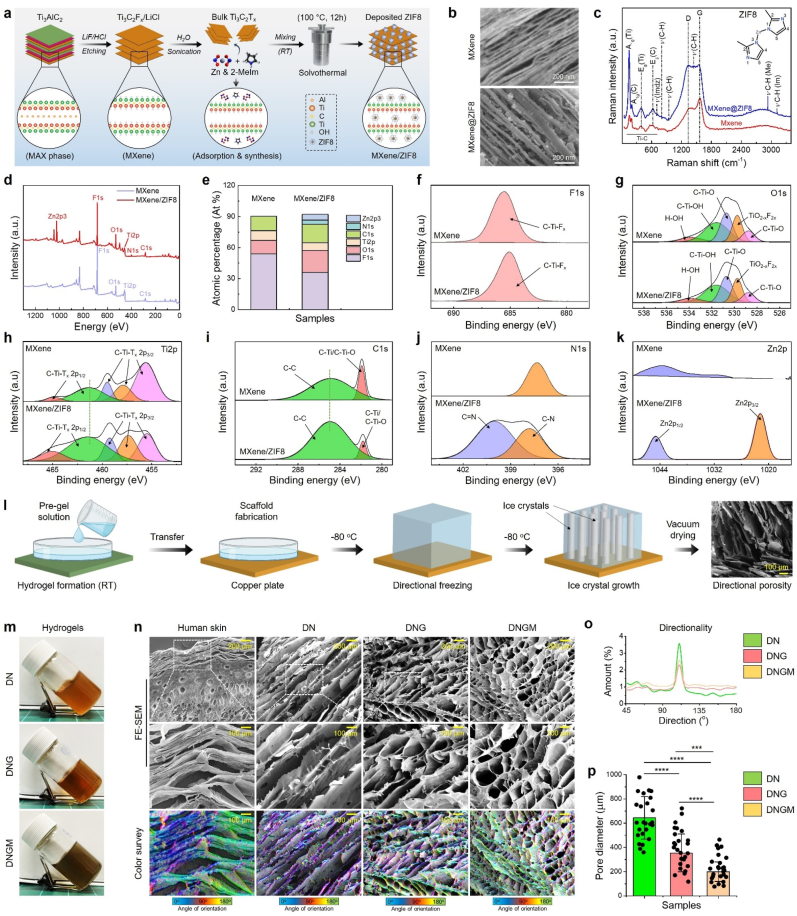
Characterization of the nanocomposite cryogel scaffold. **(a)** Schematic illustration of the hydrothermal synthesis of MXene@ZIF8. **(b)** FE-SEM images of the pristine MXene and MXene@ZIF8 nanocomposites. **(c)** FT-IR spectra of the MXene and MXene@ZIF8. **(d)** XPS survey spectra of the MXene and MXene@ZIF8 nanocomposites. **(e**–**k)** High-resolution XPS spectra of the MXene and MXene@ZIF8. **(l)** Schematic illustration of the cryogel fabrication process inspired by skin epidermis. **(m, n)** Representative digital photographs and FE-SEM micromorphologies with corresponding color survey maps of the cryogels. A cryo-SEM image of a human skin sample was taken as a comparison. Scale bar: 200 μm. **(o)** Pore directionality map of the fabricated cryogels. **(p)** Calculation of pore diameters of the fabricated cryogel scaffolds (*n* = 25 each). Statistical significance was considered at ∗∗∗*p* < 0.001 and ∗∗∗∗*p* < 0.0001 (One-way ANOVA followed by Tukey's HSD *post-hoc* test).

The schematic illustration of the MXene@ZIF8 fabrication process is shown in Fig. 1(a). The MXene (=Ti_3_C_2_T_x_) was synthesized from the MAX phase of Ti_3_AlC_2_, while the ZIF8 was intercalated onto the MXene nanosheets using a solvothermal method. Fig. S1 illustrates the digital photographs of the macroscopic appearance of the pristine MXene and MXene@ZIF8 nanocomposites. The morphology of the MXene and MXene@ZIF8 was investigated by scanning electron microscope (SEM), and the result is shown in Fig. 1(b). The results show that bulk MXene displayed a unique layered (=packed accordion-like) structure after etching and sonication [27,28], while small particles (oval-to-rod shaped) were observed onto the layers in MXene layer interfaces, indicating the presence of ZIF8 metal-organic framework (MOF) deposition [29,30]. Moreover, the X-ray diffraction (XRD) analysis (Fig. S2) indicates that both MXene and MXene@ZIF8 exhibited notable peaks at around 2θ = 43.37° and 58.72° correspond to the Al [104]. Furthermore, several diffraction peaks at 2θ = 18.46° [004], 25.09° [006], 35.21° [103], and 44.93° [107] were assigned to the Ti_3_C_2_ structure, resembling the Ti-C framework of MXenes [28,31]. Importantly, several other diffraction peaks at 2θ = 26.11°, 29.76°, 37.88°, 48.13°, 53.72°, 60.69°, 65.46°, and 68.39° were found in MXenes, corresponding to the graphitic-C [32] and oxide peaks of TiO_2_ [33]. Notably, in MXene@ZIF8, characteristic diffraction peaks at 2θ = 19.70° [223], 21.51° [114], 30.16° [044], and 38.64° were found, which matches with the JCPDS: 00-062-1031, confirming the presence of deposited ZIF8 particles [34]. We investigated Raman spectroscopy to gain insights into the structural changes of the bulk MXene and MXene@ZIF8. As illustrated in Fig. 1(c), bulk MXene exhibited signature peaks at around 155.35 and 390.04 cm^−1^, resembling resonance and in-plane vibrations of Ti-C (E_g_) and Ti-O (E_g_). Moreover, peaks at 208.49 and 627.35 cm^−1^ were assigned to the out-of-plane Ti-C (A_1g_) bond [30]. A small peak at around 409 cm^−1^ in MXene was assigned to the oxygen (O) bound to the Ti-C (Ti_3_C_2_(OH)_2_) framework [35]. Notably, the MXene@ZIF8 nanocomposite displayed additional peaks at around 707.56, 943.07, and 1447.89 cm^−1,^ owing to the blending vibrations of out-of-plane imidazolium (*v*_Imdz_) and methyl (C-H) bending (C4-C5) vibrations of the ZIF8, respectively [36]. Besides, two peaks at 2931.12 and 3113.34 cm^−1^ were attributed to the anti-asymmetric stretching of C-H, C-H (aromatic), and C-H blending vibrations of imidazolium (*v*_Imdz_) ring, suggesting the incorporation of the ZIF8 particles. Furthermore, we observed two signature peaks for both MXene and MXene@ZIF8 at around 1372 and 1580 cm^−1,^ indicating the D-band (A_1g_) and G-band (E_2g_) of the out-of-plane vibrations of the carbon atom [36,37], suggesting that the native structure of Ti_3_C_2_ was preserved after Al removal.

The chemical composition and electronic properties of the MXene and MXene@ZIF8 were explored using X-ray photoelectron spectroscopy (XPS), and the result is shown in Fig. 1(d). The XPS survey spectra of bulk MXene revealed the presence of C1s, N1s, O1s, Ti2p, and F1s. By contrast, the MXene@ZIF8 nanocomposite additionally showed the presence of Zn2p spectra. The element composition is shown in Fig. 1(e). The percentage of Zinc composition was calculated to be 5.68 ± 1.76 At. % for MXene@ZIF8. Besides, the element composition of carbon, oxygen, and nitrogen was found higher for MXene@ZIF8 (C1s: 17.99 At. %, O1s: 21.23 At. %, and N1s: 3.99 At. %) than pure MXene (C1s: 14.15 At. % and O1s: 13.19 At. %), suggesting the successful incorporation of ZIF8 onto the MXene surface. The high-resolution F1s spectra of MXene and MXene@ZIF8 (Fig. 1(f)) show binding energies at 685.49 and 685.06 eV, indicating the presence of the C-Ti-F_x_ bond [38]. Subsequently, the high-resolution O1s spectra of both MXene and MXene@ZIF8 (Fig. 1(g)) contain peaks at 528.73, 529.61, 530.48, 531.56, and 533.82.84 eV, which can be assigned to the C-Ti-O, TiO_2-x_F_x_, C-Ti-O, C-Ti-OH, and H-OH bonds [38,39]. Surprisingly, the Ti2p spectra showed a notable change after ZIF8 incorporation in MXenes. As depicted in Fig. 1(h), both MXene and MXene@ZIF8 exhibited three peaks at 455.56, 457.86, and 459.47 eV for C-Ti-T_x_ 2p_3/2,_ while two other peaks at around 461.35 and 464.84 eV for C-Ti-T_x_ 2p_1/2_, respectively. The integral area of the Ti 2p_1/2_ was found to be increased (2p_1/2_: AUC_MXene@ZIF8_ > AUC_MXene_) in MXene@ZIF8 than bulk MXene, while a slight decrease at Ti 2p_3/2_ was noticed, indicating the interaction of imidazolium rings and/or methyl (C-H) groups of ZIF8 with C-Ti framework [29,40]. Similarly, the high-resolution C1s spectra (Fig. 1(i)) of the MXene@ZIF8 exhibited significant change at a binding energy of 284.87 eV (C-C bond), suggesting the incorporation of ZIF8 onto the MXene structure. Furthermore, the high-resolution N1s and Zn2p spectra exhibited distinct peaks at around 397.79, 400.08, 1021.81, and 1044.92 eV, corresponding to the C-N, C=N, Zn2p_3/2_, and Zn2p_1/2_ (Fig. 1(j and k)), which was not found in bulk MXenes [29,30,38]. These findings underscore the successful fabrication of MXene@ZIF8 nanocomposites, which are mostly composed of elemental Zn, C-Ti-F_x_, C-Ti-O, H-OH, C-N, and C=N groups, respectively.

Owing to the attractive 2D structure, electronic configuration, tailorable surface functionalities, and higher photothermal properties, MXene and its hetero-nanocomposite-based hydrogels have gained enormous attention in tissue engineering, especially in diagnostics and wound healing [23,41]. In this study, we fabricated a DNA-based mild phototherapeutic cryogel platform to mimic the skin epidermis, the outer layer of skin with an anisotropic structure [42], and to combat drug-resistant pathogens toward chronic wound healing. The cryogel was designed using a DNA/GelMA-based soft ECM matrix, where MXene@ZIF8 helps in cross-binding DNA and/or GelMA via metal coordination and hydrogen bonds improving the overall mechanical and photothermal properties. The sustained release of Zn^2+^ from the MXene@ZIF8 through the cryogel in response to photothermal effect would enhance the skin regeneration potential via activating the matrix metalloproteinases (MMPs), attenuating reactive oxygen species (ROS) burden, keratinocytes migration, reducing inflammation, angiogenesis, and bacterial infection, resulting in scarless wound healing [43]. A schematic illustration of the cryogel fabrication process is shown in Figure S3 and Fig. 1(l). In a typical process, the aqueous solution of MXene@ZIF8 was introduced into a mixture of 6 % salmon sperm DNA (ssDNA, *w*/*v*) and 0.5 % GelMA (*w*/*v*), followed by radical polymerization using ammonium persulfate (APS) and tetramethylethylenediamine (TEMED). After that, the resulting solution was allowed to form a gel. Subsequently, resulting hydrogels (DN, DNG, and DNGM) were quickly transferred into a silicone mold and placed on the top of a copper plate (pre-cooled at −80 °C) to ensure unidirectional ice crystal growth. The digital photographs of the fabricated cryogels are shown in Fig. 1(m). The FE-SEM images with corresponding color survey maps of the native skin and freeze-dried cryogels are depicted in Fig. 1(n). Nevertheless, low GelMA concentration would not provide higher mechanical strength, but it could contribute to aligned cryogel porosity generation. The results show that compared to the native skin [44], our cryogel scaffolds displayed unique lamellar and mostly unidirectional pores in all the cryogels with a smaller pore size when we moved from DN (pure DNA gel) to DNGM (MXene@ZIF8 containing gel) and higher orientation degree (Fig. 1(o)), indicating the influence of GelMA and incorporated MXene@ZIF8 in regulating porosity, which is not commonly observed in conventional DNA-based hydrogels [11,15,45,46]. The DN and DNG cryogel scaffolds displayed a pore diameter of around 642 ± 56 μm (porosity: 77.4 ± 4.56 %) and 350 ± 50 μm (porosity: 61.5 ± 3.43 %), while the DNGM cryogel scaffold showed a pore diameter of 198 ± 40 μm (porosity: 49.8 ± 3.76 %), respectively (Fig. 1(p)).

The interaction between the DNA, MXene@ZIF8, and GelMA was studied using Fourier transform infrared (FT-IR) spectroscopy, and the result is shown in Fig. S4. The DN scaffold (=pure DNA) exhibited a peak around 3288 cm^−1^ owing to the presence of hydroxyl (–OH) moieties. The peaks at around 3285 and 3299 cm^−1^ in DNG and DNGM were attributed to the –OH and –NH groups of GelMA, with a shoulder peak at around 3052-3056 cm^−1^ due to the presence of methyl (–CH) groups [47]. In pure DN scaffold, a band at around 1590 cm^−1^ was assigned to the stretching vibration of the deoxyribose unit (C–C), while the bands at 1230 and 1053 cm^−1^ were attributed to the stretching vibrations for C–O–C/PO^2−^ and C–O/-S=P groups [48], respectively. Similarly, the DNG and DNGM scaffolds displayed three predominant peaks between 1650 and 1100 cm^−1^, indicating the presence of GelMA's Amide-I, Amide-II, and Amide-III regions [47]. Interestingly, after the incorporation of DNA and MXene@ZIF8, the GelMA's Amide-I band (1642 cm^−1^ → 1239 cm^−1^) was slightly shifted to the lower wavenumber, suggesting the interaction of DNA and/or MXene@ZIF8 with the GelMA matrix. Furthermore, the peak intensity at 1230 cm^−1^ in DN was slightly decreased and blended with Amide-III of both DNG and DNGM, indicating the interaction of DNA with MXene@ZIF8 and GelMA, respectively. The viscoelastic property of the developed cryogels was assessed using a rotational rheometer under varying frequencies (0.1–100 Rad s^−1^) at 25 °C. As depicted in Fig. S5(a), all the cryogels displayed a frequency-dependent change in storage (G′) and loss modulus (G″), with a greater increase in G′ value for DNGM than other groups (G′_DNGM_ > G′_DNG_ > G′_DN_), suggesting that MXene@ZIF8 incorporation significantly enhanced the elasticity of the DNA/GelMA matrix. The G′ values for DN, DNG, and DNGM at 100 Rad s^−1^ were calculated to be 0.271 kPa, 3.471 kPa, and 10.154 kPa, respectively. In addition, the cryogels also displayed a frequency-dependent change in complex viscosity (*η*∗) with high viscosity at a low-frequency range (0.1 Rad s^−1^) and low viscosity at a high-frequency range (100 Rad s^−1^) (Fig. S5(b)), suggesting its elastic-to-viscous transition, ideal for tissue engineering applications [49]. The *η*∗ values for DN, DNG, and DNGM at 100 Rad s-1 were calculated to be 695.15, 11335, and 24120 mPa s, respectively. The initial shear stress values for all the cryogel are given in Fig. S5(c).

The mechanical properties of the DNA-based hydrogels can be tailored by changing the nucleotide length, crosslinking strategy, and incorporating other biopolymers [50]. In this context, we then evaluated the mechanical properties of the developed cryogels by compressive test. Fig. S6(a) represents the compressive stress-strain curve of the DN, DNG, and DNGM cryogels. It was worth noticing that DNGM cryogel displayed significantly higher compressive strength (∼16.9 ± 0.45 kPa, ∗*p* < 0.05) and elastic modulus (∼1.14 ± 0.61 kPa, ∗∗∗∗*p* < 0.0001) than other samples (Fig. S6(b and c)), suggesting that both DNA and MXene@ZIF8 incorporation and its crosslinking with GelMA resulted in soft and viscoelastic cryogel, which was surprisingly higher than previously reported DNA hydrogels. A comparative study on the mechanical and viscoelastic properties of various DNA-based hydrogels is listed in Table S1. The swelling property of the developed cryogel scaffolds was investigated in 1 × PBS up to 24 h, and the result is shown in Fig. S7(a). Notably, we found a slight decrease in swelling efficiency when we moved from the DN → DNGM scaffold, which can be explained by the decrease in pore size DNGM than DN (642 μm → 198 μm). Nevertheless, all the cryogel samples displayed a swelling degree of ≥ 100 % after 24 h, suggesting that it could be ideal for biological studies. It was also reflected in the degradation behavior of the cryogel scaffolds. As depicted in Fig. S7(b), the DN (∼50 %) scaffold with greater pore size exhibited a higher degradation rate than DNG (∼43 %) and DNGM (∼25 %) scaffolds at day 7. Moreover, the DNGM cryogel degradation is also assessed in cell culture media (=DMEM). This process is necessary for the final removal of the applied hydrogel and restricts the amount of time that hydrogel-mediated biological reactions can last [51]. As shown in Fig. S7(c), the reducing sodium dodecyl sulfate-polyacrylamide gel electrophoresis (SDS-PAGE) showed controlled degradation of DNGM in DMEM media, which was characterized by the increasing band size and intensity when we moved from day 0 to day 7, suggesting its good biodegradability in biological fluids. Besides, enzymatic degradation with pronase and trypsin (Fig. S7(d and e)) further validated a slight faster but controlled degradation profile of DNGM up to day 7, underscoring that the DNA double-helix network and MXene@ZIF-8 coordination enhance proteolytic resistance and thereby improving structural integrity, which would be beneficial for infected wounds. Using both pronase and trypsin provides a comprehensive evaluation of the cryogel stability, since pronase mimics broad non-specific proteolysis while trypsin reflects selective enzymatic cleavage, together simulating different physiological degradation environments. Taken together, we anticipate that the DNGM cryogel is best in terms of mechanical and viscoelastic properties and exerts good biodegradation, which could be an ideal soft hydrogel for skin tissue engineering.

**Fig. 2:**
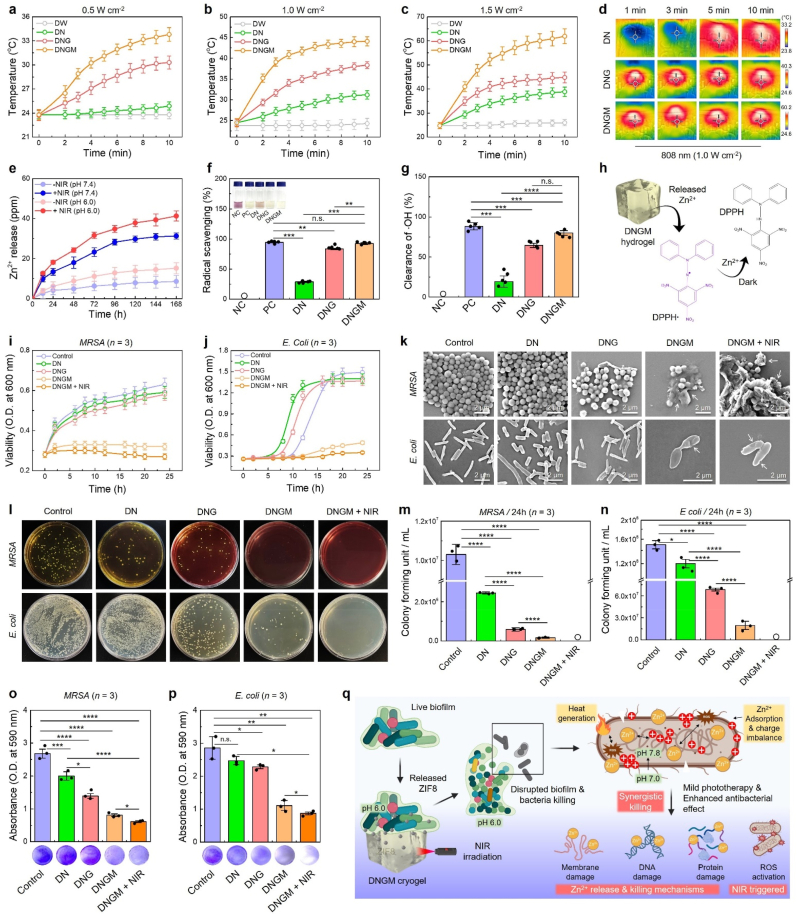
*In vitro* bioactivity of the fabricated cryogel scaffolds. **(a**–**c)** Time-dependent temperature rise profiles of the fabricated cryogel scaffolds under various laser (808 nm) power densities (0.5, 1, and 1.5 W cm^−2^) **(d)** Representative NIR thermal images showing the temperature profile of the cryogels at 1 W cm^−2^. **(e)** Zn^2+^ release profile from DNGM cryogel w/or w/o NIR irradiation (1.0 W cm^−2^) at pH 6.0 and 7.4 up to 7 days. **(f, g)** DPPH and · OH radical scavenging potential of the fabricated cryogels (*n* = 5 each). **(h)** Mechanism of DPPH radical scavenging property of DNGM cryogel. **(i, j)** Representative growth curve of the *MRSA* (gram-positive) and *E. coli* (gram-negative) at indicated time points. **(k)** FE-SEM images of the surface morphology of the *MRSA* and *E. coli* in various treatment groups after 24 h of incubation. Scale bar: 2 μm. **(l)** Digital photographs of the agar plates showing the antibacterial efficacy of the fabricated cryogels with **(m, n)** corresponding quantification data (*n* = 3 each). **(o, p)** Digital photographs with corresponding statistical data of the antibiofilm assay with various formulations (*n* = 3 each). **(q)** Schematic illustration showing the mechanisms of the antibacterial performance of the fabricated cryogel scaffold. Data reported as mean ± s.d. of replicated experiments, statistical significance was considered at ∗*p* < 0.05, ∗∗*p* < 0.01, ∗∗∗*p* < 0.001, and ∗∗∗∗*p* < 0.0001 (One-way ANOVA followed by Tukey's HSD *post-hoc* test).

To investigate the photothermal properties of the DNGM cryogel, we performed the macroscopic heat generation properties, which would effectively kill the DRMs. In this context, ZIF8-decorated MXene would exert better PCE. Before that, we compared the photothermal properties of pristine MXene and MXene@ZIF8. We used 100 μg mL^−1^ concentration of MXene and MXene@ZIF8 for this study, owing to their mild temperature (∼45–55 °C) rise [22], which would favor Zn^2+^ release and produce sufficient hyperthermia for killing bacteria-infected wounds. The ultraviolet–visible (UV–Vis) spectra of the MXene and MXene@ZIF8 exhibited an absorption peak at around 823 nm within a scan range of 450–950 nm (Fig. S8), indicating its NIR responsiveness. Inspired by this finding, we used 100 μg mL^−1^ of MXene@ZIF8 concentration for DNGM cryogel preparation and subsequent NIR irradiation with varying laser power densities (0.5, 1.0, and 1.5 W cm^−2^). As shown in Fig. 2(a–c) and Fig. S9(a), the DNGM cryogel exhibited a laser power-dependent temperature rise profile within a time frame of 0–10 min, respectively. Besides, the DN and DNG cryogels did not show any obvious change in temperature rise. It has been reported that NIR light (*e*.*g*., 808–860 nm) with a power density ranging from 0.01 to 1.0 W cm^−2^, generating a temperature of around 40–45 °C, is sufficient for drug delivery, antimicrobial sterilization, anti-inflammation, and wound healing in clinical applications [52]. Thus, we selected 1.0 W cm^−2^ laser power and 10 min irradiation time for later experiments unless stated elsewhere. The NIR thermal images of DN, DNG, and DNGM are shown in Fig. 2(d).

The origin of the photothermal property of DNGM cryogel can be explained by PCE calculation. A detailed study on PCE revealed that MXene@ZIF8 (∼35.57 %) exerts higher PCE than pristine MXene (∼30.98 %) (Fig. S9(b–e)), which is probably due to the doping of ZIF8, a good photothermal agent [29]. The NIR thermal images of the photothermal experiment are shown in Fig. S9(f). To gain insights into the origin of PCE, we further studied the energy levels of the frontier orbital (HOMO-LUMO and band gaps) for both MXene and MXene@ZIF8 using cyclic voltammetry. As depicted in Fig. S9(g and h), the pristine MXene shows *E*_HOMO_ and *E*_LUMO_ of around 4.98 eV and 4.01 eV. Additionally, the MXene@ZIF8 exhibits EHOMO and ELUMO values of approximately 5.16 eV and 4.62 eV, respectively. Interestingly, the calculated band gap (*E*_g_) value was found much lower in MXene@ZIF8 (*E*_g_ = 0.54 eV) than pristine MXene (*E*_g_ = 0.97 eV), suggesting its strong NIR absorption and efficient heat conversion efficiency (Fig. S9(i)). Moreover, the DNGM cryogel exhibited excellent cyclic thermal stability under repeated NIR irradiation, as the temperature consistently increased to ∼41–43 °C during the “ON” cycles and returned near baseline (24-24 °C) during the “OFF” periods (Fig. S10). This reversible heating and cooling behavior over three cycles demonstrates its reliable photothermal responsiveness and stability. Thus, DNGM cryogel with a higher PCE could be beneficial for photobiomodulation and NIR-triggered therapeutic studies, especially for drug delivery and antibacterial studies.

To investigate whether mild phototherapy has any effect on Zn^2+^ release and subsequent antioxidant properties, we next performed the Zn^2+^ release study from the DNGM cryogel scaffold w/or w/o NIR stimulation (1.0 W cm^−2^, 10 min, and 808 nm) at pH 6.0 and 7.4 up to 7 days *in vitro*. As depicted in Fig. 2(e), at acidic pH (6.0), Zn^2+^ release is significantly higher compared to neutral pH (7.4), and NIR exposure further enhances this release. The highest Zn^2+^ release is observed under combined conditions of pH 6.0 with NIR, reaching ∼40 ppm after 168 h. This suggests that both acidic environment and NIR irradiation synergistically accelerate Zn^2+^ release. Therefore, we hypothesized that NIR-triggered Zn^2+^ release from DNGM would favor skin wound healing in two ways: (1) by killing pathogenic bacteria in the wound bed by Zn^2+^ uptake at acidic pH and hyperthermia [53] and (2) by promoting skin cell migration. To examine the antioxidant-like nature of the fabricated cryogels, we studied the 2,2-diphenyl-1-picrylhydrazyl (DPPH) assay to determine hydroxyl (· OH) radical scavenging potential. As shown in Fig. 2(f and g), the DNG and DNGM exhibited significantly higher (∗∗*p* < 0.01 and ∗∗∗*p* < 0.001) rates of DPPH and · OH radical scavenging potential than DN or DNG, which was comparable with positive control (=ascorbic acid, 0.5 mM). The greater radical scavenging property of DNGM was probably due to the released Zn^2+^ from MXene@ZIF8, which stabilized the DPPH radicals (Fig. 2(h)) [29,54]. Our findings underscore the excellent mechanical, viscoelastic, and antioxidant properties of the DNGM cryogel, which could be beneficial for treating infected wounds in clinical settings.

### 2.2 Mild phototherapy enhances the bactericidal performance of DNGM cryogel

Understanding the fact that Zn^2+^ ions effectively kill bacteria by producing ROS, disrupting bacterial enzyme systems, inhibiting protein and DNA synthesis, and interacting with the bacterial cell wall [53,55], we then investigate the bactericidal efficacy of the fabricated cryogels w/or w/o NIR stimulation against *Escherichia coli* (*E. coli*, gram-negative) and methicillin-resistant *Staphylococcus aureus* (*MRSA*, gram-positive) bacteria, respectively. The bactericidal property was investigated by growth curve inhibition (O.D.-based assay) test, plate-dilution test, morphological assessment, and biofilm inhibition assay. The control group received only saline. As illustrated in Fig. 2(i and j), DN and DNG exhibited less growth inhibition for *E. coli* and *MRSA* within 0–24 h. This would probably be due to the non-toxic nature of the DNA and GelMA, which facilitated the higher proliferation of *E. coli* and *MRSA*. Subsequently, the DNGM and DNGM + NIR group showed a significantly higher degree of growth inhibition for both *E. coli* (∗∗∗∗*p* < 0.0001) and *MRSA* (∗∗∗∗*p* < 0.0001) than the control, suggesting that the incorporation of MXene@ZIF8 effectively killed the bacteria. The growth inhibition for *MRSA* started from 1 to 2 h, while it was around 5–6 h for *E. coli*. It was also verified that in the presence of pure MXene and MXene@ZIF8, the *E. coli* viability was reduced to almost half (50 %) at 100 μg mL^−1^ concentration (Fig. S11), further confirming that the bactericidal efficacy was due to the MXene [56] and the released Zn^2+^ ions from ZIF8 upon NIR irradiation [57]. This outstanding growth inhibits the effect of DNGM cryogel w/NIR, which seems to depend on the excellent hyperthermia-induced ROS activation and charge imbalance in the cell membrane of both *E. coli* and *MRSA*.

The excellent growth inhibition property of the DNGM cryogel further motivated us to explore the detailed antibacterial mechanisms in *E. coli* and *MRSA*. For this, we examined the morphology of the bacteria by SEM, while the colony inhibition study was performed by plate dilution test after 24 h of culture. Fig. 2(k) shows that *MRSA* and *E. coli* exhibited smooth surfaces in the control and DN groups, suggesting healthy bacterial morphology. Interestingly, in the DNG group, some of the bacteria (∼5–10 %) in both *MRSA* and *E. coli* tend to exhibit slightly collapsed morphology, which would probably be due to the interaction between the bacterial cell membrane and the amine groups of the GelMA. More interestingly, in the DNGM and DNGM + NIR groups, a higher amount (>70 %) of bacteria showed punctured and collapsed morphology, characterized by visible damage in the cell membrane (=perforated membrane). Thus, we concluded that the MXene@ZIF8 containing DNGM cryogel with mild phototherapy significantly disrupted the membrane integrity of *E. coli* and *MRSA* by generating hyperthermia and ROS amplification. The agar colony formation assay also demonstrated a similar trend. As depicted in Fig. 2(l), both *MRSA* and *E. coli* showed a greater number of visible colonies in the control, DN, and DNG groups. The DNGM group displayed fewer colonies on agar plates, followed by no visible colony formation in the DNGM + NIR group, suggesting their killing efficacy. The statistical analysis of the colony formation assay suggested a significantly reduced colony forming unit (CFU) for both *MRSA* (∗∗∗∗*p* < 0.0001) and *E. coli* (∗∗∗∗*p* < 0.0001) cultured with DNGM w/or w/o NIR. It is worth noticing that the DNGM cryogel can exert superior antibacterial performance even after first use against *E. coli*, as confirmed by the agar colony formation and live/dead assay (Fig. S12(a–c)). The inhibition of colony formation after cyclic NIR stimulation was achieved through the released Zn^2+^ and heat generation from DNGM cryogel.

During chronic wound healing, bacterial mass tends to form thick extracellular polymeric substances (EPS), ensuring their survival, drug escape, and resistance. Thus, biofilm formation at or near the wounded skin often causes inflammation and secondary infection, leading to impaired healing and skin integrity [58]. To investigate the efficacy of the fabricated cryogels in biofilm eradication w/or w/o NIR, we tested the bacterial biofilm inhibition test, and the results are shown in Fig. 2(o and p). Not surprisingly, the control group (PBS-treated) of both *MRSA* and *E. coli* exerts no change in biofilm reduction as evidenced by higher O.D. value of crystal violet and macroscopic image. Meanwhile, the DN and DNG groups also displayed no significant reduction in biofilm formation, as evidenced by the dark crystal violet color formation. Interestingly, in DNGM and DNGM + NIR groups, a significant reduction in biofilm formation was documented for both *MRSA* (∗∗∗∗*p* < 0.0001) and *E. coli* (∗∗*p* < 0.01) than control and other groups, as characterized by the reduction of crystal violet stain and OD values. Membrane damage often leads to surface charge imbalance and protein leakage in bacteria, resulting in intracellular and extracellular redox homeostasis [19]. This was also reflected when repeated NIR stimulation was carried out with DNGM up to 72 h. As depicted in Fig. S12(d) and E *coli* and *MRSA* biofilm formation potential was significantly (∗∗∗∗*p* < 0.0001) reduced in both DNGM and DNGM + NIR groups, suggesting that the fabricated DNGM cryogel with mild phototherapy can effectively eliminate persistent bacterial infection from wounded tissue and reduce the pathogenic biofilm formation. The statistical analysis of biofilm disruption study up to 72 h is showing in Fig. S12(e). To find the MXene@ZIF8-assisted bacterial cell disruption, we next investigated the surface charge after the desired treatments. As depicted in Fig. S13(a and b), the control groups of *MRSA* and *E. coli* exhibit a zeta potential of −18.55 and −22.6 mV, respectively. Notably, in the DNGM and DNGM + NIR groups, the zeta potential was significantly increased for *MRSA* (−6.54 mV, ∗∗∗∗*p* < 0.0001) and *E. coli* (−9.83 mV, ∗∗∗∗*p* < 0.0001), suggesting a disruption or cationic charge adsorption (*e.g.*, Zn^2+^) onto its surfaces upon photothermal effect [59]. Similarly, compared to control, DN, and DNG groups, the protein leakage (Fig. S13(c)) was found significantly (∗∗∗*p* < 0.001) higher for both *MRSA* (∼200 μg mL^−1^) and *E. coli* (∼280 μg mL^−1^) in DNGM and DNGM + NIR groups, collectively referring the cell lysis and redox imbalance. Moreover, the inductively coupled plasma mass spectrometry (ICP-OES) analysis after DNGM and DNGM + NIR treatment revealed traces of Zn^2+^ in *MRSA* (8.84 ppm) and *E. coli* (11.2 ppm) after 24 h (Fig. S13(d)), further conferring the source of charge imbalance, ROS activation, biofilm inhibition, and apoptotic cell death [60], which confirmed the FE-SEM data. These findings underscore the remarkable antibiofilm efficacy of the DNGM cryogel, and mild phototherapy synergistically improved its efficacy. A schematic illustration of the DNGM-induced antibacterial mechanism is shown in Fig. 2(q). Taken together, our results showed excellent bactericidal efficacy of DNGM cryogels with mild phototherapy, where the released Zn^2+^ from the MXene@ZIF8 at acidic pH (pH 6.0) participated in membrane disruption, ROS activation, adsorption, lipid peroxidation, pH imbalance, and hindered metabolism, collectively leading to bacterial cell death [61].

### 2.3 DNGM primes keratinization and wound healing under mild phototherapy

**Fig. 3:**
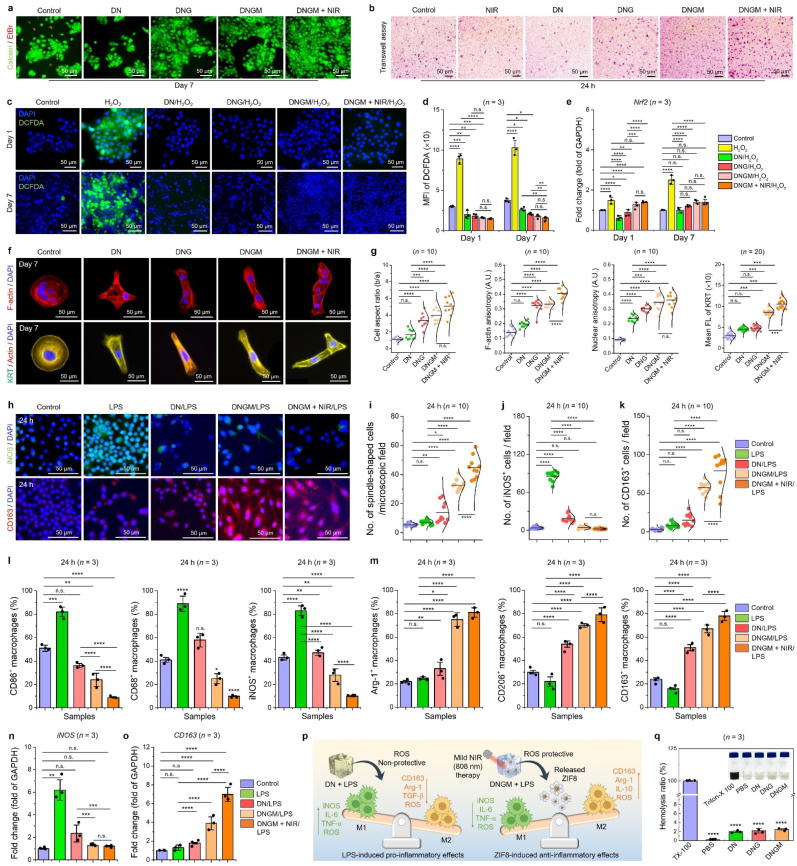
*In vitro* biocompatibility of the fabricated cryogel scaffolds. **(a)** Representative FL images showing the live/dead (Calcein-AM/EtBr) assay of HaCaT cells growing on various cryogel scaffolds after 7 days of culture (*n* = 3 each). Scale bar: 50 μm. **(b)** Transwell® cell migration assay of HaCaT cells showing the migrated colonies after 24 h of treatment (*n* = 5 each). Scale bar: 50 μm. **(c, d)** Representative DCF-DA staining results of HaCaT cells at day 1 and day 7 showing the excellent ROS scavenging properties (*n* = 3 each). Scale bar: 50 μm. **(e)** qRT-PCR analysis of the *Nrf2* gene expression as an evidence of redox homeostasis mechanism of the fabricated cryogels. **(f)** Immunostaining images of HaCaT cells showing the F-actin (red), nuclear (blue) morphology, and the expression of cytokeratin protein (green) marker after 7 days of culture. **(g)** Statistical data for cell aspect ratio, F-actin and nuclear anisotropy (=elongation index), and KRT FL intensity in various groups after 7 days of culture (*n* = 10 each). **(h, i)** Immunostaining results of macrophage polarization specific markers (iNOS and CD163) expression in LPS-induced RAW 264.7 cells in various formulations after 24 h of incubation. Scale bar: 50 μm. **(j, k)** Statistical data of the immunostaining experiment for RAW 264.7 cells (*n* = 10 each). **(l, m)** FACS quantification of RAW 264.7 cells showing the expression of M1 (CD86, CD68, and iNOS) and M2 (Arg-1, CD206, and CD163)-polarization specific markers at indicated time point (*n* = 3 each). **(n, o)** qRT-PCR validation of M1 and M2-polarization specific gene markers expression after 24 h of incubation with various groups (*n* = 3 each). **(p)** Schematic illustration showing the mechanism of mild phototherapy-assisted M2-macrophage polarization *in vitro*. **(q)***In vitro* blood biocompatibility (hemolysis assay) test of various cryogels adopted in this study. Data reported as mean ± s.d. of replicated experiments, statistical significance was considered at ∗*p* < 0.05, ∗∗*p* < 0.01, ∗∗∗*p* < 0.001, and ∗∗∗∗*p* < 0.0001 (One-way ANOVA followed by Tukey's HSD *post-hoc* test).

The *in vitro* biocompatibility of the fabricated cryogels was assessed using human keratinocyte (HaCaT) and murine monocyte/macrophage (RAW 264.7) cells. The water-soluble tetrazolium-8 (WST-8) and terminal deoxynucleotidyl transferase dUTP nick end labeling (TUNEL) assays were initially performed to evaluate the cytotoxicity and intracellular apoptosis of the developed cryogels w/or w/o NIR stimulation. Prior to cryogel biocompatibility test, we studied the cytotoxicity of the ZIF-8 particles (0–100 μM) using WST-8 assay to define a safe therapeutic window. As shown in Fig. S14(a–c), the fabricated ZIF-8 particles were non-toxic to the HDFs (viability >95 %), HaCaT (viability ∼99 %), and HUVECs (viability >90 %) up to 7 days, suggesting their superior biocompatibility. Moreover, the results of the WST-8 assay primarily suggest that none of the cryogels were toxic to the HaCaT cells after 7 and 14 days, w/o NIR (Fig. S14(d)). Notably, with mild NIR stimulation, we observed significantly higher viability on DNGM cryogels (∗*p* < 0.05 and ∗∗*p* < 0.01) from day 1 to day 14 (Fig. S14(e)) when compared with control, DN, and DNG groups, suggesting that mild phototherapy enhanced the proliferation of HaCaT cells. Moreover, the TUNEL assay further confirmed that none of the HaCaT cells undergone nuclear apoptosis (characterized by the green color of BrdU) at day 7 and day 14 w/or w/o NIR stimulation (Fig. S14(f and g)), which would be due to the release of Zn^2+^ from the DNGM, acting as a ROS attenuator and promoting differentiation [62]. To validate the regenerative capabilities of the fabricated cryogels, we performed the live/dead staining after 7 days of *in vitro* culture, and the results are shown in Fig. 3(a). Interestingly, the HaCaT cells were mostly found live growing onto the cryogels at day 7, including the control group. We observed an increase in cell number when we moved from the control to the DNGM + NIR group. The statistical analysis of the live/dead assay revealed a significant increase in cell number for DNGM (∼110 %, ∗∗*p* < 0.01) and DNGM + NIR (∼125 %, ∗∗*p* < 0.01) groups at day 7, confirming their bioactive properties (Fig. S15(a)).

Selective photobiomodulation (660–980 nm) has been5shown to exert a promising effect on skin cell migration via promoting cell proliferation, collagen synthesis, and scarless wound healing [63]. To evaluate the therapeutic efficacy of the fabricated cryogels, we investigated the HaCaT cell migration and morphogenesis after 7 days of treatment w/or w/o NIR. The cell migration assay was performed using a Transwell migration insert. The various formulation was introduced in the Transwell inserts with HaCaT cells, while the control group received no cryogel treatment. As shown in Fig. 3(b) and Fig. S15(b), the number of migrated HaCaT colonies accumulated higher (∗∗*p* < 0.01) than the control group. More interestingly, the HaCaT cells cultured with DNGM + NIR treatment exhibited a significantly (∗∗∗*p* < 0.001) higher migration of colonies towards the insert, suggesting that DNGM + light stimulation triggered the HaCaT migration. Thus, we conclude that the enhanced bioactivity and light-responsive property of the DNGM favored greater proliferation via Zn^2+^ uptake, and nutrient exchange, collectively enhancing HaCaT migration. Subsequently, we also verified the excellent ROS protective role of the fabricated cryogels on HaCaT cells at day 1 and day 7. As depicted in Fig. 3(c and d), the fabricated hydrogels (DN, DNG, and DNGM) displayed significantly (∗∗*p* < 0.01 and ∗∗∗∗*p* < 0.0001) lower fluorescence for DCF-DA after H_2_O_2_-induced oxidative stress compared to control and H_2_O_2_-treated HaCaT cells at day 1 and day 7, suggesting their excellent ROS attenuation properties. More surprisingly, the DNGM + NIR group also displayed no noticeable change in DCF-DA fluorescence. This was well-supported by the expression of the redox homeostasis-related genes, *e*.*g*., *Nrf-2* and *HO-1* as shown in Fig. 3(e) and Fig. S16. The mRNA expression for *Nrf2* and *HO-1* were significantly raised in H_2_O_2_-treated cells (>2.0 fold, ∗∗∗∗*p* < 0.0001), while in DN, DNG, DNGM, and DNGM + NIR groups, no significant change (∼1.2–1.4 fold) in their expression was observed compared to the control, suggesting that the fabricated cryogels are highly ROS protective in nature and would not elicit any toxicity towards HaCaT cells.

The immunostaining results of the HaCaT cells after incubating with various samples revealed attractive morphological changes at day 7. Initially, the cellular anisotropy in response to cryogel porosity and light stimulation was assessed using F-actin staining, while the keratinization was observed by staining with a basic cytokeratin (KRT) marker. As depicted in Fig. 3(c), the control group HaCaT cells displayed round cells with isotropic F-actin distribution. Notably, the cells cultured on DN, DNG, DNGM, and DNGM + NIR showed elongated and/or anisotropic F-actin and nuclear distribution, with a higher anisotropy for the DNGM + NIR group. It has been shown that scaffold porosity and stiffness greatly influence cell adhesion and spreading [64]. The statistical analysis of cell aspect ratio, F-actin, and nuclear anisotropic index showed that NIR stimulation through DNGM cryogel positively regulated the cell architecture. As shown in Fig. 3(d), the cell aspect ratio, F-actin, and nuclear anisotropy of the HaCaT cells significantly (∗∗∗∗*p* < 0.0001) increased as we move from control to DNGM and DNGM + NIR group, suggesting its bioactive and light-induced migration dynamics. The HaCaT cell aspect ratio, F-actin, and nuclear anisotropic index for the DNGM + NIR group were calculated to be 5.1, 0.4, and 3.6, respectively. This was also reflected in the intracellular KRT expression of HaCaT cells. The cells in all the groups showed profound expression of KRT with no significant difference in expression for the control, DN, and DNG groups. However, the KRT expression was found to be significantly higher in DNGM (∗∗∗*p* < 0.001) and DNGM + NIR groups (∗∗∗*p* < 0.001) than in the control, conferring the synergistic effect of bioactive DNGM with photobiomodulation.

The immunofluorescence analysis demonstrates that DNGM and DNGM + NIR treatments promote EMT, a key cellular process in wound healing. EMT is characterized by the loss of epithelial traits and the acquisition of mesenchymal features, enabling enhanced migration and tissue repair. In the presented data, vimentin, a hallmark mesenchymal marker, was strongly expressed in DNGM and DNGM + NIR groups compared to the control (Fig. S17(a)), suggesting cytoskeletal reorganization and increased cell motility. Similarly, RhoA GTPase, a regulator of actin cytoskeleton dynamics and cell migration, showed elevated expression under these treatments, supporting active remodeling of the cellular architecture (Fig. S17(b)). Importantly, YAP, a mechanotransduction-related protein and key EMT regulator, exhibited enhanced nuclear localization in the DNGM and DNGM + NIR groups, further validating EMT induction. When compared to TGFβ1 (a classical EMT inducer), DNGM and DNGM + NIR demonstrated comparable effects, highlighting their potential to trigger EMT without additional growth factor stimulation. Notably, the synergistic effect of NIR with DNGM appeared to strengthen EMT activation, as evidenced by increased expression of these markers (Fig. S17(c)). The statistical analysis of the immunofluorescence staining is given in Fig. S17(d). Altogether, these findings indicate that DNGM and DNGM + NIR treatments effectively drive EMT, thereby facilitating cell migration and tissue remodeling processes that are crucial for accelerated wound healing.

The proteomic and genomic profile of the HaCaT cells in various formulations was investigated using Raybiotech® human cytokine array, enzyme-linked immunosorbent assay (ELISA), and real-time PCR (qRT-PCR) analysis after 7 days of culture. Before the proteomic study, the bulk secretome of the HaCaT cells from different groups was examined for quality control of the secreted proteins. As shown in Fig. S17(e), the SDS-PAGE identified distinct protein bands in the 20–242 kDa range, with a thick band at 242 kDa for the DNGM and DNGM + NIR groups. Nevertheless, the HaCaT cells from all the groups showed a similar banding pattern. The Raybiotech antibody array identified 9 key proteins from the HaCaT secretome, including growth-regulated oncogene-alpha (RGO-α), interleukin-8 (IL-8), angiogenin (ANG), tissue inhibitor of metalloproteinase-1 (TIMP-1), osteoprotegerin (OPG), transforming growth factor beta-1 (TGFβ-1), epidermal growth factor (EGF), monocyte chemoattractant protein-2 (MCP-2), and insulin-like growth factor protein-4 (IGFBP-4), respectively. The array maps of the respective groups are shown in Fig. S17(f). It is worth noticing that compared to the control group; all the protein spots are predominant in the treatment groups (Fig. S17(g)) with a significantly (∗∗∗*p* < 0.001) higher expression of OPG and EGF in DNGM (1.2 and 1.3 fold) and DNGM + NIR (1.5 and 1.7 fold) groups, suggesting that MXene@ZIF8 incorporated DNA cryogels induced higher rate of secretion from HaCaT cells [65]. Surprisingly, in the DNGM + NIR group, the expression of ANG and TGFβ-1 was also higher than in other groups. A STRING protein-protein interaction study (Fig. S17(h) and Table S2) through *k*-means clustering revealed that the secreted proteins are mostly connected to IL-10 signaling (Cluster-1 and 2; MCP2, IL-8, GRO- α, ANG, and TIMP-1) and epidermal growth factor signaling (Cluster-3; EGF) towards keratinization [66].

The involvement of EGF secretion and signaling in HaCaT cells, we next performed an ELISA test to verify the intracellular EGF level in various groups. As shown in Fig. S17(i), the EGF concentration was found significantly higher in DNGM (158 pg mL^−1^, 30 times, ∗∗∗∗*p* < 0.0001) and DNGM + NIR (204 pg mL^−1^, 40 times, ∗∗∗∗*p* < 0.0001) groups compared to DN (18 pg mL^−1^) or control (<10 pg mL^−1^) group, indicating that photobiomodulation through DNGM triggered the intracellular EGF production in HaCaT cells, which would be beneficial for wound healing. Keratinocytes express a variety of transcription factors, *e.g*., KRTs, which play an essential role in skin homeostasis, epidermis maintenance, keratinization, and inflammation [67]. To explore the potential of the fabricated cryogels in keratinization and skin homeostasis, we investigated the key epidermis maintenance gene markers (*KRT5*, *KRT10*, and *KRT14*) expression by qRT-PCR after 7 days of HaCaT culture in differentiation media (DMEM supplemented with 5 mM CaCl_2_) with various formulations. As depicted in Fig. S17(j–l), the pure DNA gel (=DN) exhibited scanty expression for all the 3 biomarkers compared to the control, with a significantly high expression for *KRT10* (∼1.2 fold, ∗∗∗∗*p* < 0.0001) at day 7. Interestingly, we observed a higher expression profile for *KRT5* (>1.2 fold, ∗*p* < 0.05), *KRT10* (1.5 fold, ∗∗∗∗*p* < 0.0001), and *KRT14* (>1.5 fold, ∗∗∗∗*p* < 0.0001) in DNG, DNGM, and DNGM + NIR groups at day 7. The flow cytometric analysis further verified that DNGM and DNGM + NIR groups have a higher number of KRT5^+^ (Fig. S18(a and b)) and KRT14^+^ (Fig. S18(c)) cells at day 7 after HaCaT differentiation, confirming the bioactive role of DNGM and mild phototherapy. Taken together, these findings underscore the remarkable bioactive properties of the DNGM cryogel and photobiomodulation through MXene@ZIF8 collectively enhanced HaCaT cell migration, differentiation, and keratinization, which could be beneficial for wound healing.

### 2.4 DNGM-induced macrophage reprogramming under mild phototherapy

Light-responsive hydrogels have been shown to have significant effects on macrophage polarization [68]. In particular, the low-intensity NIR light stimulation exerts anti-inflammatory effects by inducing the activity of M2 macrophage phenotypes and reducing the pro-inflammatory M1 types [69]. Moreover, it has been shown that photoactivated hydrogels integrated with MXenes or Zn^2+^ ions can ameliorate healing by activating the macrophage polarization toward the M2 phenotype and promote wound healing [[70], [71], [72]]. Consistent with these reports, we hypothesized that the photoactivated MXene@ZIF8 in bioactive DNGM cryogel would deploy an anti-inflammatory microenvironment during wound healing. Consequently, we tested the RAW 264.7 cell polarization using the fabricated cryogel in response to an inflammatory agent, lipopolysaccharide (LPS), w/or w/o NIR stimulation. The immunostaining results on LPS-induced RAW 264.7 cells after 24 h of culture exhibited an interesting pattern of pro-inflammatory (intrinsic nitric oxide synthase or iNOS) and anti-inflammatory (cluster of differentiation-163 or CD163) protein markers expression. As shown in Fig. 3(h), the control group (-LPS) cells exhibited negligible fluorescence for iNOS and CD163, suggesting no polarization state. The LPS-treated RAW 264.7 cells displayed higher cytoplasmic fluorescence for iNOS and less fluorescence for CD163, followed by a low intensity of iNOS and moderate intensity of CD163 for the DN/LPS group. Interestingly, the DNGM/LPS group exhibits a very low amount of iNOS^+^ cells and higher cytoplasmic fluorescence for CD163, suggesting a shift towards the M2 phenotype. More interestingly, when the RAW 264.7 cells growing on DNGM/LPS were treated with NIR light, we observed a negligible fluorescence for iNOS and a significantly higher cytoplasmic fluorescence for CD163. The characteristic M2 polarization was also followed by the elongated (=spindle-shaped) fibroblast-like morphology of RAW 264.7 cells upon NIR stimulation, with a significantly (∗∗∗∗*p* < 0.0001) higher number of spindle-shaped cells in the DNGM + NIR/LPS group than in other groups (Fig. 3(i)). The statistical analysis of the immunostaining experiment also suggests that the number of iNOS^+^ cells gradually decreased when we moved from control to DNGM + NIR/LPS (Fig. 3(j)). In contrast, the number of CD163^+^ cells significantly (∗∗∗∗*p* < 0.0001) increased after 24 h of *in vitro* culture (Fig. 3(k)). To validate this, we additionally performed the effect of DNGM/LPS and DNGM + NIR/LPS on macrophage polarization up to 14 days of culture. Notably, there was a significant shift in F-actin morphology (cytoskeleton) from round/oval-shaped to spindle-shaped (=fibroblastic, typically M2 morphology) when we move from control (untreated) or LPS-treated to DNGM + NIR/LPS group at day 7 and day 14 (Fig. S19(a)). The percentage of elongated cells were significantly (∗∗∗∗*p* < 0.0001) increased from control/LPS → DNGM + NIR/LPS group (Fig. S19(b and c)), suggesting the long-term M2 polarization potential of the DNGM cryogels. To support this data, we then investigate the intracellular expression level of iNOS and CD163 via immunostaining. Surprisingly, the control groups at day 7 and day 14 expressed a low level of iNOS and CD163 and a high level of cytoplasmic iNOS expression in LPS-treated cells. More interestingly, the expression of iNOS was decreased and CD163 expression was gradually increased when we move from DN/LPS to DNGM + NIR/LPS group, suggesting that bioactive DNGM with mild phototherapy positively enhanced the M2 polarization in an immunocompromised environment (Fig. S19(d)). The statistical analysis also suggested a significantly (∗∗∗∗*p* < 0.0001) higher number of CD163^+^ cells in the cryogel-treated groups than control or LPS groups at day 7 and day 14 (Fig. S19(e and f)).

Next, we then studied the immunoregulatory profile in RAW 264.7 cells by FACS and qRT-PCR. The gating strategies for FACS analysis is given in Fig. S20. As depicted in Fig. 3(l and m), the flow cytometry results suggest that the LPS and DN/LPS-treated samples have mostly CD86^+^ (40–80 %), CD68^+^ (50–90 %), and iNOS^+^ (45–80 %) cells, while lesser amount of Arg-1^+^ (20–30 %), CD206^+^ (30–50 %), and CD163^+^ (20–40 %) cells. In contrast, the DNGM/LPS and DNGM + NIR/LPS groups have a higher number of Arg-1^+^ (70–80 %), CD206^+^ (∼80 %), and CD163^+^ (70–80 %) than the control and other groups. The qRT-PCR study also revealed a similar gene expression pattern for RAW 264.7 cells. The control group LPS-treated group displayed significantly higher gene expression for *iNOS* (>5.0 fold, ∗∗*p* < 0.01), followed by a drastic decrease (≤ 2-fold, *n*.*s*. to control) when we moved from DN/LPS to DNGM + NIR/LPS groups (Fig. 3(n)). Notably, the expression of the *CD163* gene marker was found to be significantly increased when we moved from the LPS (≤ 2-fold, n.s. to control) to DNGM + NIR/LPS (>6.0 fold, ∗∗∗∗*p* < 0.0001) group (Fig. 3(o)), conferring the suppression of M1 phenotype and activation of M2 phenotype. Furthermore, the qRT-PCR results of RAW 264.7 cells at day 7 and day 14 also showed a remarkable suppression of *iNOS* gene (<1.0 fold) and significant up-regulation of *CD163* gene (>30.0 fold, ∗∗∗*p* < 0.001 and ∗∗∗∗*p* < 0.0001) expression in DNGM + NIR/LPS group, indicating the long-term M2-polarization potential (Fig. S21(a–d)). The activation of M2 polarization or anti-inflammatory activation was probably due to the activation of NIR and MXene@Zn^2+^-induced co-activation of M2 transcription factors, which later triggered the activation of chemokine ligand-17 (CCL-17), CCL22, TGFβ-1, IL-10 via peroxisome proliferator-activated receptor (PPAR) and Janus kinase/signal transducers and activators of transcription (JAK-STAT) signaling and downregulating the tumor necrosis factor-alpha (TNF-α) and IL-1β signaling pathways, thereby promoting wound healing [69,71]. Taken together, our findings suggest that DNGM mitigates the pro-inflammatory activation of macrophages via reducing intracellular ROS and the unique cryogel porosity, which helped in the spreading and differentiation of RAW 264.7 cells towards a tissue healing phenotype. A schematic illustration showing the DNGM cryogel-assisted macrophage polarization mechanism with photobiomodulation is given in Fig. 3(p). We also tested the hemocompatibility of the fabricated cryogels using red blood cells (RBCs). Strikingly, none of the cryogel samples were found toxic to the RBCs (Fig. 3(q)), which was characterized by the low hemolysis rate, indicating their excellent blood biocompatibility.

### 2.5 Transcriptomic study reveals signature DEGs involved in wound healing

Based on the outstanding biocompatibility and skin regenerative properties of the DNGM cryogel under photobiomodulation, we aimed to investigate the molecular mechanisms underlying the HaCaT cell proliferation and keratinization using bulk RNA sequencing (RNA-Seq) analysis. Nevertheless, the excellent biocompatibility of the HaCaT cells towards DNGM is well-elucidated by proteomic and genomic assays; however, the exact molecular cues regulating the keratinization and wound healing are still unknown. To address this, we performed the RNA-Seq after culturing HaCaT cells in differentiation media containing cryogels. We selected control, DN, DNGM, and DNGM + NIR groups to uncover the differentially expressed genes (DEGs) after 7 days of culture *in vitro*. Initially, we performed the hierarchical clustering and correlation mapping of the DEGs, followed by bioinformatics analysis to find out key enrichment in various pathways, respectively.

**Fig. 4:**
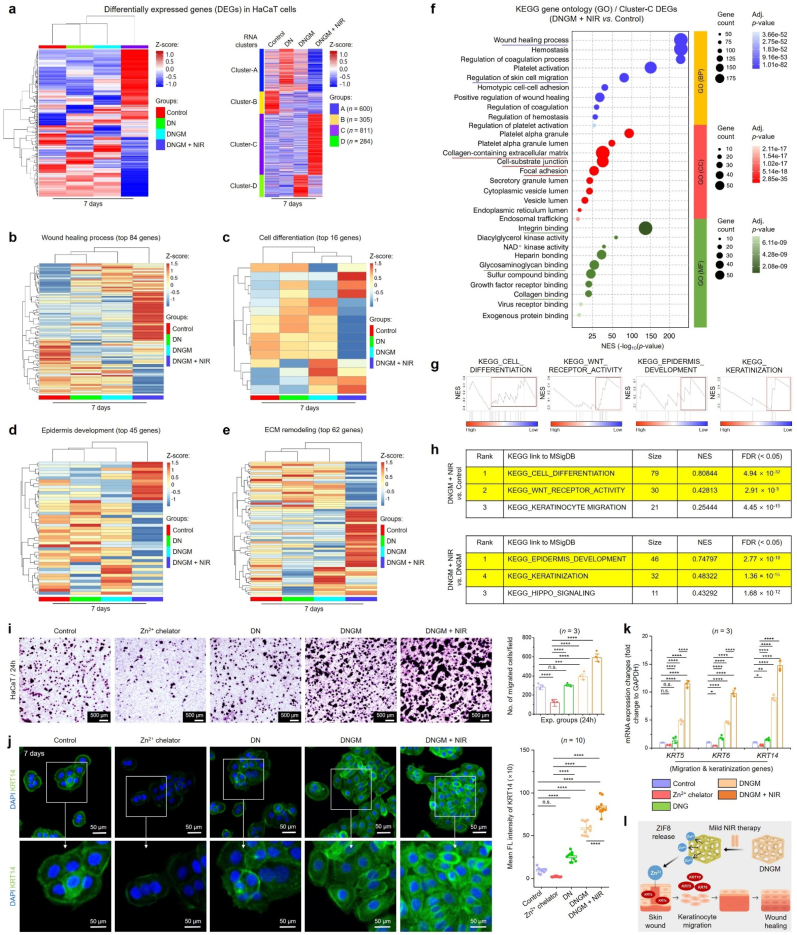
Transcriptomic analysis of HaCaT cells shows upregulation in the wound healing process. **(a)** Heatmaps of the differentially expressed genes (DEGs) and *k*-means clustering results of HaCaT cells in various groups after 7 days of treatment. **(b)** KEGG gene ontology (GO) analysis of DEGs in Cluster-C showing the enrichment in various processes when compared between DNGM + NIR *vs*. Control group after 7 days of treatment. **(c**–**f)** Heatmaps of the top DEGs in HaCaT cells associated with wound healing, cell differentiation, epidermis development, and ECM remodeling after 7 days of treatment. **(g, h)** Gene set enrichment analysis (GSEA) and molecular signatures database (MSigDB) analysis show the top 3 ranked gene sets in DNGM + NIR *vs*. Control and DNGM + NIR *vs*. DNGM groups. **(i)** Validation of the RNA-Seq data to examine the role of DNGM and DNGM + NIR-induced cell migration and keratinization in HaCaT cells *in vitro*. Representative optical micrographs with corresponding statistical analysis of the Transwell migration assay in the presence of Zn^2+^ chelator (DETPA) and cryogel samples. Scale bar: 500 μm. **(j)** CLSM images of HaCaT cells showing the expression of KRT14 at day 7 in various groups. Scale bar: 50 μm. **(k, l)** qRT-PCR validation of cell migration and keratinization-specific gene markers expression in HaCaT cells after 7 days of culture. Data reported as mean ± s.d. of replicated experiments, statistical significance was considered at ∗*p* < 0.05, ∗∗*p* < 0.01, ∗∗∗*p* < 0.001, and ∗∗∗∗*p* < 0.0001 (One-way ANOVA followed by Tukey's HSD *post-hoc* test).

The unbiased and *k*-means hierarchical clustering results showed a significant (∗*p* < 0.05) amount of up-regulated DEGs in the DNGM + NIR group than other groups (Fig. 4(a)). Subsequently, out of a total of 2000 DEGs, the Cluster-C of the DNGM + NIR group exhibited significantly up-or down-regulated DEGs (gene count: 811, Log2FC, ∗*p* < 0.05) than other groups, suggesting that photobiomodulation through DNGM significantly up-or down-regulated the HaCaT transcriptome. We also identified the top genes involved in the wound healing process, cell differentiation, epidermis development, and ECM remodeling process. Transcriptomic analysis revealed a total of 84, 16, 45, and 62 genes significantly up-or down-regulated in HaCaT cells after 7 days of induction through fabricated cryogels (Fig. 4(b–e)). Next, we performed GSEA to detect subtle yet coordinated changes in DEG expression in the DNGM + NIR *vs*. control group from HaCaT transcriptome on day 7. This was also supported by the scatter plots and Venn diagrams, which show inter-relationships between the DEGs in various groups. As shown in Fig. S22(a and b), control *vs*. DN and control *vs*. DNGM display no significant change in DEG expression. Surprisingly, a significant change in up- and down-regulated DEGs was found when comparing control and DNGM + NIR (Fig. S22(c)). More surprisingly, we noticed a highly significant change in DEG expression with a higher variance (R^2^ > 0.99, Log2FC) in the DN *vs*. DNGM + NIR and DNGM *vs*. DNGM + NIR group (Fig. S22(d and e)), respectively. We concentrated on finding the overlapping DEGs (total up/down-regulated and contra-regulated) in various groups using the Venn diagram, and the result is shown in Fig. S23. The statistical analysis reveals a diverse expression pattern of DEGs in various groups. We found a total of 42 and 49 genes up- and down-regulated in the control/DN (G-1) group, while 37 and 43 genes were found up-and down-regulated in the control/DNGM (G-2) group, respectively. Meanwhile, only 1 and 7 genes were found up- and down-regulated, with no contra-regulated genes when comparing control/DN *vs*. control/DNGM. Similarly, in the control/DNGM + NIR (G3) group, 1095 and 656 genes were found up- and down-regulated, and among them, 20 and 14 genes were found co-expressed when compared with the control/DN group, and only 1 gene was found to be contra-regulated. Notably, among the 20 up-regulated DEGs highly up-regulated in the DNGM + NIR group were mostly the nuclear transcription factors, and two of them were identified as zinc-binding proteins, *i.e.*, *Znf648* and *Znf789*, which were previously known as activation of Zn^2+^ ions for cell proliferation, adhesion, and cytoskeletal remodeling [73]. Next, we compared the DEG expression between control/DNGM and control/DNGM + NIR groups, revealing a co-expression of 35 and 14 up-and-down-regulated genes with no contra-regulated genes. Strikingly, when we compared co-expression and co-regulatory DEGs in G-1, G-2, and G-3, we found significant up-regulation of 6 genes with no down- and contra-regulated genes. Out of the 6 identified genes, 3 genes, i.e., *Znf648*, *Znf566*, and *Znf620,* were found to be associated with Zn^2+^ uptake events.

The gene ontology (GO) and gene sets enrichment analysis (GSEA) in DNGM + NIR *vs*. control of the Cluster-C DEGs (811 genes) from HaCaT cells at day 7 revealed the enrichment of several enrichment terms associated with keratinization and wound healing (Fig. 4(f)). In particular, terms such as ‘*wound healing process*’ (gene count: 175) and ‘*regulation of skin cell migration*’ (gene count: 125) were found to be highly enriched in biological process (BP), while a higher enrichment was found for ‘*collagen-containing extracellular matrix*’ (gene count: 50), ‘*cell-substrate junction*’ (gene count: 40), and ‘*focal adhesion*’ (gene count: 32) terms in cellular component (CC), respectively. Similarly, in molecular function (MF), several terms, such as ‘*integrin binding*’ (gene count: 48), ‘*glycosaminoglycan binding*’ (gene count: 30), and ‘*collagen binding*’ (gene count: 19), were found significantly enriched in DNGM + NIR group, suggesting its positive role in wound healing. The individual DEGs involved in BP, CC, and MF are represented as a Cnet plot, and the data is shown in Fig. S24–26. More interestingly, the KEGG pathway enrichment result of the DNGM + NIR *vs*. control group showed higher enrichment for 4 key pathways, *i*.*e*., Rap1 signaling (P-1), hippo signaling (P-2), focal adhesion (P-3), and ECM-receptor signaling (P-4) pathways (Figs. S27–30) as predicted through Pathview tool. The significantly (∗*p* < 0.05) up-regulated genes in P-1, P-2, P-3, and P-4 were identified as *Vav2*, *Wnt10b*, *Yap1*, *MLC1*, and *CD36*, respectively. An in-depth text and data mining through bioinformatics tools revealed that these genes play a positive role in hemostasis, cell migration and invasion, skin cell proliferation, keratinization, reducing inflammation, hair follicle promotion, and wound healing [74,75]. To validate this, we performed a qRT-PCR analysis of the above-mentioned genes in HaCaT cells after 7 days of culture *in vitro*. As depicted in Fig. S31, the mRNA expression profile was found higher for DNGM, with a significantly higher expression for *Vav2* (>2.0 fold, ∗∗*p* < 0.01), *Wnt10b* (>15.0 fold, ∗∗∗∗*p* < 0.0001), *Yap1* (>20.0 fold, ∗∗∗∗*p* < 0.0001), *MLC1* (>3.0 fold, ∗∗∗*p* < 0.001), and *CD36* (>3.0 fold, ∗∗∗∗*p* < 0.0001) in DNGM + NIR group than control and other groups, conferring that photobiomodulation through DNGM triggers cell adhesion, proliferation, and differentiation of HaCaT cells towards wound healing.

Furthermore, a GSEA linked to molecular signature database (MSigDB) and principal component analysis (PCA) was carried out to identify the hallmark gene sets and gene variance In various groups from HaCaT transcriptome at day 7 using GSEA (v4.3.3) web-based tool and ggplot2 (RStudio, v2024). As shown in Fig. 4(g and h), the top four hallmarks were identified according to their rank, gene size, normalized enrichment score (NES), and false discovery rate (FDR) < 0.05, respectively. In particular, we found the highest score for ‘*cell differentiation*’ (NES: 0.80844), ‘*Wnt receptor activity*’ (NES: 0.42813), and ‘*keratinocyte migration*’ (NES: 0.25444) when comparing DNGM + NIR *vs*. the control group. More interestingly, the top 3 ranked terms, such as ‘*epidermis development*’ (NES: 0.74797), ‘*keratinization*’ (NES: 0.60322), and ‘*Hippo signaling*’ (NES: 0.43292), were observed in MSigDB, underscoring the significant role of DNGM and photobiomodulation in keratinization and wound healing. To support these findings, we then concentrated on evaluating the over-representation analysis (ORA) of the gene sets in the DNGM + NIR *vs*. control group using g:Profiler linked with the Ensembl database. The gene sets were cumulatively compared based on GO, KEGG, Reactome (REAC), and human protein atlas (HPA) databases. As shown in Fig. S32, the genes (=DEGs) that are highly expressed in DNGM + NIR group showed significantly higher (-Log_10_(*p*)) enrichment in 3590 GO pathways, including cell surface protein binding (2 genes), regulatory RNA binding (18 genes), Zn^2+^ ion binding (7 genes), cell morphogenesis (22 genes), epidermis development (27 genes), growth factor secretion (37 genes), skin cell differentiation (28 genes), keratinization (38 genes), and cellular response to biomechanical stimuli (31 genes), respectively. Similarly, the KEGG identified significant enrichment in glycoprotein complex formation (53 genes) and TGF-beta-Akt signaling (57 genes), while the REAC and HPA chiefly identified protein metabolism (206), DNA binding (70), fibroblast maturation (22), and skin gland formation (58)-related terms at day 7. These results envisioned the skin regenerative and wound healing capabilities of the DNGM cryogel under photobiomodulation.

The PCA plot shows that the DNGM + NIR group showed the highest variance in DEGs expression and clustered separately from the control group (Fig. S33(a)) while closely associated with the DN and DNGM groups, suggesting that DNA and MXene@ZIF8 containing cryogels with NIR stimulation has a positive effect on HaCaT transcriptome. The signature up-regulated DEGs associated with wound healing (*Adra2c*, *Cd36*, *Fgf1*, *Gna12*, *Gp6*, *Fn1*, *Hsp6*, *Mpig6b*, *Slc7a11*, and *Tlr4*), keratinization (*Mfab*, *Krt1*, *Krt5*, *Krt10*, *Krt14*, *Flg*, *Dsg4*, and *Krt77*), and angiogenesis (*Aggf1*, *Efnb2*, *Hoxa7*, *Micall1*, *Adgra2*, *Adm2*, *Efnb1*, *Egf*, *Epgn*, *Pik3r3*, and *Tmem201*) are also identified and their expression profiles (Log2FC, ∗*p* < 0.05) are shown in Fig. S33(b–d). Moreover, the multidimensional PCA revealed notable findings related to ECM remodeling for the DNGM + NIR group. As depicted in Fig. S34, we observed higher enrichment for several terms, including ‘*animal organ morphogenesis*’ (*p* = 2e-08), ‘*focal adhesion*’ (*p* = 5e-04), ‘*cell-substrate junction*’ (*p* = 4e-04), ‘*zinc ion binding*’ (*p* = 4e-05) in KEGG (Fig. S34(a–c)), while ‘*TGFb signaling*’ (*p* = 3e-01), ‘*PI3K-Akt-mTOR signaling*’ (*p* = 3e-02), ‘*Wnt signaling*’ (*p* = 5e-01), and ‘*hair follicle development: cytodifferentiation signaling*’ (*p* = 2e-01) in Wiki pathways (Fig. S34(d)), which confirms our clustering, GSEA, and qRT-PCR data.

To justify the effect of Zn^2+^ and/or *Znf* proteins in keratinization and wound healing, we further performed a chelation and knockdown experiments using HaCaT cells. The chelation experiment was performed using diethylenetriaminepentaacetic acid (DETPA, 100 μM), followed by Transwell™ migration and ICC/qRT-PCR validation, while the knockdown experiment was conducted using miR-*Znf648*, respectively. As depicted in Fig. 4(i), the DETPA significantly (∗∗∗∗*p* < 0.0001) hindered the HaCaT cell migration after 24 h than control. Besides, a remarkable increased in the migrated HaCaT colonies were observed in DNGM and DNGM + NIR-treated groups, conferring the role of released Zn^2+^ ions in cell migration. This was well-supported by the intracellular KRT14 expression in HaCaT cells at day 7 (Fig. 4(j)). Compared to the control group, the DEPTA group exhibited significant (∗∗∗∗*p* < 0.0001) reduction in KRT14 expression, while a significantly (∗∗∗∗*p* < 0.0001) higher expression was spotted for DNGM and DNGM + NIR groups. More interestingly, the mRNA expression for *KRT5* (>10.0 fold), *KRT6* (>8.0 fold), and *KRT14* (>12.0 fold) were significantly increased compared to the DETPA group, suggesting that Zn^2+^ treatment and transport through DNGM is crucial for HaCaT cell migration and keratinization. Furthermore, the qRT-PCR results also demonstrate that miR-Znf648 treatment markedly suppressed the expression of keratinocyte markers *KRT5*, *KRT6*, and *KRT14*, indicating its inhibitory role in keratinocyte differentiation (Fig. S35(a)). In contrast, DNGM and particularly DNGM + NIR groups significantly upregulated these markers, highlighting that the cryogel system can override miR-Znf648–mediated suppression and strongly promote epidermal regeneration. This was also reflected in the 3D angiogenesis experiment of HUVECs *in vitro*. As indicated in Fig. S35(b), the HUVECs grown on Matrigel control showed numerous sprouts, while the DETPA-treated groups exhibited impaired sprouting after 24 h. A significant (∗∗∗∗*p* < 0.0001) increase in sprouts number was observed in DNGM and DNGM + NIR groups after 24 h, suggesting that Zn^2+^ is pivotal for angiogenesis of HUVECs [76]. Taken together, these findings envision that DNGM cryogel has a positive effect on keratinization, angiogenesis, wound ECM remodeling, inducing secretion, reducing inflammation, promoting hair follicle/gland formation, and photobiomodulation through DNGM synergistically elevated the wound healing process by inducing zinc-binding proteins (*e.g*., *Znf*) through MXene@ZIF8 and thereby restoring skin function.

### 2.6 *In vivo* biosafety analysis of the fabricated cryogels

**Fig. 5:**
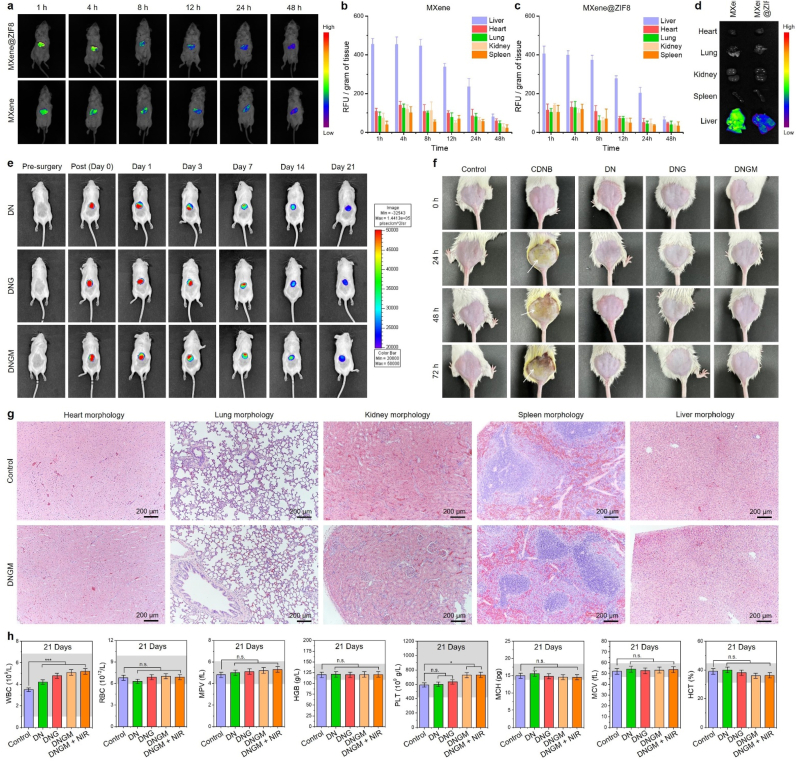
*In vivo* biosafety analysis of the fabricated cryogels. **(a)***In vivo* biodistribution analysis of the MXene and MXene@ZIF8 at indicated time points. **(b, c)** Statistical analysis of the relative fluorescence unit (RFU) in various organs at indicated time points. **(d)** Biodistribution of MXene and MXene@ZIF8 in major organs (heart, lung, kidney, spleen, and liver) after 24 h of administration. **(e)***In vivo* biodegradation of the fabricated cryogels up to 21 days. Each cryogel was loaded with 0.01 mg/mL FITC for IVIS analysis. **(f)** Digital photographs of the rats showing the skin irritant toxicity in the presence of fabricated cryogel samples at indicated time points. 1- chloro-2, 4-dinitrobenzene (CDNB) was used as a positive control for inducing skin allergy (white arrowhead). **(g)** H&E staining results showing the histology of major organs after 21 days of cryogel implantation. Scale bar: 200 μm. **(h)** Blood biochemistry analysis of the rats after treated with various formulations at day 21. Grey lines represent the clinical range for each components. Data reported as mean ± s.d. of replicated experiments, statistical significance was considered at ∗*p* < 0.05 and ∗∗∗*p* < 0.001 (One-way ANOVA with Tukey's HSD post-hoc test).

The *in vivo* biosafety evaluation of the fabricated cryogels was systematically assessed using biodistribution, biodegradation, histopathology, and hematological parameters (Fig. 5). Whole body fluorescence imaging revealed a time-dependent biodistribution pattern of MXene and MXene@ZIF8 following administration (Fig. 5(a)). Initially, strong signals were observed within the first 1–8 h, which gradually decreased over 48 h, indicating effective systemic clearance. Quantitative analysis demonstrated that the liver and spleen were the primary accumulation sites, consistent with reticuloendothelial system uptake (Fig. 5(b, c)). Importantly, MXene@ZIF8 exhibited faster clearance and reduced retention compared to bare MXene, suggesting that the ZIF8 coating improved biocompatibility and excretion kinetics. Furthermore, ex vivo organ fluorescence imaging at 24 h further confirmed minimal residual accumulation of MXene@ZIF8 relative to unmodified MXene (Fig. 5(d)). The IVIS imaging of fluorescein isothiocyanate (FITC)-labeled cryogels demonstrated gradual signal decline, confirming progressive biodegradation *in vivo* up to 21 days (Fig. 5(e)). The fluorescence intensity notably diminished after day 14, and almost complete disappearance was observed by day 21, indicating that the cryogels were biodegradable within a physiologically relevant timeframe. Such degradation kinetics are advantageous for wound healing or tissue engineering applications, as they minimize long-term foreign body response.

To assess irritant effects, the cryogels were subcutaneously implanted and monitored up to 72 h (Fig. 5(f) and Table S3). Unlike the CDNB positive control, which induced visible allergic erythema, no apparent redness, swelling, or ulceration was observed in cryogel-treated animals. This absence of skin irritation highlights the material's compatibility for local application. H&E staining of major organs (heart, lung, kidney, spleen, liver) after 21 days showed normal histomorphology with no necrosis, inflammatory infiltrates, or structural abnormalities in the cryogel-treated groups compared to the control (Fig. 5(g)). This further supports the systemic biosafety of the materials. Blood biochemistry at day 21 revealed no significant differences in WBC, RBC, HGB, PLT, MCV, MCH, or HCT values between the treated and control groups (Fig. 5(h)). This absence of hematological abnormalities confirms that the cryogels did not induce systemic toxicity or impair hematopoietic function. These results collectively demonstrate that the MXene@ZIF8-incorporated cryogels exhibit favorable *in vivo* biosafety, with efficient clearance, biodegradability, absence of skin irritation, preserved organ histology, and stable hematological indices. Such profiles position them as promising candidates for safe biomedical applications.

### 2.7 Infection-free and scarless wound healing programmed by DNGM cryogel under photobiomodulation

**Fig. 6:**
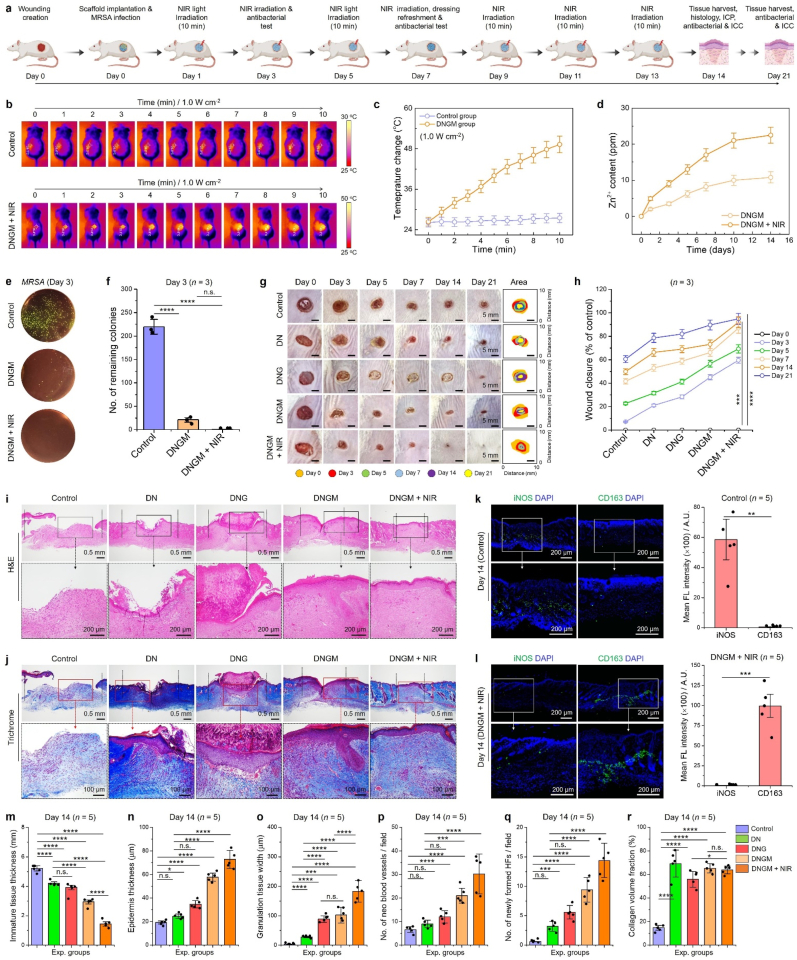
*In vivo* wound healing analysis of the fabricated cryogel scaffolds. **(a)** Schematic illustration of the wound healing timeline adopted in this study. **(b)** Representative thermal images of the DNGM cryogel after implantation in rat subcutaneous wound model when irradiated with 808 nm NIR light (1.0 W cm^−2^, 10 min). **(c)** Representative temperature rise profile of the DNGM cryogel as a function of time. **(d)** The Zn^2+^ release profile from the DNGM cryogel w/or w/o NIR irradiation at wound tissue at indicated time points. **(e)** Digital photographs of the MRSA colony isolated from the wound bed after irradiating with NIR (1.0 W cm^−2^, 10 min) on day 3. **(f)** Statistical analysis of the colony formation test. **(g)** Digital photographs of the macroscopic wound healing with the corresponding wound area in various groups. Scale bar: 5 mm. **(h)** Statistical analysis for % of macroscopic wound closure at indicated time points (*n* = 3 each). **(i, j)** Representative H&E and Trichrome staining images of the wound bed showing the microscopic wound healing potential of the fabricated cryogels at day 14. Scale bar: 100 μm, 200 μm, and 0.5 mm. **(k, l)** Immunostaining images of the wound tissue showing the expression of pro-inflammatory (iNOS) and anti-inflammatory (CD163) markers expression in control and DNGM + NIR groups at day 14 (*n* = 5 each). Scale bar: 200 μm. **(m**–**r)** Statistical analysis of the microscopic wound healing parameters (immature tissue thickness, epidermis thickness, granulation tissue width, no. of new blood vessels, hair follicles, and collagen fraction volume) at day 14 (*n* = 5 each). Data reported as mean ± s.d. of replicated experiments, statistical significance was considered at ∗*p* < 0.05, ∗∗*p* < 0.01, ∗∗∗*p* < 0.001, and ∗∗∗∗*p* < 0.0001 (One-way ANOVA followed by Tukey's HSD *post-hoc* test).

Inspired by the promising biocompatibility, photothermal property, antibacterial efficacy, skin regenerative capability, and ECM mimicking nature of the DNGM cryogel, we aimed to investigate the *in vivo* therapeutic potential using a mice subcutaneous infected wound model. The timeline of the wound treatment procedure and analysis is shown in Fig. 6(a). The mice were divided into five random groups: control (no cryogel, *n* = 10), DN (*n* = 10), DNG (*n* = 10), DNGM (*n* = 10), and DNGM + NIR (*n* = 10) groups, respectively. The macroscopic wound healing was monitored for up to 21 days, and the histological stainings were conducted on day 7 and day 14, as the subcutaneous wound heals typically between 10 and 14 days, followed by complete skin re-epithelialization and ECM remodeling by 21 days. Therefore, the regenerative potential was assessed using ICC and qRT-PCR after 14 and 21 days of cryogel implantation. The bacterial inoculation (*MRSA*, 10 μL, 1 × 10^8^ CFU mL^−1^) was performed on day 0 after cryogel treatment, and the NIR stimulation (808 nm, 1.0 W cm^−2^) was performed every other day. Subsequently, the Zn^2+^ ion release from the cryogel to wound tissue was also monitored using ICP-OES. As shown in Fig. 6(b), the DNGM cryogel sufficiently produced heat in the wound area, while the control group exhibited no change in temperature in the wound bed, suggesting that the photothermal heat production was mainly due to the incorporated photo-modulating agent, *i*.*e*., MXene@ZIF8 [23,29,41,77]. The time-dependent change in temperature rise is shown in Fig. 6(c). Notably, the Zn^2+^ release profile showed that the DNGM + NIR group released more Zn^2+^ than the DNGM group, suggesting that mild phototherapy through DNGM cryogel can effectively deliver Zn^2+^, activating intracellular Znf proteins. The greater release of Zn^2+^ under NIR will also benefit from higher antibacterial performance. The slow release of Zn^2+^ from the pure DNGM would be due to the shrinkage of the cryogel inside the wound due to body temperature. We observed a remarkable decrease in MRSA colonies following DNGM application with no visible colony for the DNGM + NIR group, underscoring that NIR stimulation through DNGM cryogel significantly (∗∗∗∗*p* < 0.0001) inhibited the *MRSA* growth (Fig. 6(e and f)). Moreover, this was also reflected in long-term antibacterial performance of the DNGM cryogel. As shown in Fig. S36, both DNGM and DNGM + NIR groups exhibited superior bactericidal effects against *MRSA* compared to the control group up to 21 days of *in vivo* implantation, suggesting their therapeutic efficacy.

The macroscopic wound healing results show the promising ECM mimicking and infection-free skin regeneration potential of the fabricated DNGM cryogel after 21 days of treatment. As depicted in Fig. 6(g), the control group exhibited a lesser area of healing compared to the cryogel treatment. Interestingly, the DN, DNG, and DNGM reflected a certain degree of wound healing on day 21. By contrast, the DNGM group showed superior wound closure ability, and NIR stimulation through DNGM significantly healed the wound at day 21. The statistical analysis of wound closure rate is given in Fig. 6(h). After observing the time-dependent wound area and statistical analysis, we concluded that the DNGM + NIR group had the greatest wound healing effect on mice subcutaneous wound model owing to the presence of antioxidative polymers, MXene@ZIF8 nanostructure, and light stimulation [45,65,72,78]. The wound closure rates for the control, DN, DNG, DNGM, and DNGM + NIR groups were calculated to be 60.55 ± 3.1 %, 78.64 ± 3.8 %, 79.11 ± 2.95 %, 83.62 ± 1.18 %, and 94.85 ± 2.47 %, respectively. To inspect the microscopic wound healing and inflammation, we performed histological stainings and immunocytochemistry of the wound tissue on day 14. The hematoxylin and eosin (H&E) staining results indicate the native skin-like regenerated tissue in DNGM and DNGM + NIR groups than control and other treatment groups (Fig. 6(i)). The control group displayed a lesser amount of epidermis formation after 14 days. Strikingly, the formation of the thick epidermis and granulation tissue was observed with no visible inflammatory or foreign body giant (FBG) cells at or near the wound area, suggesting the bioactive and regenerative role of DNGM cryogel under photobiomodulation. We then examined the potential of the fabricated cryogel on matrix deposition and collagen formation by Massion's trichrome (MT) staining. The MT staining showed the presence of collagen fibers (deep blue), muscle fibers (red), and blood cells (light red), as illustrated in Fig. 6(j). The control groups showed loose and disorganized collagen bundles scattered through the dermis region, which could probably be due to bleeding or infection. Notably, the cryogel-treated wound tissues displayed more ordered and well-organized collagen fibers with deep blue staining, followed by activated myofibroblasts and neoangiogenesis at day 14. The magnified images of the MT staining at the scaffold-wound interface are given in Fig. S37. The DNGM + NIR group additionally exhibited thick bundles of collagen fibers with ordered structures, whereas the DN and control group exhibited dense and disorderly arranged collagen fibers. It has been known that excessive and irregular collagen deposition in the wound bed may cause hypertrophic scar formation owing to the activity of pro-inflammatory macrophages, infection, and ECM mechanical forces, leading to improper skin regeneration [4,79]. In this context, the excessive collagen deposition, as characterized by the dark blue color in the DN group, suggests an induction of hypertrophic scarring. Meanwhile, the DNG, DNGM, and DNGM + NIR groups exhibited less scarring and proper collagen deposition, closely mimicking the native skin. The scar tissue thickness and fibrosis index for control, DN, DNG, DNGM, and DNGM + NIR groups are shown in Fig. S38. The statistical analysis of microscopic wound healing suggests that DNGM + NIR group mice had significantly less immature tissue with a thicker epidermis than the control and other groups (Fig. 6(m and n)). Moreover, the granulation tissue width, newly formed blood vessels, and hair follicles were found to be significantly higher with greater collagen volume fraction in the DNGM + NIR group than the control at day 14 (Fig. 6(o–r)), deciphering its superior wound healing potential. The DNGM + NIR groups exhibited significantly (∗∗∗∗*p* < 0.0001) higher amount of microvessel (CD105 expression) at day 14 than control (Fig. S39(a and b)), suggesting its potential for inducing neoangiogenesis.

Inflammation plays a pivotal role in chronic wound healing, and anti-inflammation regulation through immunomodulatory hydrogels is of great clinical interest. Moreover, 2D nanomaterials, such as MXene and its nanocomposites, have gained enormous attention in biomedical engineering owing to their ROS attenuating, robust antibacterial, and anti-inflammatory properties, which could be beneficial for treating infected wounds [80]. Furthermore, it has been shown that light stimulation can also ameliorate tissue healing by activating the M2 polarization of macrophages [69]. Therefore, to investigate the level of inflammation, we stain the wound beds using M1 (iNOS) and M2-polarization (CD163) specific markers at day 14. As expected, the control group exhibited higher expression of iNOS^+^ cells with significantly (∗∗*p* < 0.01) low expression of CD163^+^ cells, suggesting acute inflammatory response due to lack of biomaterials (Fig. 6(k)). Strikingly, the DNGM + NIR group showed less fluorescence for iNOS and a significantly (∗∗∗*p* < 0.001) higher expression of CD163 (Fig. 6(l)), suggesting the activation of pro-healing macrophages at or near the wound bed. This was also verified by performing qRT-PCR of the inflammation-related gene markers (*iNOS*, *TNF-α*, *CD163*, and *IL-10*) expression at day 14 and day 21, and the results are shown in Fig. S40. The significantly higher expression of *CD163* (>10.0 fold, ∗∗∗∗*p* < 0.0001) and *IL-10* (>20.0 fold, ∗∗∗∗*p* < 0.0001) markers with a low *TNF*-*α* (<2.0 fold, ∗∗∗*p* < 0.001) expression confirmed the anti-inflammatory activation of macrophages. Taken together, these findings envisioned the promising role of DNGM cryogel in attenuating inflammation and tissue repair by photobiomodulation.

### 2.8 Mechanisms of cryogel-guided wound healing

**Fig. 7:**
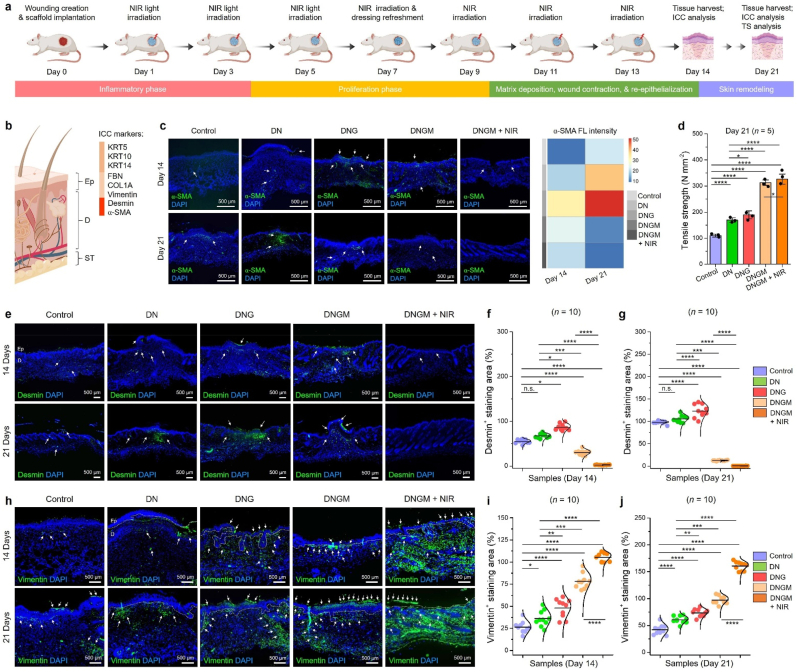
Immunostaining results of myofibroblasts and fibroblast-specific biomarkers after cryogel implantation *in vivo*. **(a)** Schematic illustration of the experimental design and wound remodeling events adopted in this study. **(b)** Schematic illustration of the skin wound-related biomarkers used in this study. Epidermal (KRT5, KRT10, and KRT14), dermal (FBN, COL1A, and Vimentin), myofibroblast (α-SMA and Desmin), and growth factor (EGFR) signaling markers are used after 14 and 21 days of cryogel implantation *in vivo*. **(c)** Immunostaining images with statistical analysis of α -SMA in wound bed after 14 and 21 days of treatment (*n* = 3 each). Scale bar: 500 μm. **(d)** Skin wound tensile strength (N mm^−2^) measured at day 21 in various groups (*n* = 5 each). **(e)** Immunostaining images of the Desmin in wound bed after 14 and 21 days of cryogel implantation. Scale bar: 500 μm. **(f, g)** Statistical analysis of the Desmin expression at indicated time points (*n* = 10 each). **(h)** Immunostaining images of the vimentin in wound bed after 14 and 21 days of cryogel implantation. Scale bar: 500 μm. **(i, j)** Statistical analysis of the vimentin expression at indicated time points (*n* = 10 each). Data reported as mean ± s.d. of replicated experiments, statistical significance was considered at ∗*p* < 0.05, ∗∗*p* < 0.01, ∗∗∗*p* < 0.001, and ∗∗∗∗*p* < 0.0001 (One-way ANOVA followed by Tukey's HSD *post-hoc* test).

To explore the mechanisms of DNGM cryogel and photobiomodulation-assisted wound healing in mice subcutaneous wound model, the skin cells’ proliferation, myofibroblast activity, and keratinization potential were investigated using cytochemistry and qRT-PCR analysis after 14- and 21-days post-implantation. Moreover, the regulatory potential of Zn^2+^ release in skin re-epithelialization and angiogenesis was also assessed by investigating the expression profile of matrix metalloproteinase-9 (Mmp9) and tissue inhibitor of metalloproteinase-1 (Timp1), respectively. Fig. 7(a, b) schematically shows the wound healing events and experimental details adopted in this study. The pre-clinical reports highlighted that photobiomodulation (*e*.*g*., visible and NIR) through therapeutic hydrogel ameliorates wound healing via regulating cell proliferation, ECM deposition, and alleviating inflammation [81]. Therefore, we hypothesize that the controlled delivery of Zn^2+^ from MXene@ZIF8 containing hydrogel would promote robust and scarless wound healing under mild NIR stimulation.

As expected, the control group displayed less staining intensity for α-smooth muscle actinin (α-SMA) near the dermis region on day 14, indicating no obvious myofibroblast community. Notably, the density of the myofibroblast community was found to increase in control group at day 21. Also, the DN, DNG, and DNGM groups indicated a slight amount of α-SMA fluorescence at day 7, suggesting the accumulation of myofibroblast. Interestingly, at day 21, except DNGM and DNGM + NIR groups, the control, DN, and DNG groups displayed traces of myofibroblast, which was characterized by an intense green color for α-SMA (Fig. 7(c)). The statistical analysis shows that the α-SMA^+^ staining area was significantly (∗∗*p* < 0.01) reduced in the DNGM and DNGM + NIR groups than in other groups, suggesting a complete healing without any scarring. This was also reflected in the change in mechanical strength of the wound tissue at day 21. As depicted in Fig. 7(d), the tensile strength of the control group was calculated to be 109.83 ± 6.49 N mm^−2^. Notably, a remarkable increase in skin tensile strength was observed in DNGM (312.55 ± 10.62 N mm^−2^, ∗∗∗∗*p* < 0.0001) and DNGM + NIR (325.94 ± 20.29 N mm^−2^, ∗∗∗∗*p* < 0.0001) groups than DN (170.34 ± 8.46 N mm^−2^) and DNG (189.33 ± 15.46 N mm^−2^) groups suggesting the positive role of DNGM in regulating skin elasticity and wound healing.

Similarly, the fluorescence of desmin (Des) was found to be lower in the DNGM + NIR group compared to the other groups (Fig. 7(e)). On day 14, the Des was found to express mostly in the dermis region in all the groups. However, at day 21, more Des^+^ cells were observed in the dermis and the wound area for DN and DNG with a significantly lower Des^+^ cells for DNGM + NIR group, further disclosing greater wound contraction after photobiomodulation. The statistical analysis shows that the Des^+^ staining area was significantly lower in the DNGM + NIR group (∗∗∗∗*p* < 0.0001) at day 14 with significantly less expression found at day 21 for DNGM (∗∗∗∗*p* < 0.0001) and DNGM + NIR (∗∗∗∗*p* < 0.0001) than control, DN, and DNG groups, respectively (Fig. 7(f and g)). Studies have shown that vimentin plays a significant role in coordinating various activities during wound healing. In particular, during skin injury, vimentin is expressed in the dermis and secreted from fibroblast cells, which initiates the Tgf-β1/Slug signaling pathway and helps in the transdifferentiation of keratinocytes, followed by collagen matrix formation and wound healing [82]. To explore whether the DNGM cryogel regulates the epithelial-mesenchymal transition (EMT), we performed immunostaining of the wound bed against vimentin, and the results are shown in Fig. 7(h). Surprisingly, after 14- and 21 days post-implantation, all the groups were shown to have vimentin^+^ cells in the dermis region with significantly higher staining for DNGM and DNGM + NIR groups than the control. In particular, the vimentin^+^ cells were found in both the dermis and epidermis region in the DNGM + NIR group. The quantification analysis underscores the significantly (∗∗∗∗*p* < 0.0001) higher staining area for both DNGM and DNGM + NIR groups (Fig. 7(i and j)), conferring keratinocytes’ transdifferentiation and EMT during skin remodeling.

**Fig. 8:**
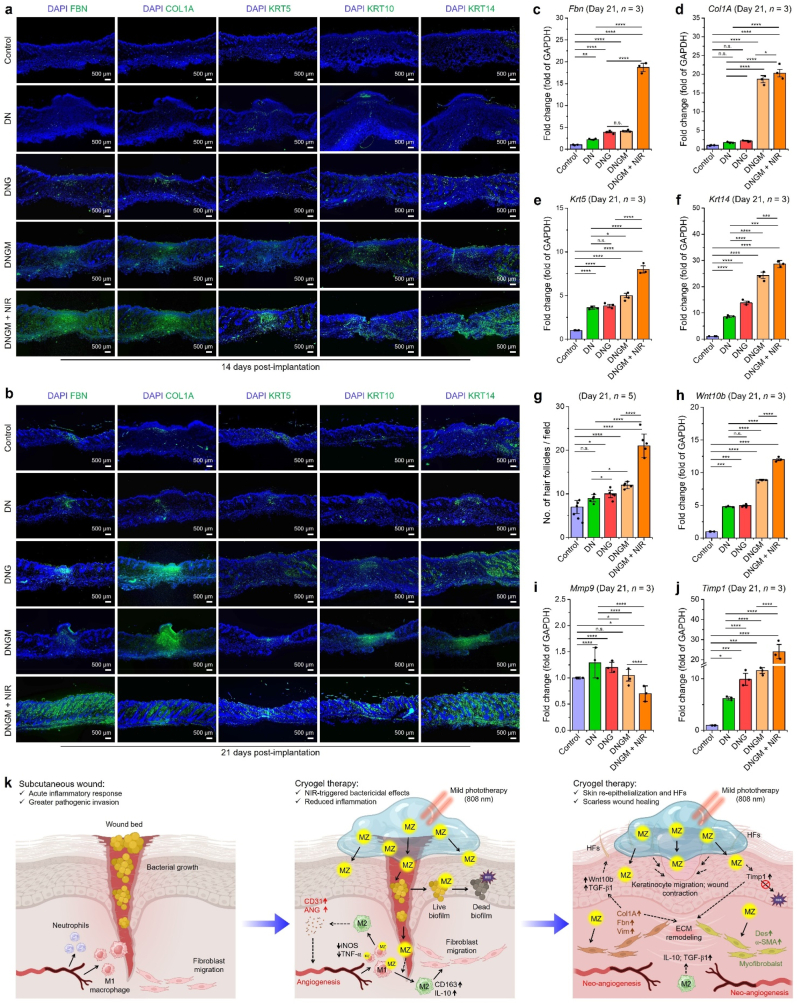
The mechanistic approach of DNGM cryogel-assisted wound healing. **(a, b)** Immunostaining results of the various wound healing markers (FBN, COL1A, KRT5, KRT10, and KRT14) after 14 and 21 days of cryogel implantation *in vivo*. Scale bar: 500 μm. **(c**–**f)** qRT-PCR analysis of the epidermal (*Krt5* and *Krt14*) and dermal (*Fbn* and *Col1A*) wound healing markers expression at day 21 (*n* = 3 each). **(g)** Statistical analysis of hair follicle (HF) number in various groups at day 21 (*n* = 5 each). **(h)** qRT-PCR analysis of *Wnt10b* gene marker at day 21 (*n* = 3 each). **(i, j)** qRT-PCR results showing the expression of *Mmp9* and *Timp1* gene markers at day 21 (*n* = 3 each). **(k)** Schematic illustration of the mechanisms of infection-free and scarless wound healing programmed by DNGM under photobiomodulation. Data reported as mean ± s.d. of replicated experiments, statistical significance was considered at ∗*p* < 0.05, ∗∗*p* < 0.01, ∗∗∗*p* < 0.001, and ∗∗∗∗*p* < 0.0001 (One-way ANOVA followed by Tukey's HSD *post-hoc* test).

Wnt/β-catenin plays a significant role wound healing and hair follicle (HF) development [83]. Studies have shown that during wound remodeling, Wnt10b is highly expressed in the hair follicle stem cells (HFSCs) through the Wnt/It is well known that cytokeratin, including Krt5 and Krt10, play a crucial role in wound healing by activating the proliferation and migration of keratinocytes during the early stage of the process. In contrast, Krt14 is putatively expressed in the epidermis during the later phase of wound healing, primarily activated through Tgf-β1 signaling pathways [84] Similarly, fibronectin (Fbn), a type of ECM protein that is highly expressed in dermal fibroblasts during chronic injury, restores the dermis by inducing hemostasis, facilitating cell migration, promoting growth factor secretion (*e*.*g*., Fgf, Vegf), encouraging keratinocyte proliferation, inducing myofibroblast differentiation, and recruiting immune cells [85] To explore this, we studied the expression profiles of Fbn, Col1A, Krt5, Krt10, and Krt14 using immunostaining and qRT-PCR at days 14 and 21. As shown in Fig. 8(a and b), all the biomarkers were highly expressed in the DNGM + NIR group, suggesting that DNGM cryogel has the potential to modulate skin re-epithelialization and ECM remodeling. In contrast, the expression of these biomarkers was lowest in the control and other groups. Notably, in the DNGM + NIR group, Col1A was highly expressed throughout the wound bed on day 14, while its expression decreased on day 21, indicating that DNGM cryogel under NIR light stimulation regulates ECM deposition and helps prevent scarring. The qRT-PCR results positively correlate with the immunostaining data. The expression of *Fbn* (>15.0 fold) and *Col1A* (>20.0 fold) gene markers was significantly higher (∗∗∗∗*p* < 0.0001) in the DNGM + NIR group compared to the others on day 21 (Fig. 8(c and d)). Similarly, the highest expressions of *Krt5* (>5.0 fold, ∗∗∗∗*p* < 0.0001) and *Krt14* (>20.0 fold, ∗∗∗∗*p* < 0.0001) were observed in the DNGM + NIR group compared to the remaining four groups (Fig. 8(e and f)), indicating skin re-epithelialization and robust wound healing.

Wnt/β-catenin plays a significant role in β-catenin signaling pathway and indirectly by keratinocytes, facilitating robust HF generation at the later stage of wound healing [75,86]. To explore whether the DNGM cryogel can induce HF development, we studied hair neogenesis by counting the hair follicles and gene expression of Wnt10b at day 21. As expected, the DNGM induced the HF formation with a significantly (∗∗∗∗*p* < 0.0001) higher number after NIR stimulation at day 21 (Fig. 8(g)), suggesting that mechanotransduction through DNGM cryogel up-regulated the Wnt/β-catenin signaling pathway in HFSCs. In contrast, the control, DN, and DNG groups displayed a lesser amount of HF development on day 21. To validate this, we then checked the mRNA expression profile of the *Wnt10b* gene, which was previously shown to up-regulate in bulk RNA-Seq. Surprisingly, the DNGM + NIR group showed a significantly higher expression of Wnt10b (>10.0 fold, ∗∗∗∗*p* < 0.0001) gene marker than others (Fig. 8(h)), concurrently supporting the RNA-Seq data and the underlying cause of the HF induction through DNGM cryogel and photobiomodulation.

The excess ECM deposition often leads to fibrosis, which can be caused by the hypoactivity of various metalloproteinases, such as Mmp9. It has been shown that Zn^2+^ activates the proteolytic function and subsequent expression of Mmp9 to cleave excess ECM formation in the wound area, thereby controlling the matrix remodeling [87]. Timp1, a tissue inhibitor of Mmp1, is also highly expressed in the later stage of wound healing to attenuate inflammatory pain and ROS burden in skin cells [88]. As stated previously, DNGM cryogel accelerates scarless wound healing; therefore, it is essential to find out the activity of Mmp9/Timp1 during biomaterial-assisted wound healing. As depicted in Fig. 8(i and j), the gene expression of *Mmp9* was found to be significantly decreased as we moved from DN to DNGM + NIR group, while the expression of *Timp1* was found to be up-regulated in DNGM + NIR group than others at day 21 which would probably be due to the release of Zn^2+^ ions from the DNGM, suggesting that DNGM + NIR group is best for promoting scarless wound healing among all samples. These results collectively indicate the promising role of DNGM cryogel in eradicating pathogenic infection and maximizing wound healing during chronic injury via inducing ECM formation, anti-inflammation, keratinocyte migration, neoangiogenesis, and HF induction (Fig. 8(k)).

## 3 Conclusion

In summary, we presented an innovative strategy to develop a programmable phototherapeutic DNA cryogel integrating plasmonic MXene@ZIF8 as a photosensitizer for wound healing through bacterial eradication, reduced inflammation, enhanced keratinocyte activity, and tissue regeneration, presenting a multifunctional, biocompatible dressing suitable for advanced wound care in clinical settings. Inspired by nature, the unique morphology of the DNGM cryogel mimics the native skin epidermis and exhibits superior bactericidal performance against pathogenic bacteria under mild phototherapy while reducing ROS burden and promoting skin regeneration. Moreover, the incorporation of MXene@ZIF8 enhanced the mechanical and viscoelastic performance of the DNA cryogel. The *in vitro* and *in vivo* results suggest the superior bioactivity of the DNGM cryogel as it showed excellent cell infiltration and keratinization and displayed robust anti-inflammatory (pro-healing) properties, setting a solid foundation for scarless wound healing. The transcriptomic study further discloses that the release of Zn^2+^ from the MXene@ZIF8 under photobiomodulation (1.0 W cm^−2^, 10 min, 808 nm wavelength) augments the up-regulation of mechanotransduction and cellular zinc metabolic events, conferring skin re-epithelialization, keratinocyte differentiation, ECM remodeling, and elevated neoangiogenesis. *In vivo* examination in an MRSA-infected wound model showed remarkable improvement in bactericidal activity, granulation tissue, neoangiogenesis, and HF development, underscoring the superior therapeutic efficacy of the DNGM cryogel under photobiomodulation. Altogether, the present study provides an innovative strategy for tissue healing and regeneration, which could be beneficial for treating infected skin wounds in clinical settings.

## 4 Experimental section

Details of the material synthesis, cryogel fabrication, characterization, *in vitro*, and *in vivo* studies are available in the Supporting **Information** section.

## CRediT authorship contribution statement

**Sayan Deb Dutta:** Writing – review & editing, Writing – original draft, Visualization, Software, Project administration, Methodology, Formal analysis, Data curation, Conceptualization. **Jeong Man An:** Writing – original draft, Methodology, Formal analysis, Data curation. **Md Moniruzzaman:** Writing – review & editing, Writing – original draft, Methodology. **Rumi Acharya:** Software, Formal analysis, Data curation. **Youjin Seol:** Software, Formal analysis, Data curation. **Hojin Kim:** Software, Formal analysis, Data curation. **Aayushi Randhawa:** Software, Formal analysis, Data curation. **Jong-Sung Kim:** Writing – review & editing, Supervision, Project administration, Funding acquisition. **Yong-kyu Lee:** Writing – review & editing, Supervision, Project administration, Funding acquisition. **Ki-Taek Lim:** Writing – review & editing, Supervision, Project administration, Investigation, Funding acquisition.

## Data availability statement

The raw data required to support these findings can be obtained from the corresponding author upon reasonable request.

## Ethics approval and consent to participate

The animal experiment was approved by the Institute of Animal Care and Use Committee (IACUC) of the Korea National University of Transportation (KNUT-2024-A7), Republic of Korea.

## Declaration of competing interest

The authors declare that they have no known competing financial interests or personal relationships that could have appeared to influence the work reported in this paper.

## References

[1] Pranantyo D., Yeo C.K., Wu Y., Fan C., Xu X., Yip Y.S., Vos M.I.G., Mahadevegowda S.H., Lim P.L.K., Yang L. (2024). Hydrogel dressings with intrinsic antibiofilm and antioxidative dual functionalities accelerate infected diabetic wound healing. Nat. Commun..

[2] Liu H., Wei X., Peng H., Yang Y., Hu Z., Rao Y., Wang Z., Dou J., Huang X., Hu Q. (2024). LysSYL‐loaded pH‐switchable self‐assembling peptide hydrogels promote methicillin‐resistant staphylococcus aureus elimination and wound healing. Adv. Mater..

[3] Wang C., Shirzaei Sani E., Shih C.-D., Lim C.T., Wang J., Armstrong D.G., Gao W. (2024). Wound management materials and technologies from bench to bedside and beyond. Nat. Rev. Mater..

[4] Dutta S.D., An J.M., Hexiu J., Randhawa A., Ganguly K., Patil T.V., Thambi T., Kim J., Lee Y.-k., Lim K.-T. (2025). 3D bioprinting of engineered exosomes secreted from M2-polarized macrophages through immunomodulatory biomaterial promotes in vivo wound healing and angiogenesis. Bioact. Mater..

[5] Ter Steeg L., Domínguez-Andrés J., Netea M.G., Joosten L.A., van Crevel R. (2021). Trained immunity as a preventive measure for surgical site infections. Clin. Microbiol. Rev..

[6] Eckert R. (2011). Road to clinical efficacy: challenges and novel strategies for antimicrobial peptide development. Future Microbiol..

[7] Eming S.A., Koch M., Krieger A., Brachvogel B., Kreft S., Bruckner-Tuderman L., Krieg T., Shannon J.D., Fox J.W. (2010). Differential proteomic analysis distinguishes tissue repair biomarker signatures in wound exudates obtained from normal healing and chronic wounds. J. Proteome Res..

[8] Liu Y., Yang X., Wu K., Feng J., Zhang X., Li A., Cheng C., Zhu Y.Z., Guo H., Wang X. (2025). Skin‐inspired and self‐regulated hydrophobic hydrogel for diabetic wound therapy. Adv. Mater..

[9] Fan R., Zhao J., Yi L., Yuan J., McCarthy A., Li B., Yang G., John J.V., Wan W., Zhang Y. (2024). Anti‐inflammatory peptide‐conjugated silk fibroin/cryogel hybrid dual fiber scaffold with hierarchical structure promotes healing of chronic wounds. Adv. Mater..

[10] Lan T., Dong Y., Shi J., Wang X., Xu Z., Zhang Y., Jiang L., Zhou W., Sui X. (2024). Advancing self‐healing soy protein hydrogel with dynamic Schiff base and metal‐ligand bonds for diabetic chronic wound recovery. Aggregate.

[11] Lachance‐Brais C., Rammal M., Asohan J., Katolik A., Luo X., Saliba D., Jonderian A., Damha M.J., Harrington M.J., Sleiman H.F. (2023). Small molecule‐templated DNA hydrogel with record stiffness integrates and releases DNA nanostructures and gene silencing nucleic acids. Adv. Sci..

[12] Li D., Tang G., Yao H., Zhu Y., Shi C., Fu Q., Yang F., Wang X. (2022). Formulation of pH-responsive PEGylated nanoparticles with high drug loading capacity and programmable drug release for enhanced antibacterial activity. Bioact. Mater..

[13] Li X., Chen X., Guan L., He W., Yin W., Ye D., Gao J., Wang M., Pan G. (2024). Bioactive metal ion-coordinated dynamic hydrogel with antibacterial, immunomodulatory, and angiogenic activities for infected wound repair. ACS Appl. Mater. Interfaces.

[14] Han X., Saengow C., Ju L., Ren W., Ewoldt R.H., Irudayaraj J. (2024). Exosome-coated oxygen nanobubble-laden hydrogel augments intracellular delivery of exosomes for enhanced wound healing. Nat. Commun..

[15] Xiong M., Yang X., Shi Z., Xiang J., Gao H., Ji S., Li Y., Pi W., Chen H., Zhang H. (2024). Programmable artificial skins accomplish antiscar healing with multiple appendage regeneration. Adv. Mater..

[16] Liu Y., Ma Q., Tang L., Shen Y., Zhao H., Liu X., Lin D., Zhou G. (2024). A multifunctional hydrogel with mild photothermal antibacterial and antioxidant properties based on quercetin and dopamine-coated zinc oxide nanoparticles for healing bacteria-infected wound. Chem. Eng. J..

[17] Wang Z., Luo H., Wang H., Xiao M., Jia H., Ren C., Liu J. (2024). Peptide‐based supramolecular therapeutics for fighting major diseases. Adv. Funct. Mater..

[18] Zhao F., Su Y., Wang J., Romanova S., DiMaio D.J., Xie J., Zhao S. (2023). A highly efficacious electrical biofilm treatment system for combating chronic wound bacterial infections. Adv. Mater..

[19] Yang Y., Fang Q., Wang J., Li M., Li Z., Xu H., Huang S., Chen J., Guo B. (2025). Glucose‐activated programmed hydrogel with self‐switchable enzyme‐like activity for infected diabetic wound self‐adaptive treatment. Adv. Mater..

[20] He L., Di D., Chu X., Liu X., Wang Z., Lu J., Wang S., Zhao Q. (2023). Photothermal antibacterial materials to promote wound healing. J. Contr. Release.

[21] Hu Y., Wang F., Ye H., Jiang J., Li S., Dai B., Li J., Yang J., Song X., Zhang J. (2024). MXene-based flexible electronic materials for wound infection detection and treatment. npj Flex Electron..

[22] Xu D., Li Z., Li L., Wang J. (2020). Insights into the photothermal conversion of 2D MXene nanomaterials: synthesis, mechanism, and applications. Adv. Funct. Mater..

[23] Yang X., Zhang C., Deng D., Gu Y., Wang H., Zhong Q. (2022). Multiple stimuli‐responsive MXene‐based hydrogel as intelligent drug delivery carriers for deep chronic wound healing. Small.

[24] Li M., Zhang Y., Lian L., Liu K., Lu M., Chen Y., Zhang L., Zhang X., Wan P. (2022). Flexible accelerated‐wound‐healing antibacterial MXene‐based epidermic sensor for intelligent wearable human‐machine interaction. Adv. Funct. Mater..

[25] Huang F., Chen M., Zhou Z., Duan R., Xia F., Willner I. (2021). Spatiotemporal patterning of photoresponsive DNA-based hydrogels to tune local cell responses. Nat. Commun..

[26] Dutta S.D., Ganguly K., Randhawa A., Patil T., Patel D.K., Lim K.-T. (2023). Electrically stimulated 3D bioprinting of gelatin-polypyrrole hydrogel with dynamic semi-IPN network induces osteogenesis via collective signaling and immunopolarization. Biomaterials.

[27] Lim K.R.G., Shekhirev M., Wyatt B.C., Anasori B., Gogotsi Y., Seh Z.W. (2022). Fundamentals of MXene synthesis. Nat. Synth..

[28] Jiang Y., Sun T., Xie X., Jiang W., Li J., Tian B., Su C. (2019). Oxygen‐functionalized ultrathin Ti3C2Tx MXene for enhanced electrocatalytic hydrogen evolution. ChemSusChem.

[29] Cheng F., Yi X., Dai J., Fan Z., He J., Huang Y., Li H. (2023). Photothermal MXene@ Zn-MOF-decorated bacterial cellulose-based hydrogel wound dressing for infectious wound healing. Cell Rep. Phys. Sci..

[30] Ranjith K.S., Sonwal S., Mohammadi A., Raju G.S.R., Oh M.-H., Huh Y.S., Han Y.-K. (2024). Imparting hydrophobicity to a MOF on layered MXene for the selective, rapid, and ppb level humidity-independent detection of NH 3 at room temperature. J. Mater. Chem. A.

[31] Raj S.M.M., Sundramoorthy A.K., Atchudan R., Ganapathy D., Khosla A. (2022). recent trends on the synthesis and different characterization tools for MXenes and their emerging applications. J. Electrochem. Soc..

[32] Magesh V., Sundramoorthy A.K., Ganapathy D., Atchudan R., Arya S., Alshgari R.A., Aljuwayid A.M. (2022). Palladium hydroxide (Pearlman’s catalyst) doped MXene (Ti3C2Tx) composite modified electrode for selective detection of nicotine in human sweat. Biosensors.

[33] Zhang J., Usman K.A.S., Judicpa M.A.N., Hegh D., Lynch P.A., Razal J.M. (2023). Applications of X‐ray‐based characterization in MXene research. Small Methods.

[34] Park K.S., Ni Z., Côté A.P., Choi J.Y., Huang R., Uribe-Romo F.J., Chae H.K., O'Keeffe M., Yaghi O.M. (2006). Exceptional chemical and thermal stability of zeolitic imidazolate frameworks. Proc. Natl. Acad. Sci..

[35] Dixit P., Maiti T. (2022). A facile pot synthesis of (Ti3AlC2) MAX phase and its derived MXene (Ti3C2Tx). Ceram. Int..

[36] Abdullah M., Elango I., Patil H., Patil P.P., Aloysius D., Gupta S., Selvamani M., Kim D.-k., Dongale T.D., Kesavan A.V. (2024). Metal-organic framework and MXene (ZIF-8: Ti3C2Tx) based organic and inorganic nanocomposite for bio-synaptic applications. Surf. Interfaces.

[37] Sarycheva A., Gogotsi Y. (2023). Raman Spectroscopy Analysis of the Structure and Surface Chemistry of Ti3C2Tx MXene, MXenes.

[38] Natu V., Benchakar M., Canaff C., Habrioux A., Célérier S., Barsoum M. (2021). A critical analysis of the X-ray photoelectron spectra of Ti3C2Tz MXenes. Matter.

[39] Halim J., Cook K.M., Naguib M., Eklund P., Gogotsi Y., Rosen J., Barsoum M.W. (2016). X-ray photoelectron spectroscopy of select multi-layered transition metal carbides (MXenes). Appl. Surf. Sci..

[40] Wang L., Song L., Yang Z., Chang Y.M., Hu F., Li L., Li L., Chen H.Y., Peng S. (2023). Electronic modulation of metal–organic frameworks by interfacial bridging for efficient pH‐universal hydrogen evolution. Adv. Funct. Mater..

[41] Jin L., Guo X., Gao D., Wu C., Hu B., Tan G., Du N., Cai X., Yang Z., Zhang X. (2021). NIR-responsive MXene nanobelts for wound healing. NPG Asia Mater..

[42] Yang W., Sherman V.R., Gludovatz B., Schaible E., Stewart P., Ritchie R.O., Meyers M.A. (2015). On the tear resistance of skin. Nat. Commun..

[43] Li J., Liu X., Tan L., Cui Z., Yang X., Liang Y., Li Z., Zhu S., Zheng Y., Yeung K.W.K. (2019). Zinc-doped Prussian blue enhances photothermal clearance of *Staphylococcus aureus* and promotes tissue repair in infected wounds. Nat. Commun..

[44] S.E. Microscopy, Visualizing Skin Tissue Morphology with Scanning Electron Microscopy, nanoScience Instruments.

[45] Ye R., Zhu Z., Gu T., Cao D., Jiang K., Dai Q., Xing K., Jiang Y., Zhou S., Cai P. (2024). Neutrophil extracellular traps-inspired DNA hydrogel for wound hemostatic adjuvant. Nat. Commun..

[46] Gao F., Ma X., Wang F., Zhou F., Ye J., Yang D., Li M., Wang P. (2023). Injectable multifunctional DNA hydrogel for accelerated wound healing. Chem. Eng. J..

[47] Dutta S.D., Ganguly K., Randhawa A., Patil T.V., Patel D.K., Lim K.-T. (2023). Electrically stimulated 3D bioprinting of gelatin-polypyrrole hydrogel with dynamic semi-IPN network induces osteogenesis via collective signaling and immunopolarization. Biomaterials.

[48] Rolim T., Cancino J., Zucolotto V. (2015). A nanostructured genosensor for the early diagnosis of systemic arterial hypertension. Biomed. Microdevices.

[49] Hayashi K., Okamoto F., Hoshi S., Katashima T., Zujur D.C., Li X., Shibayama M., Gilbert E.P., Chung U.-i., Ohba S. (2017). Fast-forming hydrogel with ultralow polymeric content as an artificial vitreous body. Nat. Biomed. Eng..

[50] Wang D., Duan J., Liu J., Yi H., Zhang Z., Song H., Li Y., Zhang K. (2023). Stimuli‐responsive self‐degradable DNA hydrogels: design, synthesis, and applications. Adv. Healthcare Mater..

[51] Athanasiadou D., Meshry N., Monteiro N.G., Ervolino-Silva A.C., Chan R.L., McCulloch C.A., Okamoto R., Carneiro K.M. (2023). DNA hydrogels for bone regeneration. Proc. Natl. Acad. Sci..

[52] Hirsch L.R., Stafford R.J., Bankson J., Sershen S.R., Rivera B., Price R., Hazle J.D., Halas N.J., West J.L. (2003). Nanoshell-mediated near-infrared thermal therapy of tumors under magnetic resonance guidance. Proc. Natl. Acad. Sci..

[53] Raghupathi K.R., Koodali R.T., Manna A.C. (2011). Size-dependent bacterial growth inhibition and mechanism of antibacterial activity of zinc oxide nanoparticles. Langmuir.

[54] Li Y., Chen W., Yin J., Xia S., Jiang Y., Ge Q., Liu J., Wang M., Hou Z., Bai Y. (2024). Biomineralized ZIF‐8 encapsulating SOD from hydrogenobacter thermophilus: maintaining activity in the intestine and alleviating intestinal oxidative stress. Small.

[55] Mendes C.R., Dilarri G., Forsan C.F., Sapata V.d.M.R., Lopes P.R.M., de Moraes P.B., Montagnolli R.N., Ferreira H., Bidoia E.D. (2022). Antibacterial action and target mechanisms of zinc oxide nanoparticles against bacterial pathogens. Sci. Rep..

[56] Seidi F., Arabi Shamsabadi A., Dadashi Firouzjaei M., Elliott M., Saeb M.R., Huang Y., Li C., Xiao H., Anasori B. (2023). MXenes antibacterial properties and applications: a review and perspective. Small.

[57] Taheri M., Ashok D., Sen T., Enge T.G., Verma N.K., Tricoli A., Lowe A., Nisbet D.R., Tsuzuki T. (2021). Stability of ZIF-8 nanopowders in bacterial culture media and its implication for antibacterial properties. Chem. Eng. J..

[58] Uberoi A., McCready-Vangi A., Grice E.A. (2024). The wound microbiota: microbial mechanisms of impaired wound healing and infection. Nat. Rev. Microbiol..

[59] Yang Y., Ma L., Cheng C., Deng Y., Huang J., Fan X., Nie C., Zhao W., Zhao C. (2018). Nonchemotherapic and robust dual‐responsive nanoagents with on‐demand bacterial trapping, ablation, and release for efficient wound disinfection. Adv. Funct. Mater..

[60] Peng B., Zhang X., Aarts D.G., Dullens R.P. (2018). Superparamagnetic nickel colloidal nanocrystal clusters with antibacterial activity and bacteria binding ability. Nat. Nanotechnol..

[61] Vitasovic T., Caniglia G., Eghtesadi N., Ceccato M., Bo̷jesen E.D., Gosewinkel U., Neusser G., Rupp U., Walther P., Kranz C. (2024). Antibacterial action of Zn2+ ions driven by the in vivo formed ZnO nanoparticles. ACS Appl. Mater. Interfaces.

[62] Ogawa Y., Kinoshita M., Shimada S., Kawamura T. (2018). Zinc in keratinocytes and langerhans cells: relevance to the epidermal homeostasis. J. Immunol. Res..

[63] Lee S.Y., Jeon S., Kwon Y.W., Kwon M., Kang M.S., Seong K.-Y., Park T.-E., Yang S.Y., Han D.-W., Hong S.W. (2022). Combinatorial wound healing therapy using adhesive nanofibrous membrane equipped with wearable LED patches for photobiomodulation. Sci. Adv..

[64] Wang Z., Lee W., Koh B., Hong M., Wang W., Lim P., Feng J., Park L., Kim M., Thian E. (2018). Functional regeneration of tendons using scaffolds with physical anisotropy engineered via microarchitectural manipulation. Sci. Adv..

[65] Wang Z., Li W., Gou L., Zhou Y., Peng G., Zhang J., Liu J., Li R., Ni H., Zhang W. (2022). Biodegradable and antioxidant DNA hydrogel as a cytokine delivery system for diabetic wound healing. Adv. Healthcare Mater..

[66] Villarreal-Ponce A., Tiruneh M.W., Lee J., Guerrero-Juarez C.F., Kuhn J., David J.A., Dammeyer K., Mc Kell R., Kwong J., Rabbani P.S. (2020). Keratinocyte-macrophage crosstalk by the Nrf2/Ccl2/EGF signaling axis orchestrates tissue repair. Cell Rep..

[67] Chakraborty S., Sampath D., Yu Lin M.O., Bilton M., Huang C.-K., Nai M.H., Njah K., Goy P.-A., Wang C.-C., Guccione E. (2021). Agrin-Matrix Metalloproteinase-12 axis confers a mechanically competent microenvironment in skin wound healing. Nat. Commun..

[68] Chen S., Saeed A.F., Liu Q., Jiang Q., Xu H., Xiao G.G., Rao L., Duo Y. (2023). Macrophages in immunoregulation and therapeutics. Signal Transduct. Targeted Ther..

[69] Liao W.-T., Hung C.-H., Liang S.-S., Yu S., Lu J.-H., Lee C.-H., Chai C.-Y., Yu H.-S. (2021). Anti-inflammatory effects induced by near-infrared light irradiation through M2 macrophage polarization. J. Invest. Dermatol..

[70] Jiang X., Ma J., Xue K., Chen J., Zhang Y., Zhang G., Wang K., Yao Z., Hu Q., Lin C. (2024). Highly bioactive MXene-M2-exosome nanocomposites promote angiogenic diabetic wound repair through reconstructing high glucose-derived immune inhibition. ACS Nano.

[71] Huang B., Li S., Dai S., Lu X., Wang P., Li X., Zhao Z., Wang Q., Li N., Wen J. (2024). Ti3C2Tx MXene‐decorated 3D‐printed ceramic scaffolds for enhancing osteogenesis by spatiotemporally orchestrating inflammatory and bone repair responses. Adv. Sci..

[72] Zhang Q., Jiang Y., Zhang X., Wang Y., Ju R., Wei G. (2024). Injectable and near-infrared light-controllable fibrin hydrogels with antimicrobial and immunomodulating properties for infected wound healing. Biomater. Res..

[73] Kamaliyan Z., Clarke T.L. (2024). Zinc finger proteins: Guardians of genome stability. Front. Cell Dev. Biol..

[74] Fernández-Parejo N., Lorenzo-Martín L.F., García-Pedrero J.M., Rodrigo J.P., Dosil M., Bustelo X.R. (2024). VAV2 orchestrates the interplay between regenerative proliferation and ribogenesis in both keratinocytes and oral squamous cell carcinoma. Sci. Rep..

[75] Liu J., Xiao Q., Xiao J., Niu C., Li Y., Zhang X., Zhou Z., Shu G., Yin G. (2022). Wnt/β-catenin signalling: function, biological mechanisms, and therapeutic opportunities. Signal Transduct. Targeted Ther..

[76] Sreenivasamurthy S.A., Akhter F.F., Akhter A., Su Y., Zhu D. (2022). Cellular mechanisms of biodegradable zinc and magnesium materials on promoting angiogenesis. Biomater. Adv..

[77] Luo B., Lei J., Wen R., Hu X., Liu S., Dong P., Lan F., Wu Y. (2024). MXene/metal–organic framework heterojunctions facilitate bacterial-infected wound repair via exogenous and endogenous synergistic stimulations. ACS Mater. Lett..

[78] Kim N., Lee H., Han G., Kang M., Park S., Kim D.E., Lee M., Kim M.J., Na Y., Oh S. (2023). 3D‐Printed functional hydrogel by DNA‐induced biomineralization for accelerated diabetic wound healing. Adv. Sci..

[79] Konieczny P., Naik S. (2021). Healing without scarring. Science.

[80] Ma J., Zhang L., Lei B. (2023). Multifunctional MXene-based bioactive materials for integrated regeneration therapy. ACS Nano.

[81] Dungel P., Hartinger J., Chaudary S., Slezak P., Hofmann A., Hausner T., Strassl M., Wintner E., Redl H., Mittermayr R. (2014). Low level light therapy by LED of different wavelength induces angiogenesis and improves ischemic wound healing. Laser Surg. Med..

[82] Cheng F., Shen Y., Mohanasundaram P., Lindström M., Ivaska J., Ny T., Eriksson J.E. (2016). Vimentin coordinates fibroblast proliferation and keratinocyte differentiation in wound healing via TGF-β–Slug signaling. Proc. Natl. Acad. Sci..

[83] Oak A.S., Bagchi A., Brukman M.J., Toth J., Ford J., Zheng Y., Nace A., Yang R., Hsieh J.-C., Hayden J.E. (2025). Wnt signaling modulates mechanotransduction in the epidermis to drive hair follicle regeneration. Sci. Adv..

[84] Richards S.M., Gubser Keller C., Kreutzer R., Greiner G., Ley S., Doelemeyer A., Dubost V., Flandre T., Kirkland S., Carbone W. (2024). Molecular characterization of chronic cutaneous wounds reveals subregion‐and wound type‐specific differential gene expression. Int. Wound J..

[85] Martino M.M., Tortelli F., Mochizuki M., Traub S., Ben-David D., Kuhn G.A., Müller R., Livne E., Eming S.A., Hubbell J.A. (2011). Engineering the growth factor microenvironment with fibronectin domains to promote wound and bone tissue healing. Sci. Transl. Med..

[86] Wang X., Liu Y., He J., Wang J., Chen X., Yang R. (2022). Regulation of signaling pathways in hair follicle stem cells. Burns & Trauma.

[87] Ridnour L.A., Windhausen A.N., Isenberg J.S., Yeung N., Thomas D.D., Vitek M.P., Roberts D.D., Wink D.A. (2007). Nitric oxide regulates matrix metalloproteinase-9 activity by guanylyl-cyclase-dependent and-independent pathways. Proc. Natl. Acad. Sci..

[88] Knight B., Kozlowski N., Havelin J., King T., Crocker S., Young E., Baumbauer K. (2019). TIMP-1 attenuates the development of inflammatory pain through MMP-dependent and receptor-mediated cell signaling mechanisms. Front. Mol. Neurosci..

