# Corrigendum to “Skin-inspired phototherapeutic cryogel ameliorates infected wound healing by orchestrating mechanotransduction and immunomodulation” [Bioact. Mater. 57 (2026) 768–790]

**DOI:** 10.1016/j.bioactmat.2025.12.003

**Published:** 2025-12-08

**Authors:** Sayan Deb Dutta, Jeong Man An, Md Moniruzzaman, Rumi Acharya, Youjin Seol, Hojin Kim, Aayushi Randhawa, Jong-Sung Kim, Yong-kyu Lee, Ki-Taek Lim

**Affiliations:** a Department of Biosystems Engineering, Kangwon National University, Chuncheon-si, 24341, Republic of Korea; b Institute of Forest Science, Kangwon National University, Chuncheon-si, 24341, Republic of Korea; c School of Medicine, The University of California Davis, Sacramento, 95817, United States; d Department of Bioengineering, Hanyang University, Seoul, 04763, Republic of Korea; e Department of Chemical and Biological Engineering, Gachon University, Seongnam-si, 13120, Republic of Korea; f Interdisciplinary Program in Smart Agriculture, Kangwon National University, Chuncheon-si, 24341, Republic of Korea; g Department of Chemical and Biological Engineering, Korea National University of Transportation, Chungju-si, 27470, Republic of Korea

The authors would like to change some of the funding information details due to the sudden change in funding numbers from the funding agency.

**Old Acknowledgements:**

The Basic Science Research Program supported this work through the 10.13039/501100003725National Research Foundation of Korea (Grant No. RS-2018-NR031068 and RS-2025-16065692), Republic of Korea. This work was supported by the Innovative Human Resource Development for Local Intellectualization program through the Institute of Information & Communications Technology Planning & Evaluation (IITP) grant funded by the Korean Government (IITP) (RS-2023-00260267). This research was also supported by the 10.13039/501100003725National Research Foundation of Korea (NRF) grant funded by the Korean Government (MSIT) (RS-2022-NR069546, RS-2021-NR060117, RS-2024-00405287, and 2021R1A2C2095113).

**New Acknowledgements:**

The Basic Science Research Program supported this work through the 10.13039/501100003725National Research Foundation of Korea (Grant No. RS-2018-NR031068 and RS-2025-16065692), Republic of Korea. This work was supported by the Innovative Human Resource Development for Local Intellectualization program through the Institute of Information & Communications Technology Planning & Evaluation (IITP) grant funded by the Korean Government (IITP) (RS-2023-00260267). This research was also supported by the 10.13039/501100003725National Research Foundation of Korea (NRF) grant funded by the Korean Government (MSIT) (RS-2022-NR069546, RS-2021-NR060117, RS-2021-NR060137, and 2021R1A6A1A03046418).

The authors would like to apologise for any inconvenience caused.

