# Supplementary material for: Skin-inspired phototherapeutic cryogel ameliorates infected wound healing by orchestrating mechanotransduction and immunomodulation
Source: Bioact Mater. 2025 Nov 29;57:768–90. doi: 10.1016/j.bioactmat.2025.11.028 (PMC12704289; doi:10.1016/j.bioactmat.2025.11.028)
Supplement: Multimedia component 1 [file mmc1.docx]

Supplementary Materials for

**Skin-inspired Phototherapeutic Cryogel Ameliorates Infected Wound Healing by Orchestrating Mechanotransduction and Immunomodulation**

**This SI file includes:**

Supplementary Methods (Page No. S2-S13)

Figs. S1 to S40 (Page No. S14-S53)

Tables S1 to S6 (Page No. S54-59)

Supplementary References (Page No. S60)

Supplementary Methods

**1. Synthesis of Ti_3_C_2_T_x_ nanosheets**

The Ti_3_C_2_T_x_ (MXene) was synthesized as reported elsewhere.^[^[^1^](#_ENREF_1)^]^ In a typical synthesis, 0.5 g of the MAX phase (Ti_3_AlC_2_, Sigma-Aldrich) was mixed with an etching mixture containing 1.2 g LiF (Sigma-Aldrich) and 9 M (20 mL) HCL (Sigma-Aldrich) in a glass beaker under vigorous stirring. Subsequently, the resultant solution was agitated at 37 ± 2 °C for 80 h, followed by centrifugation and washing (3-5 times) with D.I. water/ethanol to obtain a neutral pH. The as-obtained product was dried at 60 °C to obtain Ti_3_C_2_T_x_ (MXene) nanosheets.

**2. Fabrication of MXene@ZIF8 nanocomposite**

The MXene@ZIF8 nanocomposite was fabricated using the solvothermal method. In a typical process, 810 mg of zinc nitrate hexahydrate (Zn(NO_3_) ·6H_2_O, Sigma-Aldrich) and a methanolic solution of 526 mg 2-methyl imidazole (2-Melm, Sigma-Aldrich) were mixed at ambient temperature under magnetic stirring. After this, 250 mg of MXene was added to the solution and stirred for 60 minutes. Afterward, the mixture was transferred into a Teflon^®^-lined autoclave and heated to 100 °C for 12 hours. Next, the dark grayish mass was centrifuged, washed several times with DI water, and dried in a hot-air oven for 48 hours. A control ZIF8 without MXene was also synthesized by adding only Zn(NO_3_)_2_ and 2-Melm.

**3. Synthesis of highly substituted gelatin methacryloyl (GelMA)**

The GelMA with a degree of substitution (DS) of approximately 70% was synthesized as described in our previous study.^[^[^2^](#_ENREF_2)^]^ Briefly, 10 g of gelatin (Type A, Sigma-Aldrich) was dissolved in 1× PBS (Welgene, Inc.) at 45 °C with constant stirring for 1-2 hours. Next, 2 mL of methacrylic anhydride (Sigma-Aldrich) was added to the gelatin solution and stirred for 2 hours. The methacrylation reaction was then halted by adding twice the amount of warm PBS (1×), followed by dialysis using a 12-14 kDa dialysis tube (Viskase) for a minimum of 7 days to eliminate unreacted methacrylic acid. Lastly, the whitish GelMA foam was acquired by freeze-drying the dialysis product and was stored at -20 °C until needed.

**4. Fabrication of DNA cryogel containing MXene@ZIF8**

The skin epidermis-mimicking, aligned, and porous DNA cryogel with MXene@ZIF8 was fabricated using the unidirectional ice crystal freezing method. To achieve this, 6% (*w*/*v*) of deoxyribonucleic acid sodium salt derived from salmon sperm (DNA, Sigma-Aldrich) was dissolved in D.I. water at 70 °C with vigorous stirring for 2 hours. Subsequently, the temperature was lowered to 45 °C, and 0.5% (*w*/*v*) GelMA was incorporated into the DNA solution, which was stirred for an additional hour until fully dissolved. The solution was then transferred to an ice bath, followed by the addition of 100 µg mL^-1^ MXene@ZIF8, 0.5% (*w*/*v*) ammonium persulfate (APS, Sigma-Aldrich), and 0.1% (*w*/*v*) tetramethylethylenediamine (TEMED, Sigma-Aldrich), stirred for another hour. The APS and TEMED serve as radical crosslinkers during hydrogel formation. The resulting mixture was incubated for 5 min at 90 °C to anneal the DNA strands and then transferred into a silicone mold (10 × 8 mm^²^) and placed on a copper plate to facilitate unidirectional ice crystal growth. The mold was insulated with Styrofoam™ (Sigma-Aldrich) and frozen at -80 °C for 48 hours. Finally, after 48 hours, the samples were removed, washed with 1× PBS, and lyophilized to yield cryogel scaffolds. The as-prepared cryogel scaffolds were kept at 4 °C for long-term storage and sterilized by UV light in a sterile culture hood before cell culture and other *in vitro* experiments. The concentration of MXene@ZIF8 was determined by assessing the cell viability of human keratinocyte (HaCaT) cells before cryogel fabrication. The pure DNA, DNA/GelMA, and DNA/GelMA/MXene@ZIF8 cryogel scaffolds were designated as DN, DNG, and DNGM.

**5. General characterizations**

EF-SEM (JSM-7900F, Jeol) was used to examine the surface micromorphology of MXene and MXene@ZIF8. The structural and chemical composition of MXene and MXene@ZIF8 was analyzed using FT-IR (iN10/iS50, Thermo Scientific), XRD (X’Pert Pro MPD, PANalytical), and Raman spectroscopy. The near-infrared (NIR) responsive properties of MXene and MXene@ZIF8 were evaluated using UV-Vis spectroscopy (Spectramax ABS Plus, Molecular Devices) within a range of 400-1000 nm. The surface functional groups of MXene and MXene@ZIF8 were investigated using XPS (K Alpha+, Thermo VG) spectroscopy. The morphology and composition of unmodified ZIF8 were analyzed using HR-TEM (JEM-2100F, Jeol) and FT-IR spectroscopy. The morphology of the freeze-dried cryogels was examined using FE-SEM (JSM-7900F, Jeol) at a 5.0 kV accelerating voltage in gentle beam (GB) mode. The viscoelasticity of the fabricated cryogels was assessed using a rotational rheometer (MCR-92, Anton Paar) under varying frequencies (0.1-100 Rad s^-1^) at room temperature. The initial shear stress was calculated in the low-frequency region (0.1-1 Rad s^-1^) for all the cryogels. The wettability properties of the DN, DNG, and DNGM scaffolds were examined through a conventional swelling test, as reported in our previous study.^[^[^2^](#_ENREF_2)^]^ The chemical composition of the fabricated cryogel scaffolds was analyzed using FT-IR spectroscopy.

**6. *In vitro* degradation study of cryogels**

The *in vitro* degradation property of the fabricated cryogels was evaluated under two conditions: (1) the mass degradation method and (2) the degradation or release of free DNA in DMEM (without serum) media. For the mass degradation study, the cryogel scaffold (~200 mg) was placed in a glass vial and incubated with 1× PBS (10 mL) or pronase/trypsin (0.1 mg mL^-1^ each) at 37 °C with mild stirring for 7 days. At various time points, the cryogel scaffold was removed, excess PBS was blotted, and the scaffold was dried at 45 °C. The dried mass was recorded, and the degradation rate was calculated as a percentage of the residual mass. For the second method, ~50 mg of DNGM cryogel was incubated with 1 mL of DMEM (Welgene, Inc.) media at 37 °C in a sterile environment for up to 7 days. After the desired time points (0, 1, 5, and 7 days), 100 µL of supernatant was collected, and the degradation or release of DNA was quantified by denaturing PAGE. For PAGE analysis, 100 µL of supernatant was mixed with 50 µL of formamide (TCI Co., Ltd.), and the mixture was thoroughly combined by pipetting and vortexing for 30 seconds. Then, 0.5 µL of the mixture was added to 7.25 µL of formamide and 7.25 µL of nuclease-free water (total volume: 15 µL) before being loaded into each gel lane. The denaturing PAGE was conducted at 300 V and 15 mA current for 2 hours using a gel apparatus (Bio-Rad™). Finally, the gel was stained with Gel-Green^®^ nucleic acid stain (Sigma-Aldrich), and imaging was performed using ChemiDoc™ XRS+ (Bio-Rad™).

**7. Photothermal performances**

The photothermal conversion efficiency of the MXene and MXene@ZIF8 (100 µg ml^-1^ each) was measured as reported in our previous study.^[^[^3^](#_ENREF_3)^]^ For the photothermal measurements, approximately 50 mg of each cryogel sample (DN, DNG, and DNGM) was placed in a 48-well plate and irradiated for 10 minutes using an 808 nm laser source (MSL-Ti, 500 mW, CNI Lasers) at various power densities (0.5, 1.0, and 1.5 W cm^-2^). The temperature change was monitored using a thermal camera (E6390, FLIR). D.W. was used as a control sample for all the measurements. The DNGM cryogel was irradiated with 808 nm NIR light for five minutes to assess photothermal stability, followed by subsequent cooling cycles. Each measurement was replicated three times (*n* = 3) over time.

To gain insights into the origin of photothermal efficiency, the energy level of the frontier orbitals of MXene and MXene@ZIF8 was studied using cyclic voltammetry. For this, an aqueous concentration of MXene and MXene@ZIF8 (100 µg ml^-1^ each) was drop-casted onto the surface of a glassy carbon electrode (GCE, working electrode) and the electrochemical oxidation-reduction (E*_ox_* or E*_red_*) potential of the samples was analyzed using three electrode system with 0.1 M H_2_SO_4_ as an electrolyte and the experiment was conducted at a scan rate of 100 mV s^-1^. Ferrocene (Sigma-Aldrich) was used as a known reference to calculate the E*_ox_* and E*_red_* values. Subsequently, the corresponding highest occupied molecular orbital (HOMO) and lowest unoccupied molecular orbital (LUMO) levels were calculated using the following Eq(s):

$$E_{LUMO}=\left[ \left( E_{red}- E_{1/2(ferrocene)} \right)+4.8 \right] \mathrm{eV}$$

$$E_{HOMO}=\left[ \left( E_{ox}- E_{1/2(ferrocene)} \right)+4.8 \right] \mathrm{eV}$$

**8. *In vitro* Zn^2+^ release and radical scavenging assays**

The *in vitro* Zn^2+^ release from the DNGM cryogel scaffold was quantified with or without NIR irradiation over a time frame of 0 to 168 hours. For this, approximately 100 mg of DNGM scaffold was placed in an Eppendorf tube containing deionized water (5 mL). After the specified time intervals, the scaffold was exposed to NIR light (1.0 W cm^-2^) for 10 minutes, and 1 mL of supernatant was collected. For the without NIR group, the same volume of liquid was removed at the designated time points. The Zn^2+^ release was measured using ICP-OES spectroscopy (Agilent-5900, Agilent Technologies).

The free radical scavenging properties of the fabricated cryogels were investigated using 2,2-diphenyl-1-picrylhydrazyl (DPPH) and $\cdot$OH radical clearance assays. For this, approximately 100 mg of each cryogel were incubated with 1 mL of 0.5 mM DPPH solution (Sigma-Aldrich) in the dark for 1 hour. The cryogel samples were centrifuged at 1500 ×*g* for 3 minutes, and the supernatant was collected for spectroscopic measurement at 517 nm. In the colorimetric $\cdot$OH clearance assay, 0.5 mL of 500 µM nitroimidazole (5-nIm, Sigma-Aldrich) and 0.5 mL of 400 µM of FeCl_2_ (Fisher Scientific) were mixed, followed by the addition of cryogel samples (approximately 100 mg each) and deionized water to achieve a final concentration of 250 µM of 5-nIm and FeCl_2_ in the solution. The resulting mixture was incubated for 5 minutes at room temperature in the dark. Next, 50 µL of the reaction mixture was taken out and mixed with 100 µL of Griess reagent (Sigma-Aldrich), followed by another incubation for 10 minutes. After that, the $\cdot$OH radical clearance performance (indicated by the production of red color) was quantified by recording the absorbance at 540 nm. For radical scavenging assays, ascorbic acid (0.5 mM) and deionized water served as positive and negative controls, respectively.

**9. *In vitro* antibacterial and antibiofilm assays**

The *in vitro* antibacterial performance of the developed cryogels was assessed with or without stimulation (808 nm, 1.0 W cm^-2^, 10 min) using O.D.-based proliferation assay, dilution plate culture assay, antibiofilm assay, bacterial membrane integrity assay, and SEM analysis. In this study, we used methicillin-resistant *Staphylococcus aureus* (*MRSA*, gram-positive, ATCC® BAA-41) and *Escherichia coli* (*E*. *coli*, gram-negative, KCTC®-2593) as model bacteria. The details of the experiments are as follows:

**9.1. Optical density (O.D.)-based viability assay**

For this, the bacteria stock solution (*E*. *coli*) was cultured overnight in nutrient broth and tryptic soy (*MRSA*) broth (Difco™, BD Biosciences) at 37 °C with gentle shaking. After achieving the desired turbidity (O.D._600 nm_ = 0.546), the bacterial solution (1 × 10^5^ CFU mL^-1^) was incubated with cryogel samples (~100 mg each) in an Erlenmeyer flask containing a total of 10 mL of media, and the O.D. value was measured over time (0-24 h). The DNGM group received NIR doses separately at each time interval. The PBS-treated group served as the control group. Each experiment was replicated three times (*n* = 3) and represented as an O.D.-based growth curve.

**9.2. Dilution plate culture assay**

To achieve this, the bacterial solution (1 × 10^5^ CFU mL^-1^) was incubated with cryogel samples (approximately 100 mg each) and cultured at 37 °C with gentle shaking (125 rpm) overnight. The PBS-treated group served as the control group. The DNGM group received NIR doses separately during and before plating. After 24 hours, bacteria from each group were collected and diluted five times with culture media. Next, 50 µL of the bacterial suspension (1 × 10^5^ CFU mL^-1^) was plated using nutrient agar (for *E*. *coli*, Difco™, BD Biosciences) and salt mannitol agar (for *MRSA*, BBL™, BD Biosciences), followed by uniform streaking. The plates were incubated at 37 °C for 24 hours, and colony formation was photographed and counted. Meanwhile, after the first experiment, cryogel samples were collected from each experimental group, washed three times with 1× PBS, sterilized under UV light, and the antibacterial test was performed again (up to three cycles) to assess the reusability of the cryogels. Each experiment was replicated three times (*n* = 3).

**9.3. Scanning electron microscopy (SEM) analysis**

To evaluate the effect of fabricated cryogel on the structural changes of bacteria with or without NIR, we examined the FE-SEM morphology of *MRSA* and *E*. *coli* after the desired treatment. For this, the bacterial solution (1 × 10^5^ CFU mL^-1^) was incubated with fabricated cryogels (~100 mg each) and treated with or without NIR for 24 hours. After 24 hours, the bacterial mass from each group was harvested by centrifugation (10,000 rpm) for 5 minutes, followed by fixation with 2% (*v*/*v*) glutaraldehyde/paraformaldehyde (Sigma-Aldrich) for 24 hours at 10 °C, washing with 1x PBS, dehydration using an ethanol series (30, 50, 70, 80, 90, and 100% *v*/*v*), and treatment with hexamethyldisilazane (Sigma-Aldrich) before drop-casting on mica films. Finally, the bacterial morphology was observed using an FE-SEM with an accelerating voltage of 5 kV cm^-1^ and a current density of 80-100 nA under gentle beam (GB) mode.

**9.4. Biofilm formation assay**

To accomplish this, the bacterial suspension (1 × 10^5^ CFU mL^-1^) was incubated with developed cryogels (~100 mg each) and cultured in a 24-well plate at 37 °C for 2 days to form thick biofilms. Subsequently, the DNGM group was additionally treated with NIR light for 10 minutes, after which all the samples were incubated at 37 °C for another 2-4 hours. The PBS-treated group served as the control. After the required incubation, the samples were carefully removed, followed by washing with 1× PBS and staining with a 0.4% (*w*/*v*) crystal violet stain (Sigma-Aldrich) for 5-10 minutes. Next, the excess stain was gently rinsed off with PBS and dried at room temperature. The biofilm formation in *MRSA* and *E*. *coli* was photographed, and the quantification of biofilm formation was measured spectrometrically (Infinite M Nano, Tecan) at 590 nm. Each experiment was repeated three times (*n* = 3).

**9.5. Membrane potential, protein leakage, and zinc quantifications**

For membrane potential measurement, the bacterial samples (both *MRSA* and *E*. *coli*) from each group after 24 hours of incubation were collected through centrifugation, resuspended in 1× PBS and subjected to zeta potential measurement (ZSP, Malvern Instruments). For the protein leakage study, the culture media from each group (*MRSA* and *E*. *coli*) were collected, centrifuged, and diluted five times in 1× PBS, and the protein amount was assessed using a BCA assay kit (AAT Bioquest, Inc.). The Zn^2+^ content in the bacteria after 24 hours was quantified using ICP-OES (Agilent 5900, Agilent Technologies). Each experiment was replicated three times (*n* = 3).

**10. *In vitro* biocompatibility and bioactivity tests**

**10.1. Viability and apoptosis assay**

The *in vitro* biocompatibility and regenerative capabilities of the fabricated cryogels were evaluated using human dermal fibroblasts (HDF), human keratinocyte (HaCaT), and human umbilical cord-derived endothelial (HUVEC) cells. The HaCaT and HDF cells were procured from the Korean Cell Line Bank (KCLB) at Seoul National University, South Korea. The HUVECs were purchased from American Type Culture Collection (ATCC^®^, PCS-100-013). The cells were cultured in DMEM media (Welgene) supplemented with 10% fetal bovine serum (FBS; Sigma-Aldrich) and 1% antibiotics (Thermo-Fisher Scientific) at 37 °C in a humidified atmosphere with 5% CO_2_. The cell viability and apoptosis of the HaCaT cells in the presence of fabricated cryogel with or without NIR were assessed using the WST-8 (Cellrix™, MediFab) and TUNEL (Thermo-Fisher Scientific) assays. Briefly, the cells (~1.5 × 10^4^ cells/100 µL/well) were incubated with cryogel samples (~50 mg each) in 96-well plates (SPL Life Sciences) for 1, 5, and 7 days with or without NIR. After the specified incubation period, the cell viability and apoptosis were evaluated using WST-8 dye and the TUNEL assay according to the manufacturer’s protocol. Additionally, the cytotoxicity of ZIF8 (0-100 µM) was also assessed using HDF, HaCaT, and HUVECs up to 7 days. The PBS-treated group served as the control group. Each experiment was replicated three times (*n* = 3). The details of the experimental groups are given as following:

| **Samples** | **Composition** | **Groups** |
| --- | --- | --- |
| Control | Only PBS treatment, -scaffold, -PTA, -NIR | Blank group |
| DN | Only DNA cryogel, -PTA, -NIR | Photoinactive groups |
| DNG | DNA/GelMA cryogel, -PTA, -NIR |  |
| DNGM | DNA/GelMA/MXene@ZIF8, +PTA, -NIR |  |
| DNGM + NIR | DNA/GelMA/MXene@ZIF8, +PTA, +NIR | Photoactive group |

**10.2. Live/dead assay**

We conducted a live/dead assay based on the initial viability assay to confirm cytocompatibility. To do this, approximately 100 mg of cryogel samples were incubated with HaCaT cells (about 2.5 × 10^⁴^ cells/mL/well) in 24-well plates (Corning®) for 7 days. The culture media were replaced every other day. After 7 days, the wells were carefully washed with 1× PBS and replenished with fresh DMEM media, followed by adding 10 µL of Calcein-AM/EtBr dye solution (5 mg/mL each, 1:1 ratio). The plates were then incubated for 10-15 minutes at 37 °C. Afterward, the dye solution was removed, and fresh 1× PBS was added to each well. Finally, the cells were photographed using an inverted fluorescence microscope (DMi8, Leica) with appropriate filters. The images were acquired using LAS-X (Leica) and processed with ImageJ (v1.8, NIH) software. Subsequently, the percentage of live cells was calculated and presented as mean ± s.d. of triplicate (*n* = 3) experiments.

**10.3. Transwell^®^ migration assay**

The effect of the engineered cryogel on the *in vitro* migration of HaCaT cells was examined using a Transwell^®^ cell migration assay. In brief, HaCaT cells (~2.5 × 10^4^ cells/500 µL) were incubated with cryogel samples in 40 µm Transwell^®^ inserts (Corning) using serum-free DMEM media. The lower chamber was then filled with DMEM media containing 10% FBS. The cells were permitted to migrate for 24 hours at 37 °C in a BOD incubator. The PBS-treated group served as the control. The DNGM group received separate NIR doses, which were administered three times (at 6, 12, and 24 hours) during the culture. After 24 hours, the upper chamber containing migrated cells was fixed with 100% methanol for 5 minutes, followed by staining with 0.5% (*w*/*v*) Giemsa stain (Sigma-Aldrich) for 10 minutes. The number of migrated HaCaT colonies (pinkish-violet color patches) was captured using an inverted optical microscope (Axio Vert. A1, Carl Zeiss) and quantified with ImageJ (v1.8, NIH) software. Five (*n* = 5) independent images were taken from each group for statistical analysis.

**10.4. Measurement of cellular oxidative stress**

The ROS scavenging potential of the fabricated cryogels in HaCaT cells was assessed using dichlorodihydrofluorescein diacetate (DCF-DA) staining method via H_2_O_2_ induced oxidative damage. Briefly, the HaCaT cells (~2.5 × 10^4^ cells/100 µL/96-well) were incubated with DN, DNG, and DNGM cryogels (~10 mg each). At day 1 and day 7, prior to NIR irradiation, the cells were treated with 100 µM H_2_O_2_ (Daejung Chemicals) for 30 min. Following that, the DNGM + NIR group was irradiated with 808 nm NIR light for 10 min at 37 °C. After that, the cells were washed with 1× PBS and supplemented with fresh media containing 40 µM of DCF-DA (Sigma-Aldrich) and incubated for 15-20 min in dark at 37 °C. Finally, the cells were washed with 1× PBS and then visualized using an invited fluorescence microscope (DMi8, Leica) with an excitation/emission of 485/520 nm. The images were captured with 20× objective lens and the FL intensity of DCF-DA was analyzed with Leica LAS-X software. For mechanistic approach, we additionally performed the qRT-PCR gene expression analysis of antioxidant genes (*Nrf2* and *HO-1*) at day 1 and day 7, respectively.

**10.5. Immunocytochemistry**

The morphology (F-actin and nuclear) and basic cytokeratin (KRT) expression in the presence of cryogel scaffolds were evaluated using immunocytochemistry (ICC) analysis. Briefly, HaCaT cells (~4 × 10^4^ cells/2 mL/well) were cultured with cryogel scaffolds (~100 mg) in 24-well plates for 7 days. Subsequently, the cells were washed with 1× PBS, then fixed with 3.7% paraformaldehyde (PFA; Sigma-Aldrich) for 10 minutes and permeabilized with 0.1% Triton X-100 (diluted in 1× PBS, Sigma-Aldrich) for 5 minutes at room temperature (RT). Following this, the cells were incubated with 1% bovine serum albumin (BSA; MP Biomedicals) for 1 hour, followed by incubation with AF-594 conjugated F-actin probe (Invitrogen) for 30 minutes at RT. For KRT staining, the cells were incubated with a primary antibody against KRT (0.2 µg mL^-1^, Santa Cruz Biotechnology) for 1 hour, followed by incubation with a FITC-conjugated secondary antibody (0.4 µg mL^-1^, Santa Cruz Biotechnology) for 1 hour at RT. After the desired incubation, the cells were washed with 1× PBS, and the nucleus was stained with DAPI (Sigma-Aldrich) for 1 minute. Finally, the cells with scaffolds were washed with PBS, and images were captured using an inverted fluorescence microscope (DMi8, Leica) equipped with the appropriate green, red, and blue filters, respectively. The images were processed using LAS-X (Leica) software, and quantification analysis was performed with ImageJ (v1.8, NIH, Bethesda) software. Details of the antibodies and their dilutions are provided in **Table S4**. The F-actin and nuclear anisotropy was presented in terms of elongation index (AI) and calculated as following:

$$AI= \left( 1- \frac{b}{a} \right)$$

Where, *a* is the length of the major axis and *b* is the length of the minor axis. A value of 0 is considered as isotropic (=round) structure, while a value close to 1 was considered as anisotropic (elongated).

**10.6. Raybiotech human cytokine array**

The effect of fabricated cryogel on HaCaT secretome with or without NIR was studied using the Raybiotech^®^ human cytokine array C5 (Raybiotech^®^, Georgia) after 7 days of culture. Briefly, the cells (~4 × 10^4^ cells/10 mL/100 mm dish) were cultured in DMEM containing cryogel extracts (~50 mg mL^-1^) and 5 mM CaCl_2_ (Sigma-Aldrich) for 7 days, with the media replaced every other day. The PBS-treated group served as the control group. After the incubation period, the culture media (approximately 9.8 ± 0.16 mL) was collected and centrifuged at 3000 rpm to remove debris and dead cells. Next, the secretome was concentrated using a centrifugal filter unit (Millipore^®^). Before the array analysis, the quality of the secretome was assessed using standard sodium dodecyl sulfate-polyacrylamide gel electrophoresis (SDS-PAGE). Finally, the cytokine array was performed according to the manufacturer's guidelines. The identified protein spots were subsequently subjected to a STRING protein-protein interaction study to reveal their role in wound healing. Each experiment was replicated three times (*n* = 3).

**10.7. EGF quantification assay**

Epidermal growth factor (EGF) was directly quantified from the HaCaT secretome at day 7 using a human EGF enzyme-linked immunosorbent assay (ELISA) kit (Thermo-Fischer Scientific), as per the manufacturer’s guideline.

**10.8. Flow cytometry**

The regenerative capabilities of the fabricated cryogels in HaCaT cells with or without NIR were evaluated by investigating the expression of cytokeratin markers (KRT5 and KRT14) using flow cytometry on day 7, as described in our previous study.^[^[^4^](#_ENREF_4)^]^

**10.9. qRT-PCR analysis**

To evaluate the expression of keratinization-specific gene markers (*Krt5*, *Krt10*, and *Krt14*) in HaCaT cells in the presence of fabricated cryogels with or without NIR, we performed quantitative real-time polymerase chain reaction (qRT-PCR) analysis. Total RNA was extracted from the HaCaT cells (~2.5 × 10^4^ cells/mL/24-well) after 7 days of culture using the Trizol^®^ (Sigma-Aldrich) method, and cDNA synthesis was performed according to the manufacturer’s guidelines. Then, mRNA expression was analyzed using a thermocycler (CFX Maestro Real-time System, Bio-Rad), and relative expression was quantified using the ^ΔΔ^CT method. The *GAPDH* primer was used as a housekeeping gene, and the fold change of marker genes was calculated relative to the fold of *GAPDH*. All primers were designed using *Primer3 Plus* and obtained from Bioneer^®^ Corporation, South Korea. A list of gene primers used for qRT-PCR analysis is provided in **Table S5**.

**11. Macrophage polarization study**

The effects of fabricated cryogel scaffolds on lipopolysaccharide (LPS)-induced macrophage polarization were studied using RAW 264.7 cells *in vitro*. The polarization potential was assessed through ICC, morphometric observation, flow cytometry, and qRT-PCR analysis with or without NIR treatment after 24 hours of incubation. The impact of fabricated cryogel under photobiomodulation in macrophage cells was evaluated by examining the expression of macrophage-specific protein and gene markers after 24 hours of culture. For this, murine monocyte/macrophage (RAW 264.7) cells were used and obtained from KCLB, South Korea. Details of the experimental procedure are provided below:

**11.1. Immunocytochemistry**

For this, the RAW 264.7 cells (~2.5 × 10^4^ cells/mL/24-well) were cultured in DMEM media with fabricated cryogels with or without NIR stimulation for 24 hours. After that, the cells were washed with 1× PBS, followed by fixation with 3.7% PFA (Sigma-Aldrich) for 10 minutes and permeabilization with 100% ice-cold methanol (Sigma-Aldrich) for 5 minutes at room temperature. Next, the cells were blocked using 1% BSA (Sigma-Aldrich) for 30 minutes, followed by the addition of an F_c_ receptor blocker (1:10, diluted in 1% BSA, eBioscience™) for 30 minutes in the dark at room temperature. After blocking and washing, the cells were incubated with primary antibodies against iNOS and CD163 (0.2 µg mL^-1^, Santa Cruz Biotechnology) for 1 hour, followed by incubation with FITC- and AF594-conjugated secondary antibodies (0.4 µg mL^-1^, Santa Cruz Biotechnology) for 1 hour at room temperature. Finally, the nucleus was counterstained with DAPI, and the expression of M1/M2 markers was visualized using an inverted fluorescence microscope (DMi8, Leica) with appropriate filters. The images were processed with LAS-X (Leica) software, while fluorescence intensities were quantified using ImageJ (v1.8, NIH, Bethesda) software. For statistical analysis, at least ten (*n* = 10) individual images were captured from each group.

**11.2. Flow cytometry**

The flow cytometry was performed to investigate the canonical M1 (CD86, CD68, and iNOS) and M2 (Arg-1, CD206, and CD163) polarization markers expressed in RAW 264.7 cells after culturing with cryogels with or without NIR. The flow cytometric analysis was carried out according to a protocol described in our previous study.^[^[^5^](#_ENREF_5)^]^ Each marker's expression was replicated three times (*n* = 3) in different groups.

**11.3. qRT-PCR analysis**

To validate the macrophage polarization potential of the fabricated cryogels, we next evaluated the expression of M1/M2 macrophage-specific gene markers (*e*.*g*., *iNOS* and *CD163*) after 24 hours of culture. Briefly, RAW 264.7 cells (approximately 4 × 10^4^ cells/1 mL/24-well plate) were incubated with the fabricated cryogel samples (around 100 mg each) with or without NIR stimulation. After 24 hours, the cells were washed with 1× PBS and harvested for RNA extraction using the Trizol^®^ (Sigma-Aldrich) method, and cDNA synthesis was performed following the manufacturer’s guidelines. The qRT-PCR was conducted as described previously. All primers were designed using *Primer3 Plus* and obtained from Bioneer^®^ Corporation, South Korea. A list of gene primers used for qRT-PCR analysis is provided in **Table S6**.

**12. Hemolysis study**

The *ex-vivo* biocompatibility of the fabricated cryogel was assessed using a red blood cell (RBC) hemolysis assay. Fresh blood from healthy rats (3-4 weeks old) was washed multiple times with 1× PBS and randomly divided into five groups: positive control (Triton-X 100), negative control (PBS only), DN, DNG, and DNGM, respectively. Approximately 6× blood (1 mL) was combined with ~100 mg of cryogel samples and incubated at 37 °C for 120 minutes. Subsequently, the blood samples were removed and centrifuged at 1000 r.p.m. for 5 minutes to separate the RBCs. Next, the supernatant (100 µL) was collected, and absorbance was measured spectrometrically at 540 nm. The percentage of hemolysis was calculated using **Eq. (1)**:

$$\% hemolysis= \frac{({Abs}_{t}- {Abs}_{n.c.})}{({Abs}_{p.c.}- {Abs}_{n.c.})} \times100\%$$

Where *Abs_t_*, *Abs_p.c._*, and *Abs_n.c._* are the absorbance of the sample, positive control, and negative control groups, respectively.

**13. Bulk RNA sequencing**

The transcriptomic changes in HaCaT cells in the presence of fabricated cryogels were investigated using bulk RNA sequencing (RNA-Seq) analysis after 7 days of culture. Briefly, HaCaT cells (~4 × 10^4^ cells/mL/24-well) were cultured in keratinocyte differentiation media (DMEM media supplemented with 10% fetal bovine serum, five mM calcium chloride, and 1% antibiotics) with DN and DNGM cryogels (~500 mg each). The DNGM group was additionally irradiated with NIR (1.0 W cm^-2^, 5 min) every other day. The experimental groups were control (no hydrogel), DN, DNGM, and DNGM + NIR, respectively. After the desired time, total RNA was extracted using the Trizol^®^ (Sigma-Aldrich) method and subsequently prepared for RNA library creation. An RNA integrity number (TapeStation, Agilent) above 9.0 was selected for the 3’ UTR of RNA for RNA-Seq analysis. Finally, QunatSeq 3’ mRNA-Seq was conducted using a next-generation sequencer (NextSeq 550, Illumina^®^) and compared with a reference genome (GRCh38, human). The raw data underwent extensive quality control (FastQC) and was processed using Excel-based differential gene expression analysis (ExDEGA, v5.2.1, ebiogen Corp.) software. The gene expression data was depicted as fold change (Log2 FC, ^*^*p* < 0.05, *t*-test) between treatment *vs*. control and normalized using the (TMM + CPM normalized RC+1, edgeR) method. The differentially expressed gene (DEG) expression involved in wound healing, cell differentiation, epidermis development, and ECM remodeling was analyzed by *k*-means hierarchical clustering (Log2 FC, ^*^*p* < 0.05, *t*-test) and heatmaps created using iDEP 2.0 (South Dakota State University) software. Additionally, gene ontology (GO) and Gene Set Enrichment Analysis (GSEA) were performed to evaluate significant enrichment in various pathways and their key gene markers using web-based bioinformatics tools, including the Kyoto Encyclopedia of Genes and Genomes (KEGG), g.Profiler (ELIXIR Infrastructure), DAVID, and GSEA (v4.3.3, UC San Diego) linked to MSigDB, respectively. Highly correlated DEGs across all samples were analyzed to identify hub genes using Weighted Gene Co-Expression Network Analysis (WGCNA) with R packages. The 2D and 4D principal component analysis (PCA) was conducted to visualize the variance of DEGs across different samples.

The most significant DEG expressions related to keratinization and wound healing were PCR-validated using HaCaT cells. Moreover, the effect of released Zn^2+^ from the DNGM cryogel under NIR stimulation and its impact on keratinization and angiogenesis was also validated by using an intracellular Zn^2+^ chelator, *e*.*g*., diethylenetriaminepentaacetic acid (DEPTA, Sigma-Aldrich) and knockdown of Znf648 (miR-Znf648) *in vitro*. Briefly, the desired concentration of HaCaT cells (~4 × 10^4^ cells) were incubated with the fabricated cryogels and DEPTA (100 µM). After 24 h, the migrated HaCaT colonies were stained with 1% crystal violet and the images were captured with an optical microscope. For immunostaining, the HaCaT cells (~1.5 × 10^4^ cells) were cultured for 7 days and then stained with anti-KRT14 antibody (1:250, SCBT) and visualized using a confocal microscope (LSM880, Zeiss). For angiogenesis study, the HUVECs (~2.5 × 10^4^ cells/500 µL/well) were incubated with the fabricated cryogels and DEPTA (100 µM) for 24 h. The Matrigel^®^ (Corning) was taken as standard control. After 24 h, the 3D angiogenic sprouts growth was photographed and the sprouts length was quantified using ImageJ (v1.8, NIH).

For knockdown experiments, HaCaT cells (60-70% confluency) were treated with 100 nM miR-Znf648 (*sense*: 3′-CCUCAUACUUCUAGACCUC-5′ and *antisense*: 5′-GAGGUCUAGAAGUAUGAGG-3′) with Lipofectamine 3000 (Thermo-Fischer Scientific) supplemented with 500 µL of Opti-MEM™ (Gibco-BRL) in a 24-well plate and incubated at 37 C for 6 h. Following that the cells were washed with PBS and incubated with serum-supplemented DMEM overnight, prior to the cell migration and qRT-PCR assay. Control miRNA (=miR-Con) was also tested as a positive control. The miRNAs were obtained from Bioneer^®^ Corporation, South Korea.

**14. *In vivo* wound healing study**

The therapeutic effects of the engineered cryogels were examined using rat diabetic wound and infected wound models. We chose *MRSA* for wound infection due to its drug-resistant properties. Prior to wound healing study, all the rats received an intraperitoneal injection of streptozotocin (200 mg kg^-1^, Sigma-Aldrich) to induce hyperglycemia. The Institute of Animal Care and Use Committee (IACUC) approved the animal study at the Korea National University of Transportation (KNUT-2024-A7). After the specified time, the wound healing effectiveness of the cryogels with or without NIR was assessed using Hematoxylin and Eosin (H&E) staining, Masson’s Trichrome (MT) staining, immunocytochemistry (ICC), and qRT-PCR analysis. Details of the animal experiment procedure are provided below:

**14.1. Biosafety evaluation *in vivo***

The in vivo biosafety of the MXene, MXene@ZIF8, and fabricated cryogels was assessed using in vivo imaging system (IVIS, whole body and organ-level), contact allergy (=irritant toxicity) test, H&E staining, and routine blood biochemistry up to 21 days (3 weeks). For the biodistribution analysis MXene and MXene@ZIF8 (100 µg mL^-1^ each) was administrated intravenously (*ex vivo* organ accumulation) and dorsal skin of rats (whole body) and the IVIS spectrum (Spectral LAGO X) was recorded at various time points to study the blood clearance. Following that, 0.01 mg mL^-1^ FITC (Sigma-Aldrich) loaded cryogel scaffolds (10 × 5 mm^2^) was also implanted inside the dorsal skin and the *in vivo* systemic biodegradation up to 21 days using IVIS system. At day 21, major organs (heart, lung, kidney, spleen, and liver) were collected and subjected to histomorphology analysis to examine any anomalies in the organs. Moreover, the blood biochemistry profile (WBC, RBC, MPV, HGB, PLT, MCH, MCV, and HCT) of the rats after scaffold implantation was assessed at day 21.

**14.2. Subcutaneous wound model and *MRSA* infection**

The *in vivo* wound healing study utilized 6-week-old imprinted control region (ICR) male mice. The mice were randomly divided into five groups: control (no cryogel, *n* = 10), DN (*n* = 10), DNG (*n* = 10), DNGM (*n* = 10), and DNGM + NIR (*n* = 10). They were housed in a controlled photoperiod room (12 h light and 12 h dark) with ad libitum access to food and water. Initially, the mice’s hair was removed from the skin using a clipper and depilatory cream. Next, a 10 mm diameter wound was created using a biopsy punch on the skin after hair removal. Following this, 10 µL of *MRSA* (1 × 10^8^ CFU ml^-1^) was injected directly into the wound, while the control group received only 50 µL of 1× PBS. After placing the cryogel scaffolds (10 × 5 mm^2^) on the wound, the scaffold was irradiated with a NIR laser (1.0 W, 808 nm) for 10 minutes to ensure mild heat generation and adequate release of Zn^2+^ from the MXene@ZIF8 in the DNGM group. NIR treatment was conducted every other day for a period of 21 days, and wound photographs were taken to document macroscopic wound healing. The *in vivo* NIR heat production was monitored with a thermal camera (E6390, FLIR), and the Zn^2+^ release in the wound tissue was quantified using ICP-OES (Agilent 5900, Agilent Technologies). On days 7 and 14, the mice were sacrificed and underwent H&E and MT staining, while cytochemical staining was performed on days 14 and 21 to visualize skin re-epithelialization and wound remodeling.

**14.3. Immunocytochemistry**

For this, the wound tissue samples from various groups were fixed in neutral buffered 10% PFA solution (Sigma-Aldrich), embedded in paraffin, and sectioned into five μm-thick slices, following standard protocols described previously. We used specific antibodies for epidermal markers (KRT5, KRT10, and KRT14), dermal markers (FBN, COL1A, and Vimentin), and myofibroblast markers (Desmin and α-SMA) staining at days 14 and 21, respectively. Images were captured using an inverted fluorescence microscope (DMi8, Leica), and fluorescence intensities were quantified using ImageJ (v1.8, NIH, Bethesda). Details of the antibodies and their dilution factors are provided in **Table S2**.

**14.4. Tensile strength measurement**

The tensile strength of the healed wound in various groups was measured using a pressure transducer connected with an amplifier recorder (Pclab-UE+ system, China). The 5 × 10 mm² skin strip was held at the wound edge and gradually dragged in the opposite direction until it fractured. The tensile strength was calculated by dividing the breakpoint strength by the cross-sectional area. Each experiment was replicated five (*n* = 5) times and data is reported as mean ± s.d. of replicated experiments.

**14.5. qRT-PCR study**

The single-cell suspension was prepared to evaluate the levels of inflammatory gene markers (*iNOS*, *TNF-α*, *CD163*, and *IL-10*) on day 14, while the expression of skin remodeling-associated gene markers (*Fbn*, *Col1A*, *Krt5*, *Krt10*, *Wnt10b*, *Mmp9*, and *Timp1*) was assessed on days 14 and 21, respectively. Briefly, wound bed skin samples were cut into small pieces measuring 2 mm² using a biopsy punch and were subsequently washed with 1× PBS. After washing, the tissue samples were digested with 0.3% dispase-II enzyme (Sigma-Aldrich) at 37 °C for 90 minutes. The dermis samples were digested with a balanced buffer containing collagenase D (1 mg/mL, Sigma-Aldrich), hyaluronidase (1 mg/mL, Sigma-Aldrich), and DNase (150 U/mL, Sigma-Aldrich) in a water bath maintained at a constant temperature of 37 °C for 2 to 3 hours. The digested samples were filtered through a 70 µm strainer (Corning^®^) and centrifuged to obtain cell pellets. The pellet was resuspended in 1× PBS and centrifuged again to discard debris. The resulting cell pellet was directly used for Trizol-based RNA extraction (Sigma-Aldrich) and subsequent qRT-PCR analysis.

**15. Software and data processing**

The raw imaging data (ICC and histology) were processed with LAS-X (Leica) and ImageJ (v1.8.0, NIH). Cell sorting analysis was performed using FlowJo™ (v10.0) software. Graph preparation was conducted with Origin Pro (v9.0, Origin Labs). Differentially expressed gene (DEG) expression and comparison analysis were performed using ExDEGA (v5.2.1, ebiogen). The qRT-PCR data analysis was executed in CFX Maestro v1.1 (Bio-Rad). All figures were prepared with Microsoft PowerPoint (v2016) or Adobe Photoshop (CS6) unless stated otherwise.

**16. Statistical analysis**

Statistical analysis was conducted using Origin Pro v9.0 (Origin Labs). The means between the control and treatment groups were compared using One-way Analysis of Variance (ANOVA) with Tukey’s HSD *post-hoc* test. Data are presented as mean ± standard deviation (s.d.) from replicated experiments, and statistical significance was determined at ^*^*p* < 0.05, ^**^*p* < 0.01, ^***^*p* < 0.001, and ^****^*p* < 0.0001. Statistically non-significant data are indicated as *n*.*s*.


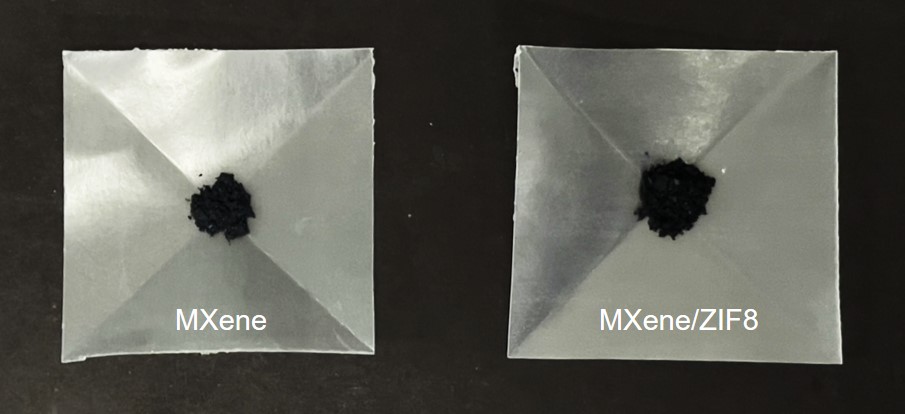


**Figure S1. (a)** Digital photographs of the MXene (Ti_3_C_2_T_x_) and MXene@ZIF8.


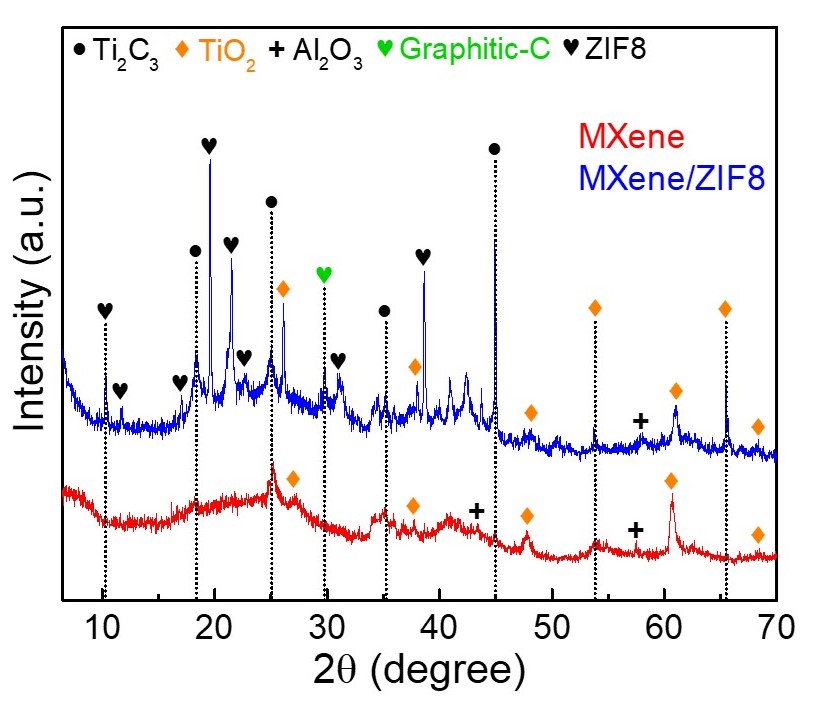


**Figure S2.** XRD spectra of the MXene and MXene@ZIF8 composites.

**
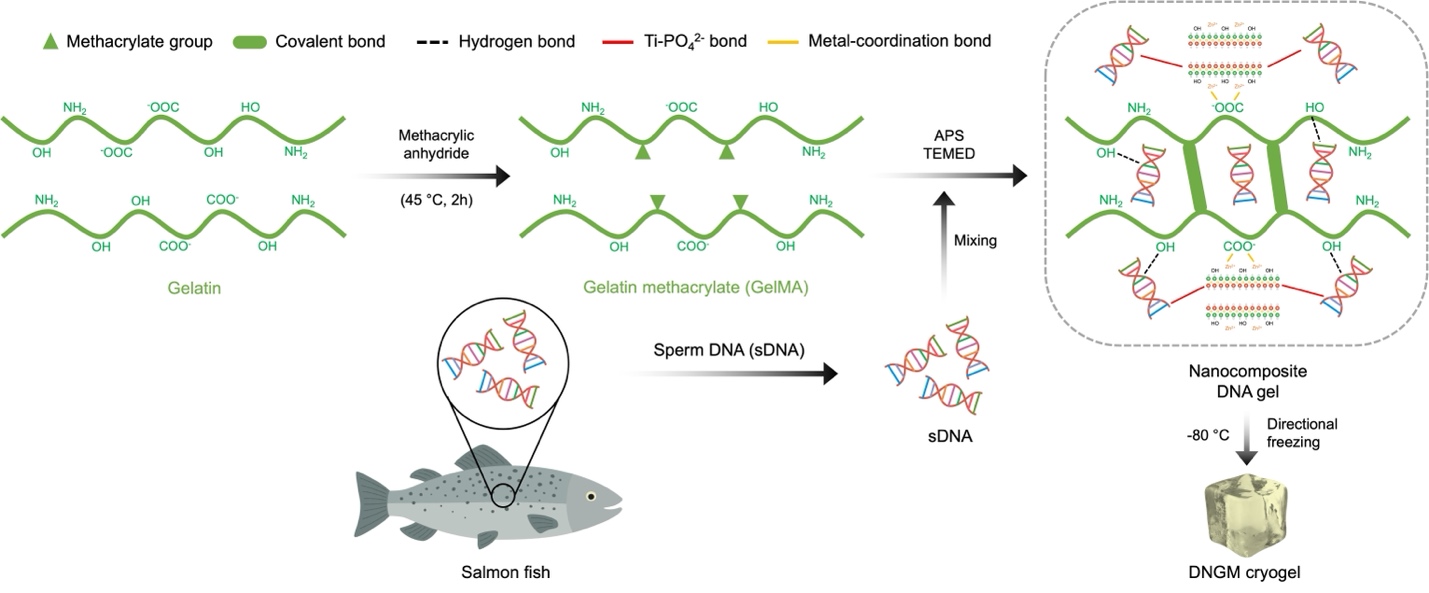
**

**Figure S3.** Schematic illustration of the detailed fabrication process of MXene@ZIF8 integrated DNA cryogels.


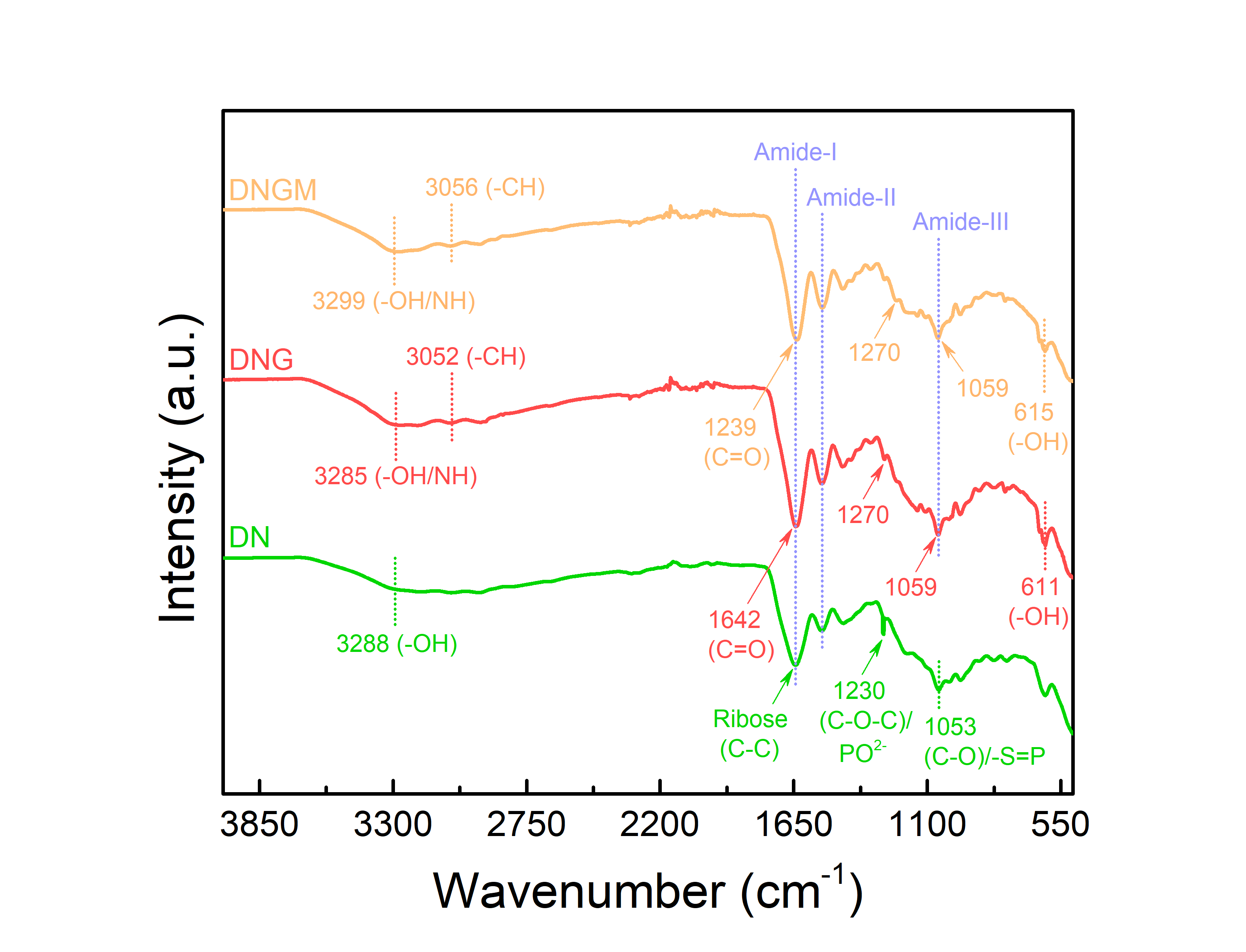


**Figure S4.** FT-IR spectra of the DN, DNG, and DNGM cryogel scaffolds.

**
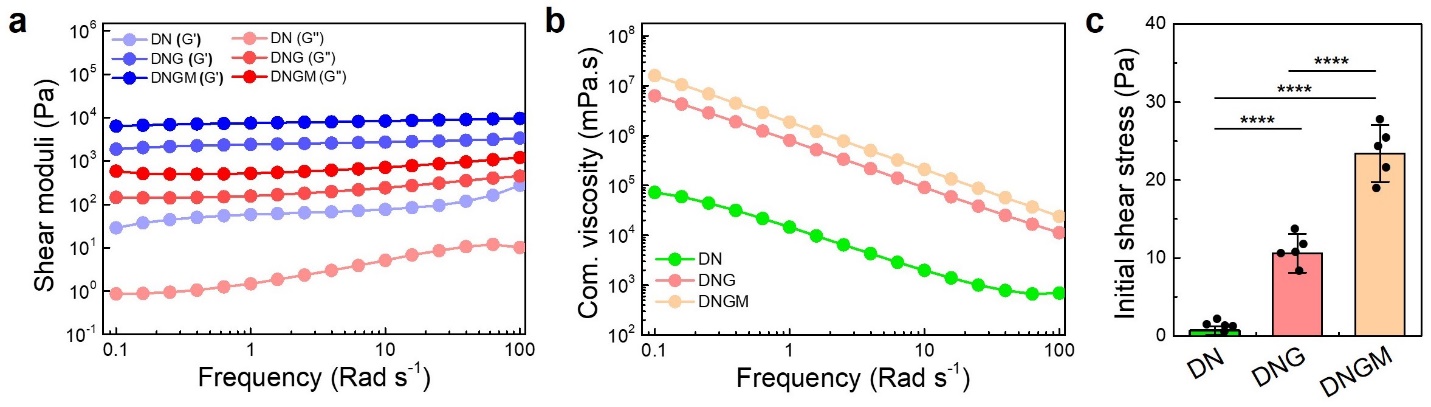
**

**Figure S5.** Viscoelastic properties of the developed cryogels. **(a)** Representative storage (G′) and loss (G′′) modulus of the DN, DNG, and DNGM cryogels under varying frequency ranges (0.1-100 Ras s^-1^). **(b)** Complex viscosity (*η**) measurement of the developed cryogels under varying frequencies. **(c)** Calculation of initial shear stress (Pa) at 0.1 s^-1^. Data reported as mean ± s.d. of triplicated experiments, statistical significance considered at ^****^*p* < 0.0001 (One-way ANOVA with by Tukey’s HSD *post-hoc* test).


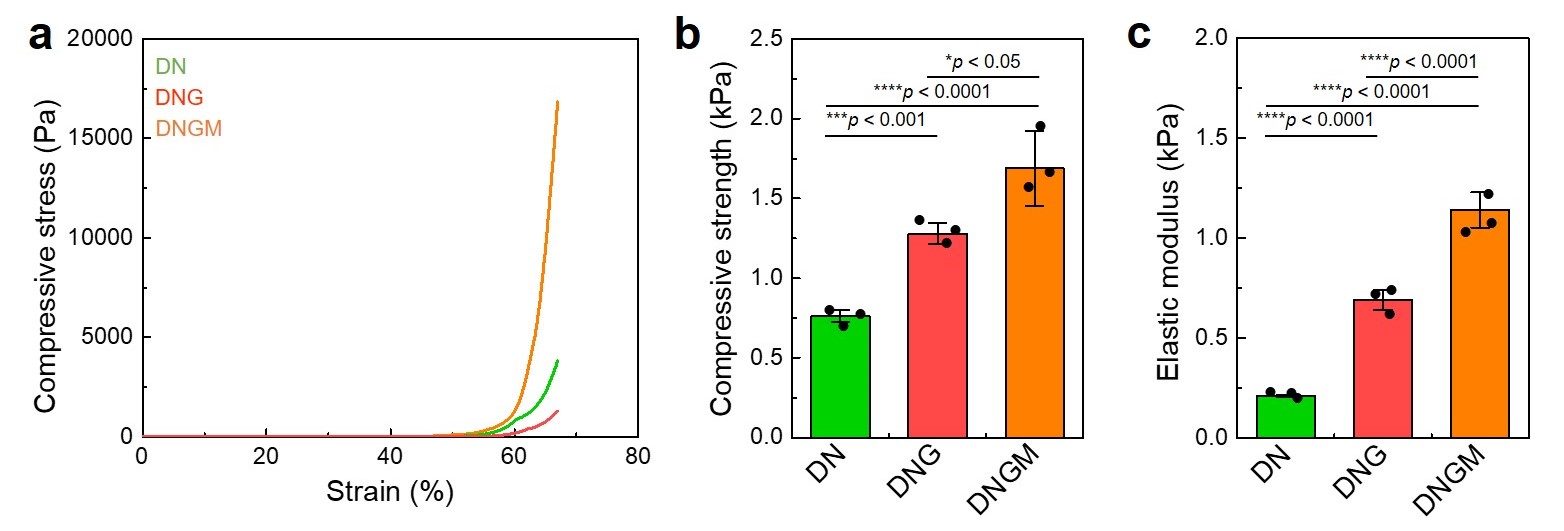


**Figure S6.** Mechanical properties of the fabricated cryogel scaffolds. **(a)** Compressive stress-strain graph of the DN, DNG, and DNGM cryogel scaffolds at RT. **(b, c)** Statistical analysis of the compressive strength and elastic modulus of the fabricated cryogels. Data reported as mean ± s.d. of triplicated experiments, statistical significance considered at ^*^*p* < 0.05, ^***^*p* < 0.001, and ^****^*p* < 0.0001 (One-way ANOVA followed by Tukey’s HSD *post-hoc* test).

**
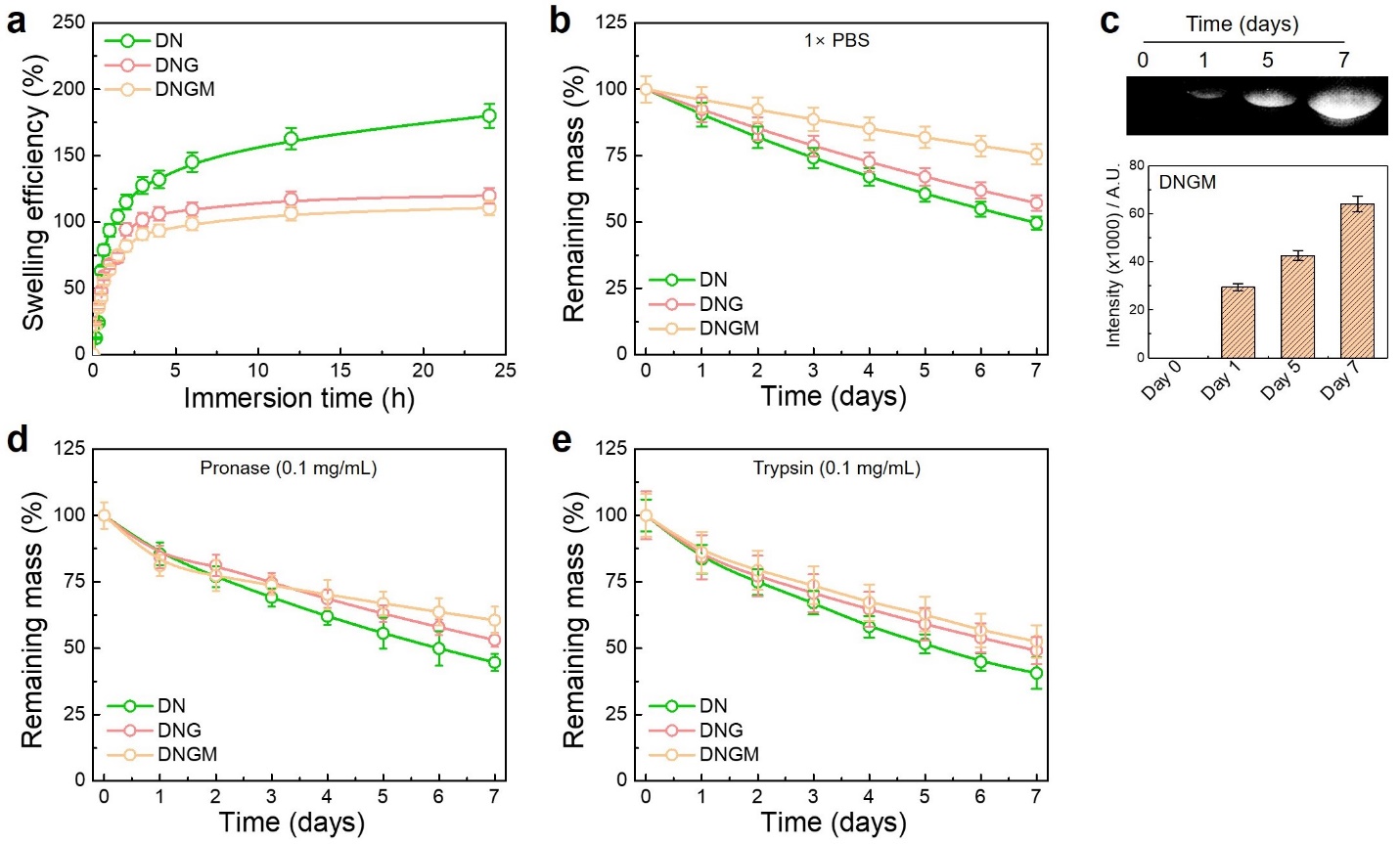
**

**Figure S7.** Swelling and degradation study of the fabricated cryogel scaffolds. **(a)** Swelling efficiency of the fabricated cryogel scaffolds in 1× PBS at 37 °C up to 24 h. **(b)** Degradation study of the cryogel scaffolds in 1× PBS at 37 °C up to 7 days. **(c)** Denaturation PAGE analysis with corresponding intensity values of the released DNA from DNGM cryogel at indicated time points. **(d, e)** Degradation study of the cryogel scaffolds in enzyme solution (pronase and trypsin, 0.1 mg mL^-1^ each) at up to 7 days.

**
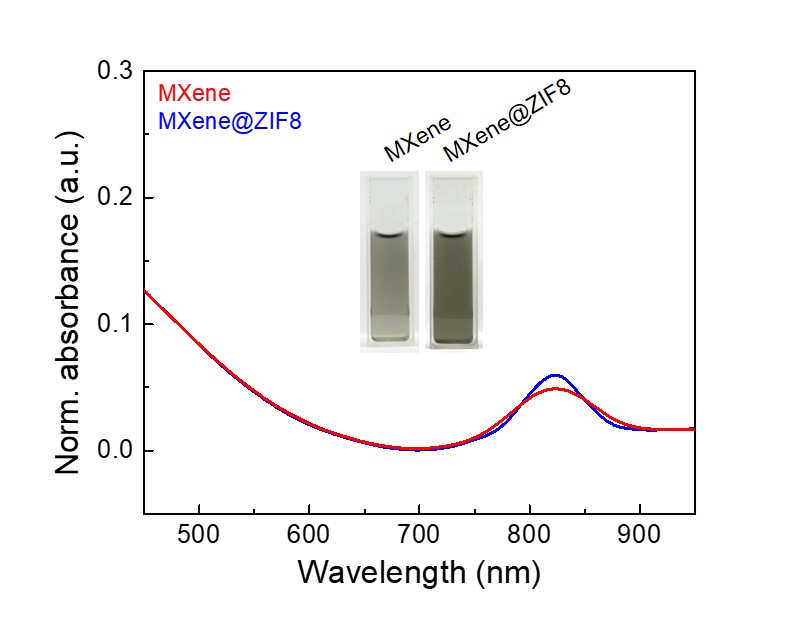
**

**Figure S8.** UV-Vis near-infrared (NIR) absorption spectra of the MXene and Mxene@ZIF8 (100 µg mL⁻¹ each) at room temperature.


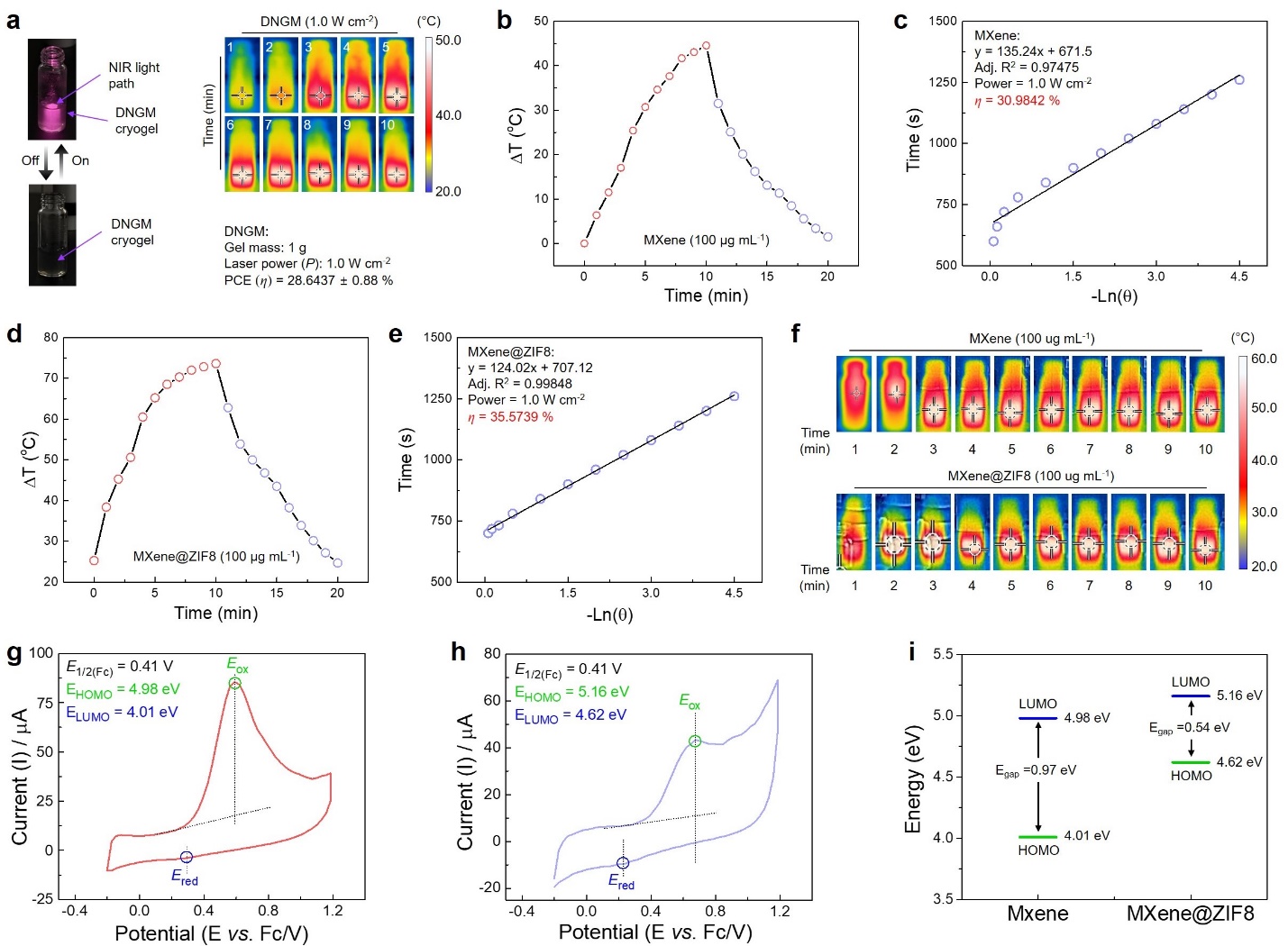


**Figure S9.** Photothermal properties of MXene and MXene@ZIF8. **(a)** Digital and thermal photographs of the DNGM cryogels illustrating the time-dependent temperature release profile under 808 nm NIR irradiation (1.0 W cm^-2^). **(b, c)** Photothermal heating and cooling curves of MXene and MXene@ZIF8 (100 µg mL^-1^) at ambient temperature. **(d, e)** Calculation of the photothermal conversion efficiency (PCE) for MXene and MXene@ZIF8. **(f)** Representative thermal images of MXene and MXene@ZIF8 (100 µg mL^-1^ each) at specified time points. **(g-i)** Experimental determination of HOMO-LUMO energy levels and band gaps (*E*_g_) of the pristine MXene and MXene@ZIF8.

**
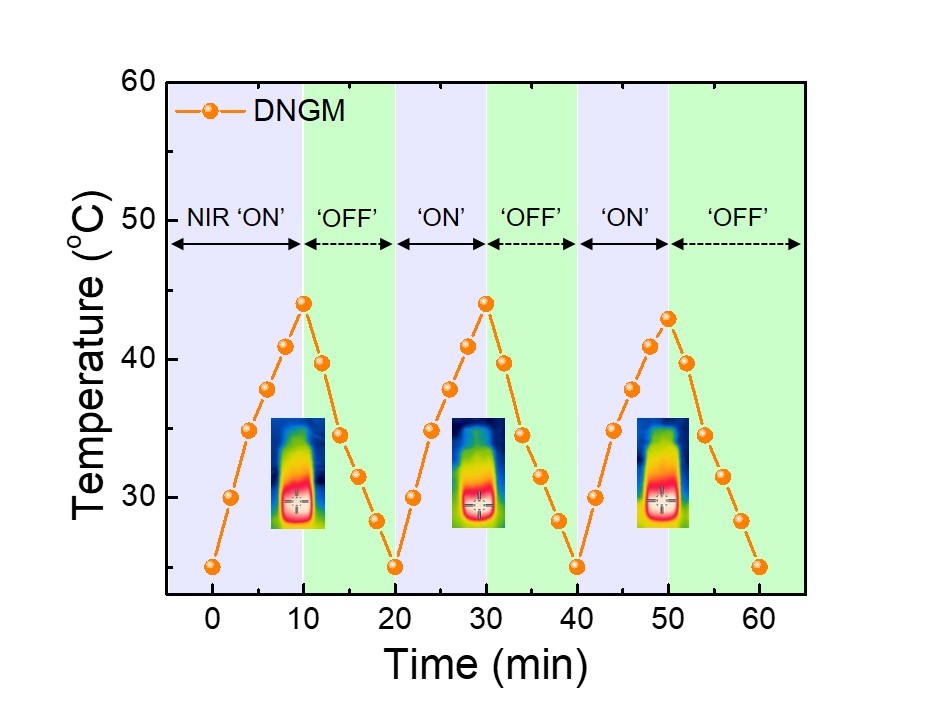
**

**Figure S10.** Cyclic photothermal responsiveness and stability of the DNGM cryogels after three NIR ‘ON’/’OFF’ cycles.

**
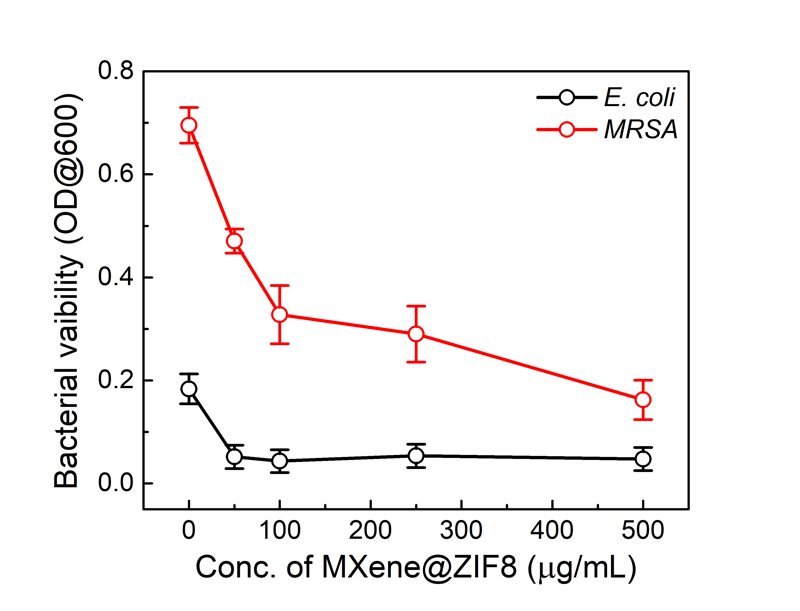
**

**Figure S11.** Optical density (O.D.)-based viability analysis of *E. coli* and *S. aureus* (MRSA) upon treatment with MXene@ZIF8 (0-500 µg mL^-1^) after 24 h of culture.

**
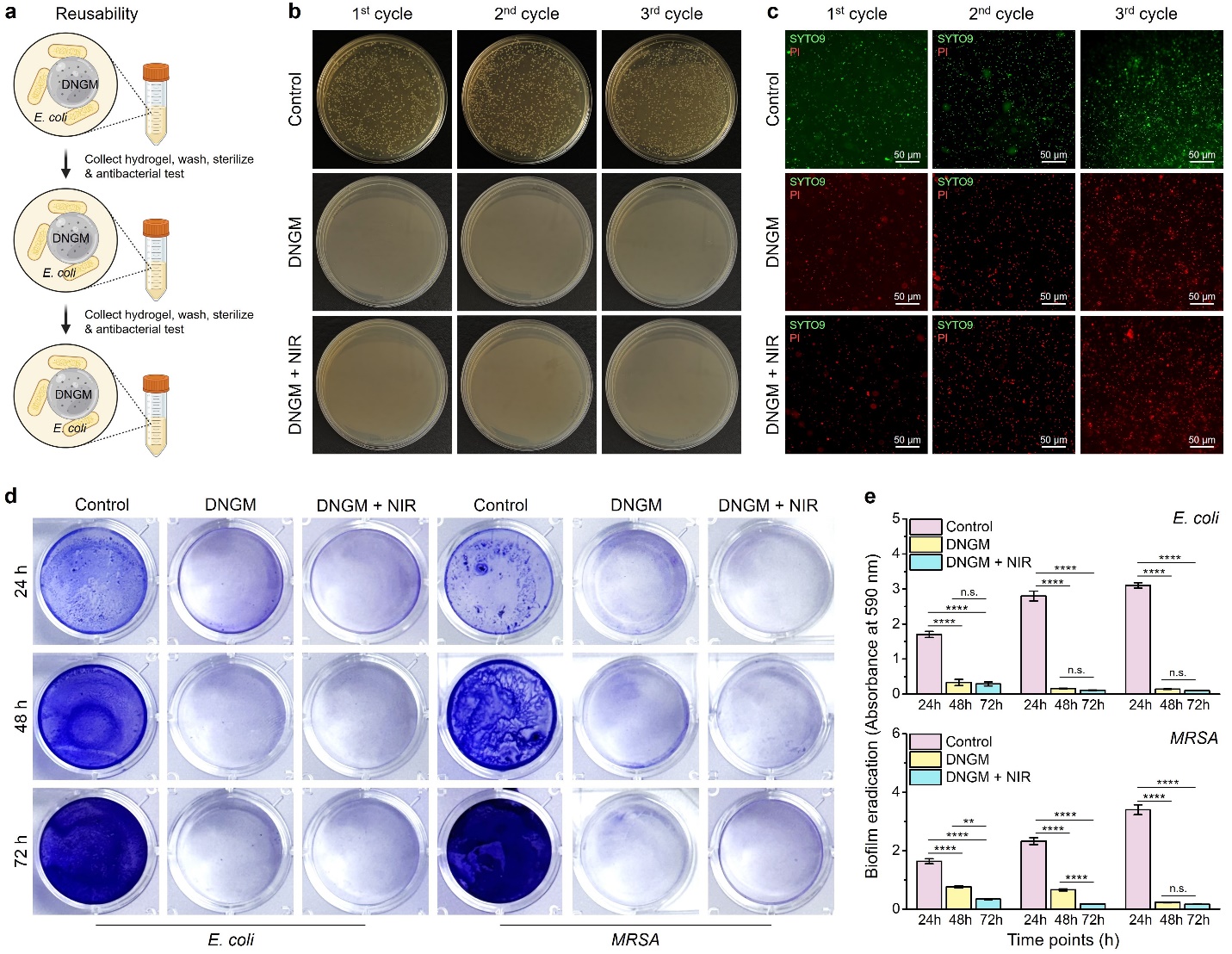
**

**Figure S12.** Long-term photothermal antibacterial performance of the DNGM cryogels against *E. coli* and *MRSA*. **(a)** Schematic illustration of the experimental procedure of the DNGM cryogel w/ or w/o NIR. **(b, c)** Digital photographs and FL microscopy images showing the colony formation and viability (live/dead; SYTO9/PI) of *E. coli* in the presence of DNGM cryogels w/ or w/o NIR (1.0 W cm^-2^) treatment. After first use, the DNGM cryogels (~80 mg) were centrifuged, washed several times with 1× PBS, and sterilized using UV light, and the antibacterial performance was tested with *E. coli* (1 × 10^5^ CFU mL^-1^). The control group bacteria were treated with PBS only. **(d)** Representative digital photographs of the biofilm eradication assay against *E. coli* and *MRSA* after repeated NIR irradiation (1.0 W cm^-2^) up to 72 h *in vitro*. **(e)** Statistical analysis of biofilm eradication assay (*n* = 3 each). Data reported as mean ± s.d. of replicated experiments, statistical significance considered at ^**^*p* < 0.01 and ^****^*p* < 0.0001 (One-way ANOVA with Tukey’s HSD *post-hoc* test). Non-significant data represented as *n*.*s*.

**
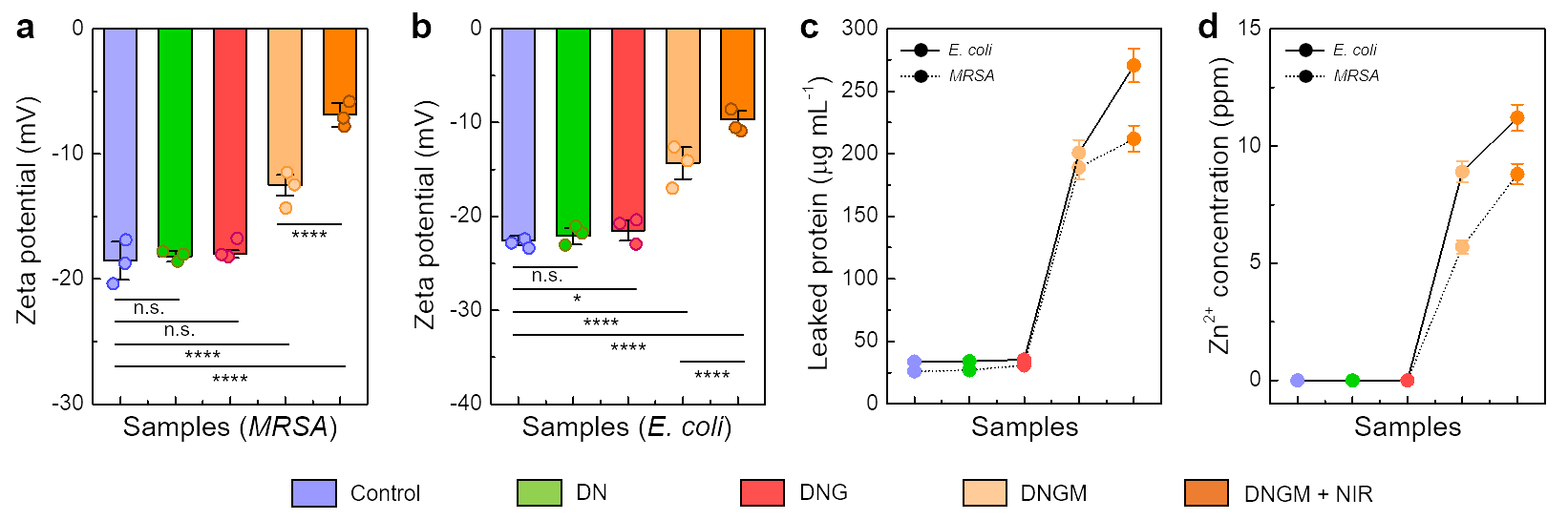
**

**Figure S13.** Quantitative evaluation of antibacterial properties using various formulations after 24 h. **(a, b)** Zeta potential (mV) of *MRSA* and *E. coli* after sample treatment (*n* = 3 each). **(c)** Analysis of protein leakage from *MRSA* and *E. coli* after sample treatment (*n* = 3 each). **(d)** ICP analysis for Zn^2+^ content in *MRSA* and *E. coli* after sample treatment (*n* = 3 each). Data reported as mean ± s.d. of replicated experiments, statistical significance considered at ^*^*p* < 0.05 and ^****^*p* < 0.0001 (One-way ANOVA followed by Tukey’s HSD *post-hoc* test).

**
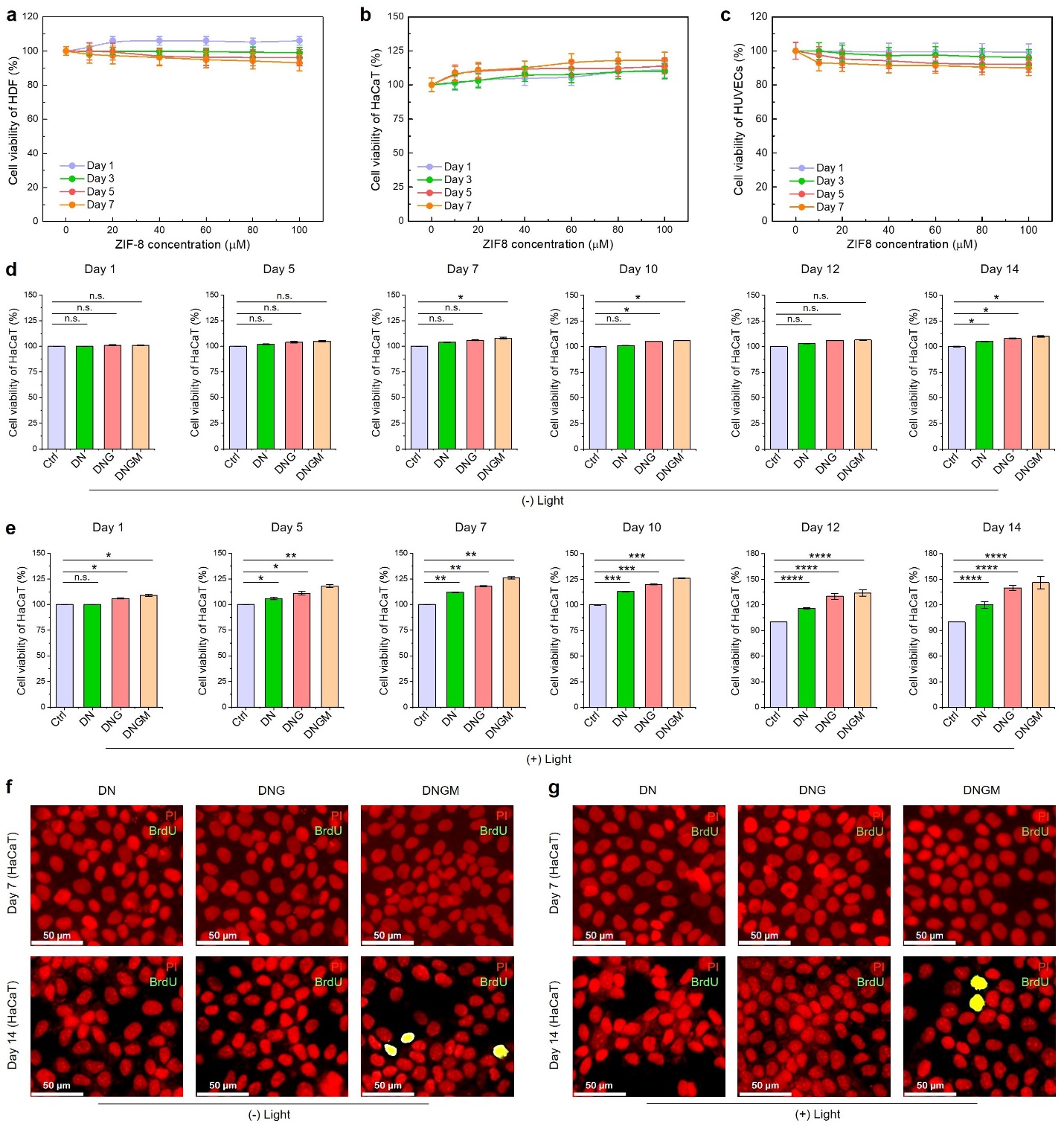
**

**Figure S14.** *In vitro* biocompatibility test of human cell lines w/ or w/o phototherapy. **(a-c)** WST-8 cell viability assay results of HDF, HaCaT, and HUVECs with varying concentration of ZIF8 (0-100 µM) up to 7 days. **(d, e)** Cytotoxicity assays of HaCaT cells with various formulations w/ or w/o NIR (1.0 W cm^-2^, 10 min, 808 nm NIR light) at 7 and 14 days. **(f, g)** Cellular apoptosis study of HaCaT cells in various formulations after 7 and 14 days of treatment using terminal deoxynucleotidyl transferase (TUNEL) assay. Scale bar: 50 µm. Data reported as mean ± s.d. of triplicated experiments, statistical significance considered at ^*^*p* < 0.05, ^**^*p* < 0.01, ^***^*p* < 0.001, and ^****^*p* < 0.0001 (One-way ANOVA followed by Tukey’s HSD *post-hoc* test).


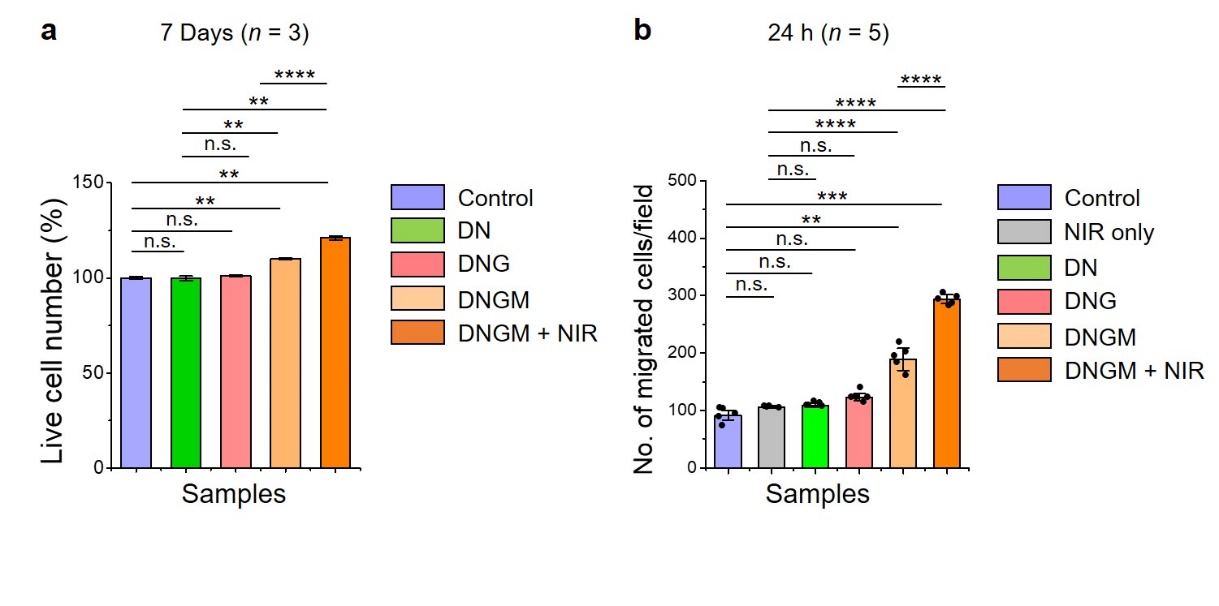


**Figure S15.** Statistical analysis of live/dead assay and Transwell™ cell migration assay. Data reported as mean ± s.d. of replicated experiments, statistical significance considered at ^*^*p* < 0.05, ^**^*p* < 0.01, ^***^*p* < 0.001, and ^****^*p* < 0.0001 (One-way ANOVA test with Tukey’s HSD *post-hoc* analysis).

**
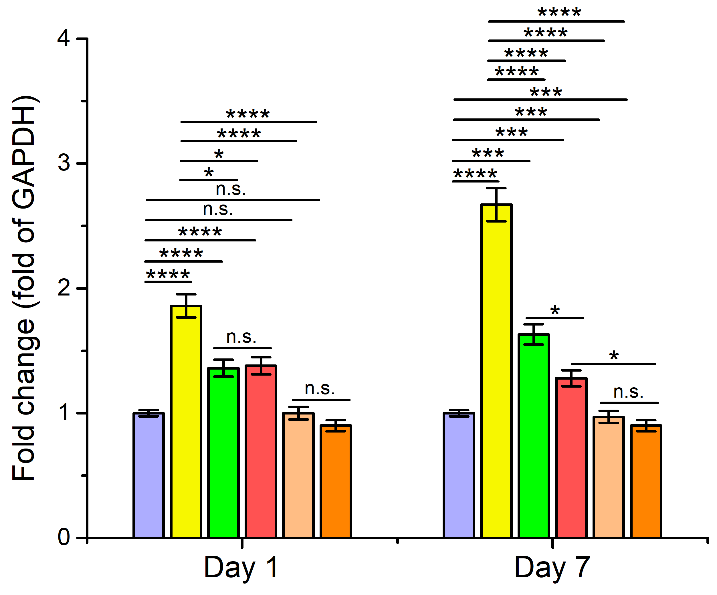
**

**Figure S16.** qRT-PCR results of *HO-1* (ROS sensitive) gene markers expression in HaCaT cells in the presence of fabricated cryogels *in vitro* (*n* = 3 each). Data reported as mean ± s.d. of replicated experiments, statistical significance considered at ^*^*p* < 0.05, ^***^*p* < 0.001, and ^****^*p* < 0.0001 (One-way ANOVA test with Tukey’s HSD *post-hoc* analysis).

**
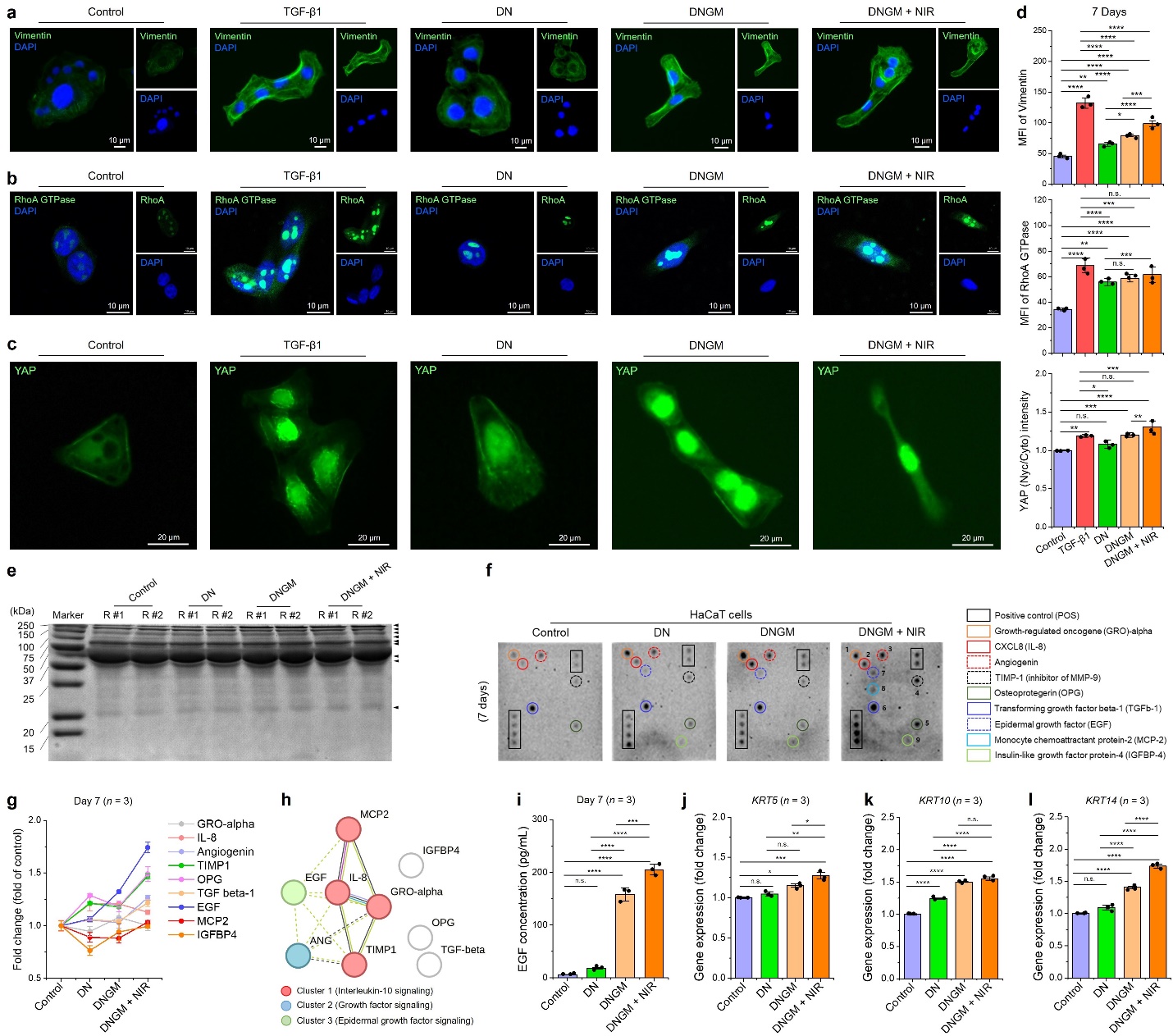
**

**Figure S17. (a-c)** Representative CLSM images of the HaCaT cells showing the expression of key EMT markers, *e*.*g*., Vimentin (green), RhoA GTPase (Green), and YAP (green) at day 7. Scale bar: 10 and 20 µm. **(d)** Statistical analysis of mean FL intensities of the Vimentin, RhoA GTPase, and YAP. Secretome analysis of HaCaT cells after 7 days of cell culture. **(e)** Representative SDS-PAGE analysis of the HaCaT cell secretome after 7 days of culture in differentiation media. R#1 and R#2 indicate the replicates from each group. **(f)** A representative antibody array of the HaCaT cell secretomes in various groups shows the expression of secreted cytokines after 7 days of culture. **(g)** Raybiotech human cytokine array analysis of HaCaT cell secretome showing the expression of various cytokines associated with skin wound healing at day 7 (*n* = 3 each). **(h)** STRING protein-protein interaction study of major proteins identified from HaCaT secretome. **(i)** ELISA quantification of EGF secreted from HaCaT cells in various treatment groups at day 7 (*n* = 3 each). **(j-l)** qRT-PCR results of the epidermis-specific gene markers (*KRT5*, *KRT10*, and *KRT15*) expression in various groups at day 7 (*n* = 3 each). Data reported as mean ± s.d. of replicated experiments, statistical significance considered at ^*^*p* < 0.05, ^**^*p* < 0.01, ^***^*p* < 0.001, and ^****^*p* < 0.0001 (One-way ANOVA test with Tukey’s HSD *post-hoc* analysis).

**
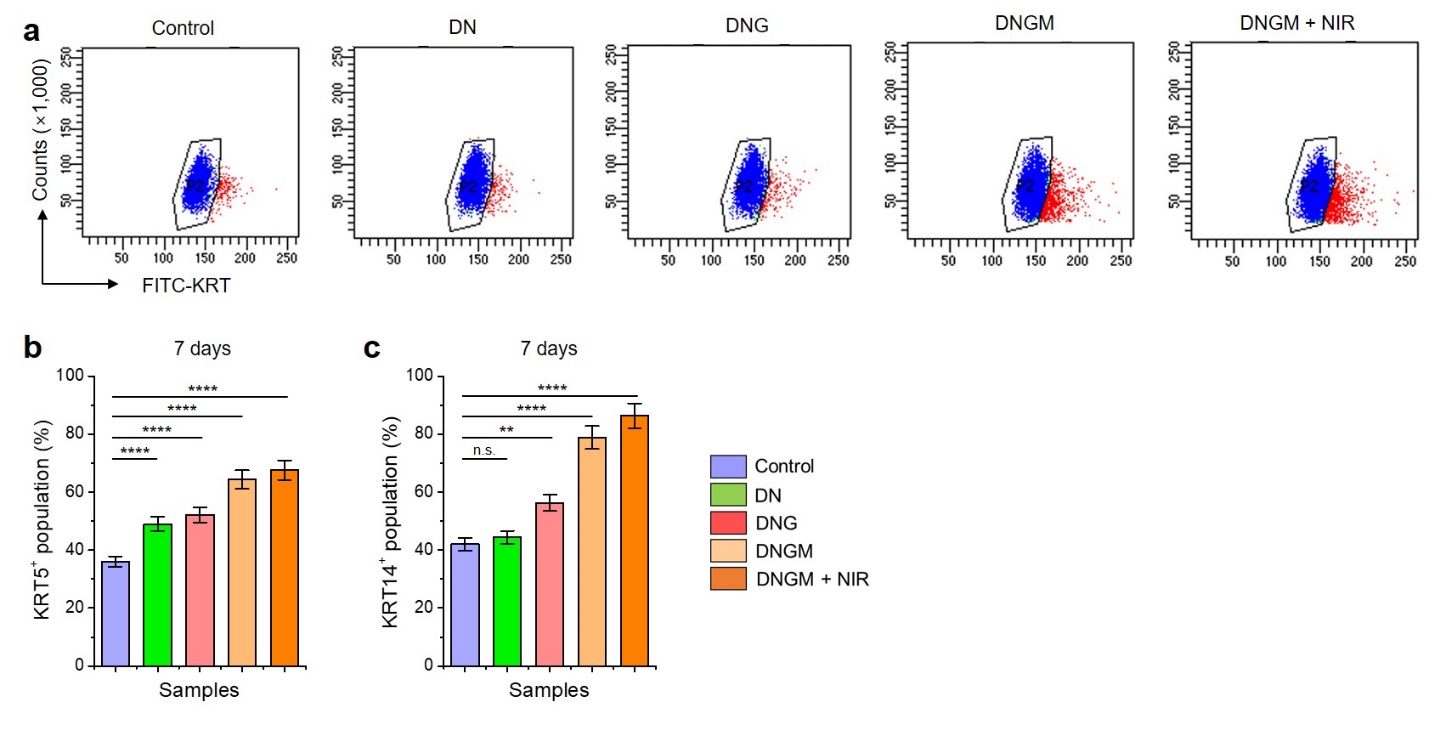
**

**Figure S18.** FACS analysis of the HaCaT cells showing the expression cytokeratin (KRT) markers after 7 days of culture with various formulations. **(a)** FACS acquisition data, and **(b, c)** KRT5^+^ and KRT14^+^ populations as of day 7 of *in vitro* culture. Data reported as mean ± s.d. of triplicated experiments, statistical significance considered at ^**^*p* < 0.01 and ^****^*p* < 0.0001 (One-way ANOVA followed by Tukey’s HSD *post-hoc* test).


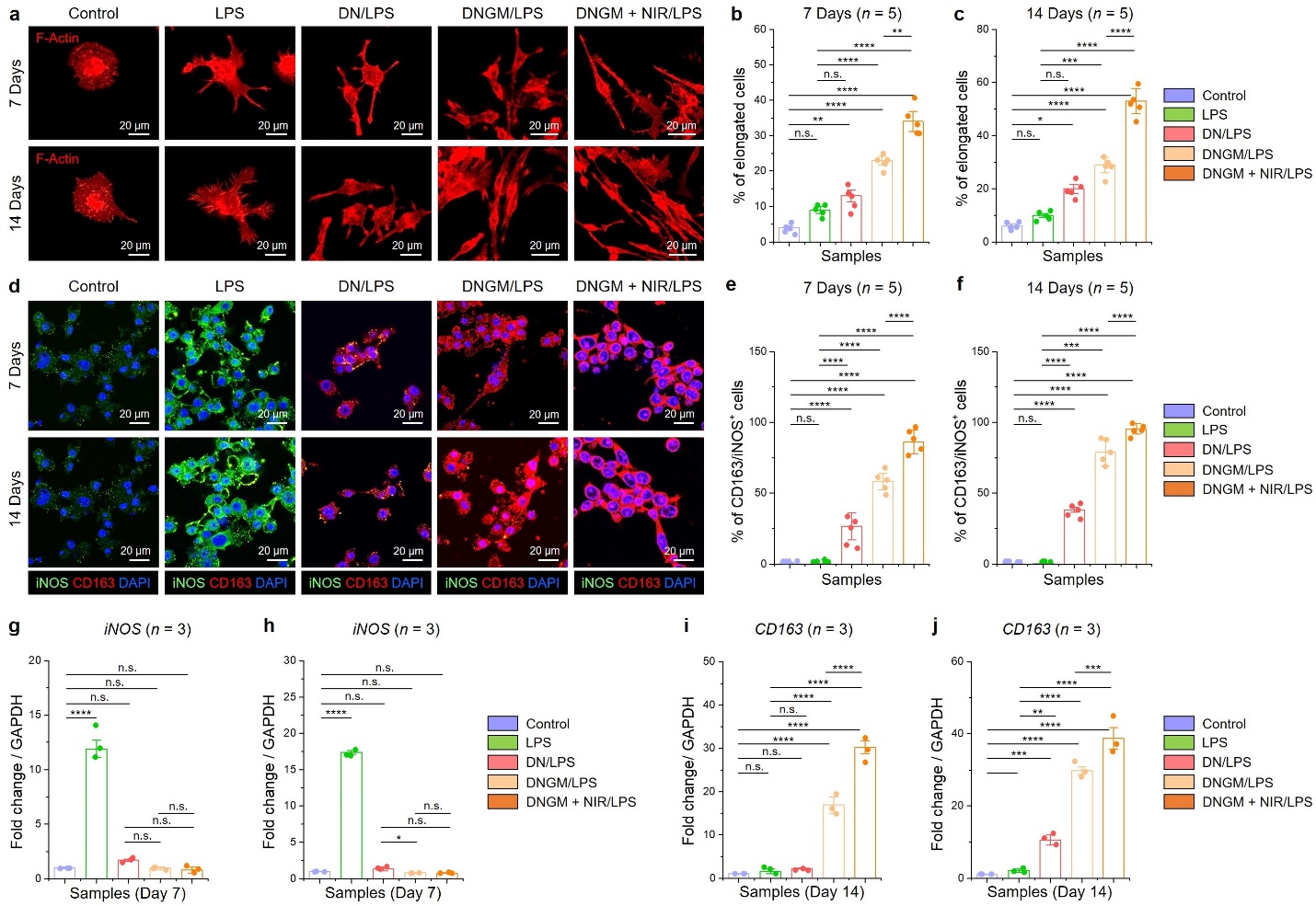


**Figure S19.** Long-term macrophage polarization study in the presence of the fabricated hydrogels. **(a)** Representative FL images of the RAW 264.7 cells showing the changes in morphology with various formulations at day 7 and day 14. The RAW 264.7 cells were stained with F-actin (red) probe at indicated time points. Scale bar: 20 µm. **(b, c)** Statistical analysis of the % of elongated cells (a key indication of M2-polarization) at day 7 and day 14 in various groups (*n* = 5 each). **(d)** Representative CLSM images of RAW 264.7 cells showing the expression of iNOS (green) and CD163 (red) at 7 and 14 days of culture with various formulations. Scale bar: 20 µm. **(e, f)** Statistical analysis of the % of CD163/iNOS^+^ cells at day 7 and day 14 (*n* = 5 each). Data reported as mean ± s.d. of replicated experiments, statistical significance considered at ^*^*p* < 0.05, ^**^*p* < 0.01, ^***^*p* < 0.001, and ^****^*p* < 0.0001 (One-way ANOVA test with Tukey’s HSD *post-hoc* analysis).

**
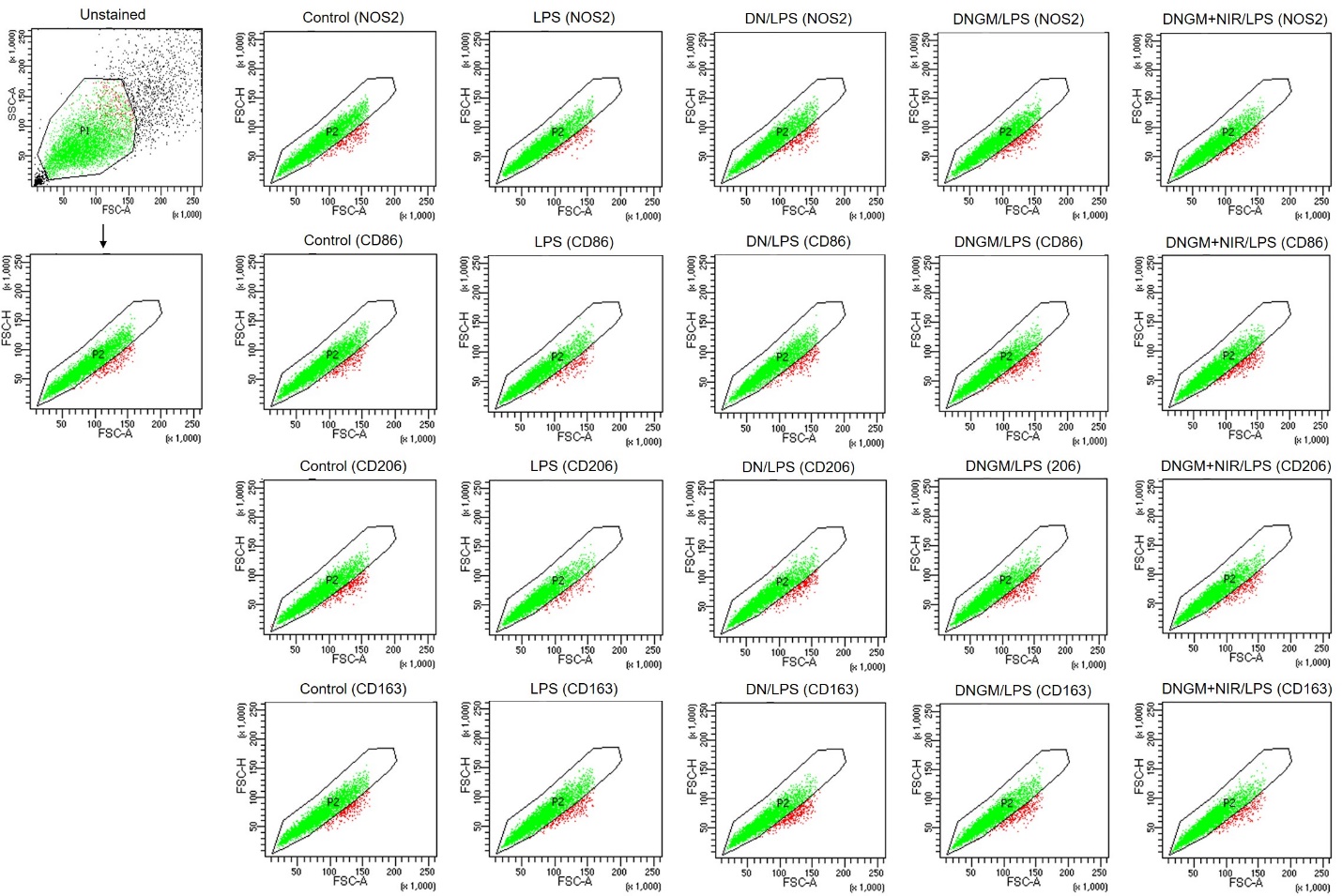
**

**Figure S20.** FACS gating strategies for macrophage polarization study. The RAW 264.7 cells were stained with FITC-labelled secondary antibodies and analyzed through FACS Symphony A3 (BD Bioscience, USA) and the data was processed using FACSDiva v9.3 (BD Bioscience, USA).

**
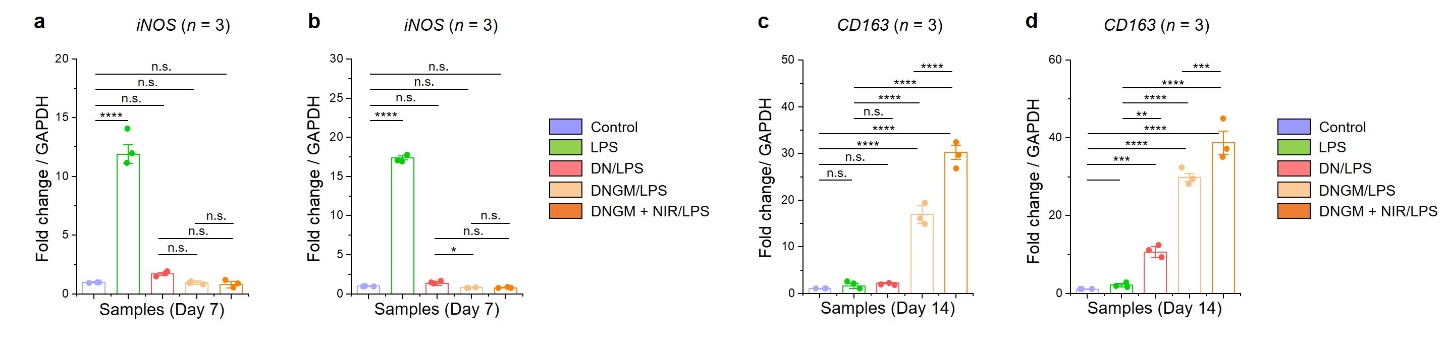
**

**Figure S21.** qRT-PCR validation of the canonical **(a, b)** M1 (*iNOS*) and **(c, d)** M2 (*CD163*) gene markers expression in RAW 264.7 cells at day 7 and day 14 in various groups (*n* = 3 each). Data reported as mean ± s.d. of replicated experiments, statistical significance considered at ^*^*p* < 0.05, ^**^*p* < 0.01, ^***^*p* < 0.001, and ^****^*p* < 0.0001 (One-way ANOVA test with Tukey’s HSD *post-hoc* analysis).

**
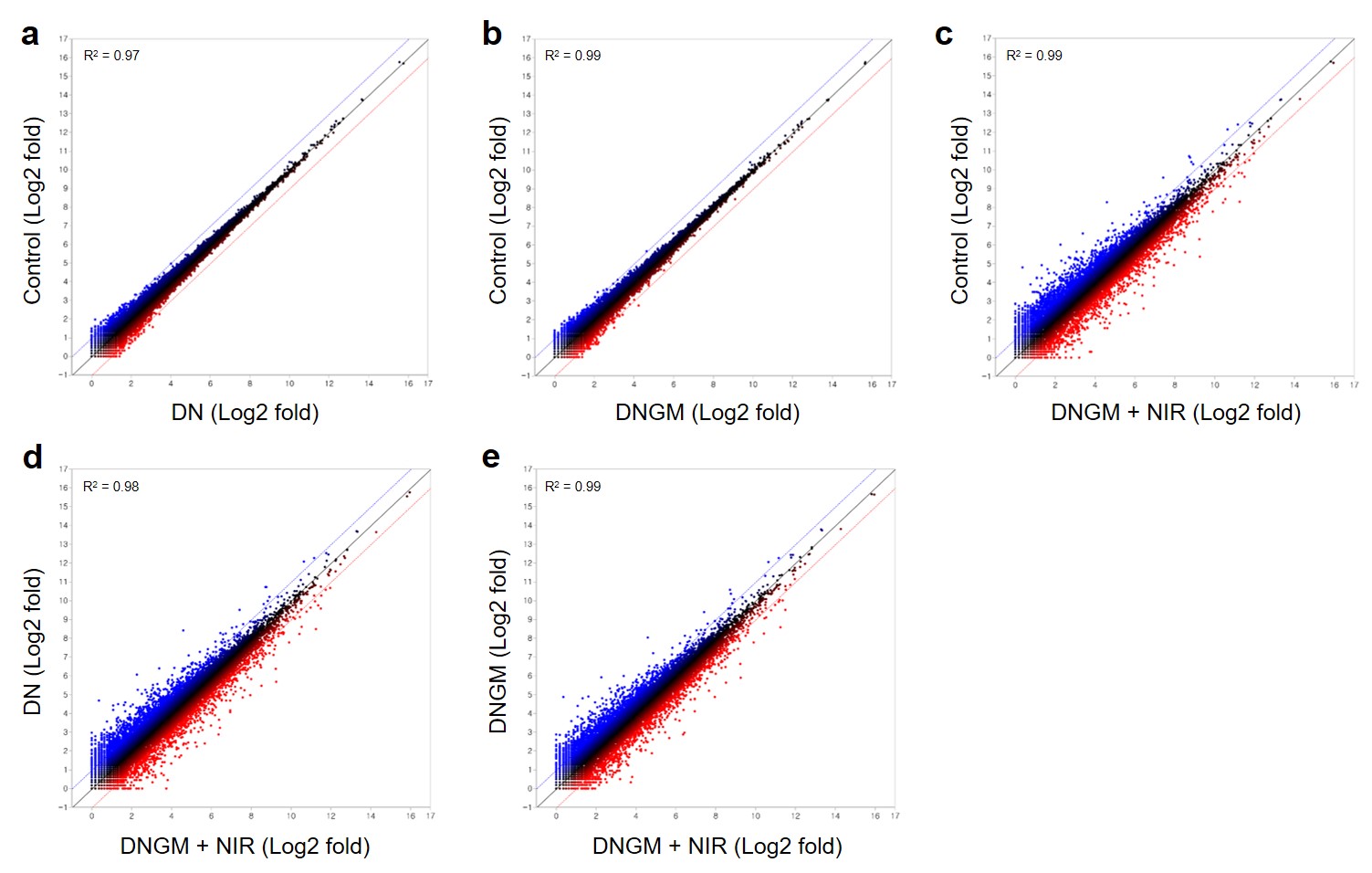
**

**Figure S22.** Scatter plots showing the log2-fold change (Log2FC) and R^2^ values of the differentially expressed genes (DEGs) when compared between **(a)** DN *vs*. Control, **(b)** DNGM *vs*. Control, **(c)** DNGM + NIR *vs*. Control, **(d)** DNGM + NIR *vs*. DN, and **(e)** DNGM + NIR *vs*. DNGM. The up-regulated genes are indicated with blue dots, while the down-regulated genes are indicated with red dots.


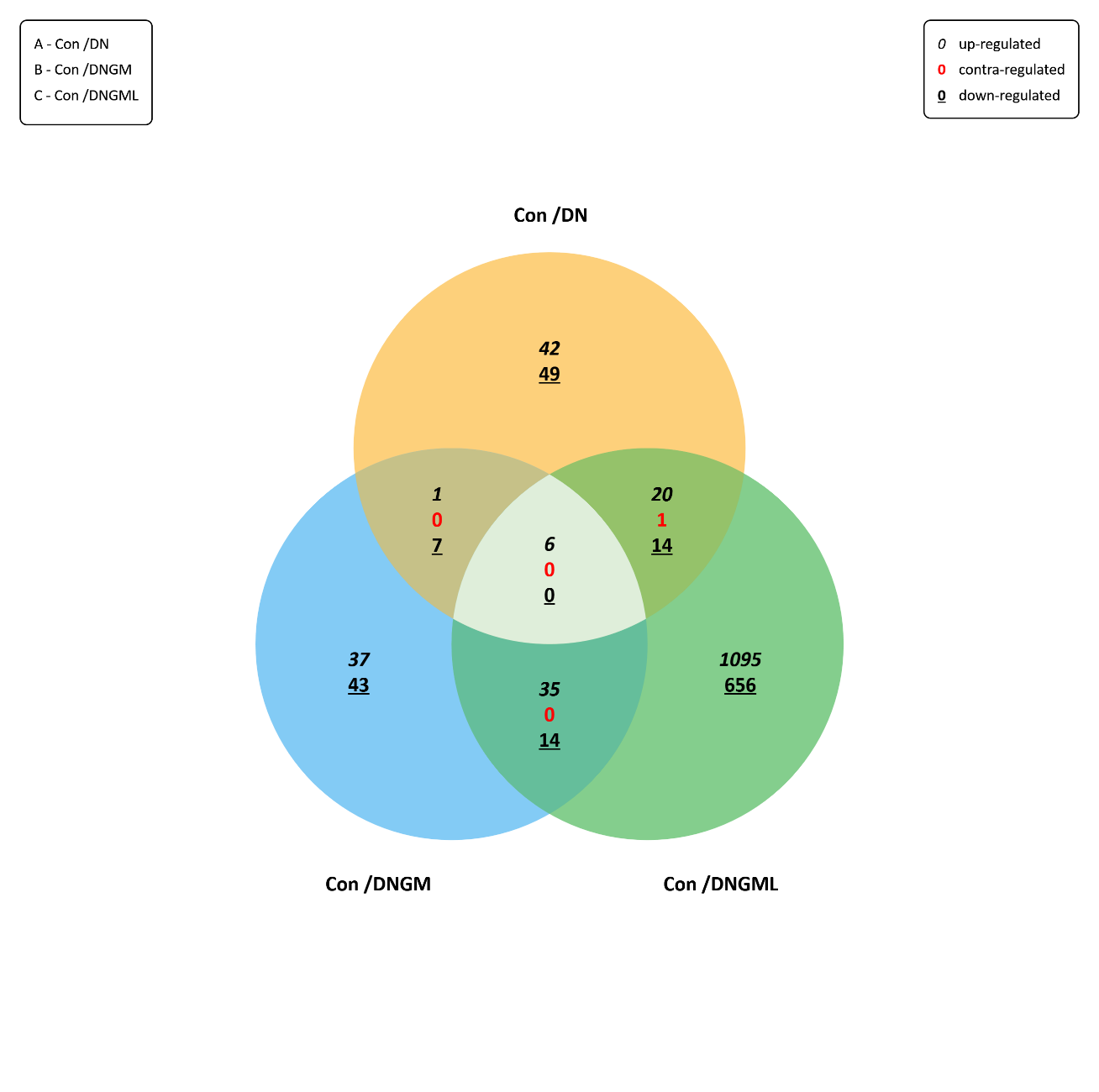


**Figure S23.** Venn diagram showing the number of significant DEGs overlapping across three different comparisons (Control/DN, Control/DNGM, and Control/DNGM + NIR) groups. The up-regulated DEGs are indicated in bold italics, down-regulated DEGs are indicated in underlined, and contra-regulated DEGs are indicated in red.

**
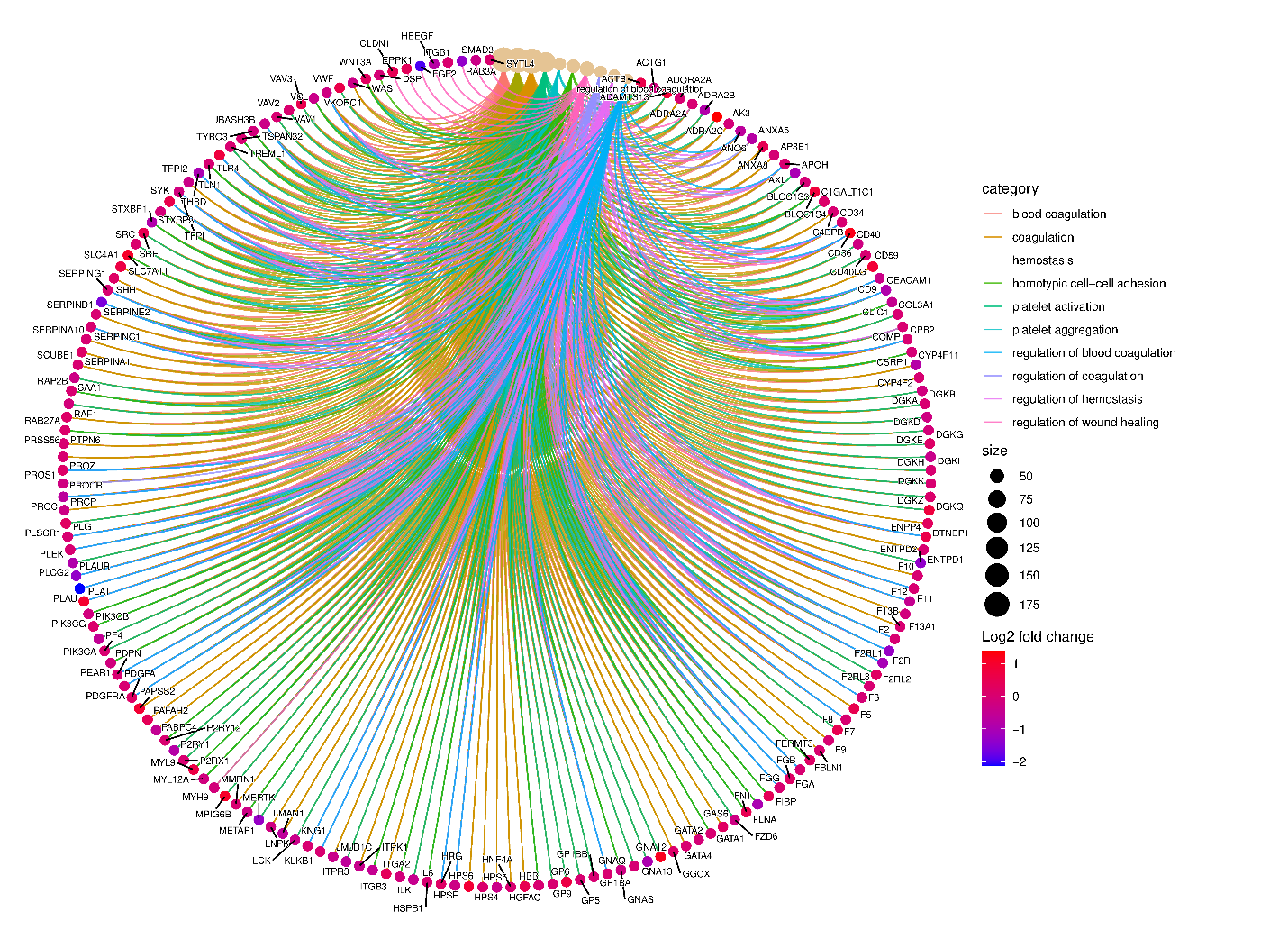
**

**Figure S24.** Cnet plot showing the enrichment in biological process (BP, KEGG_GO) and its associated DEGs (Log2FC) expression when compared between DNGM + NIR *vs*. Control group, as predicted through GO via clusterProfiler and g.Profiler.

**
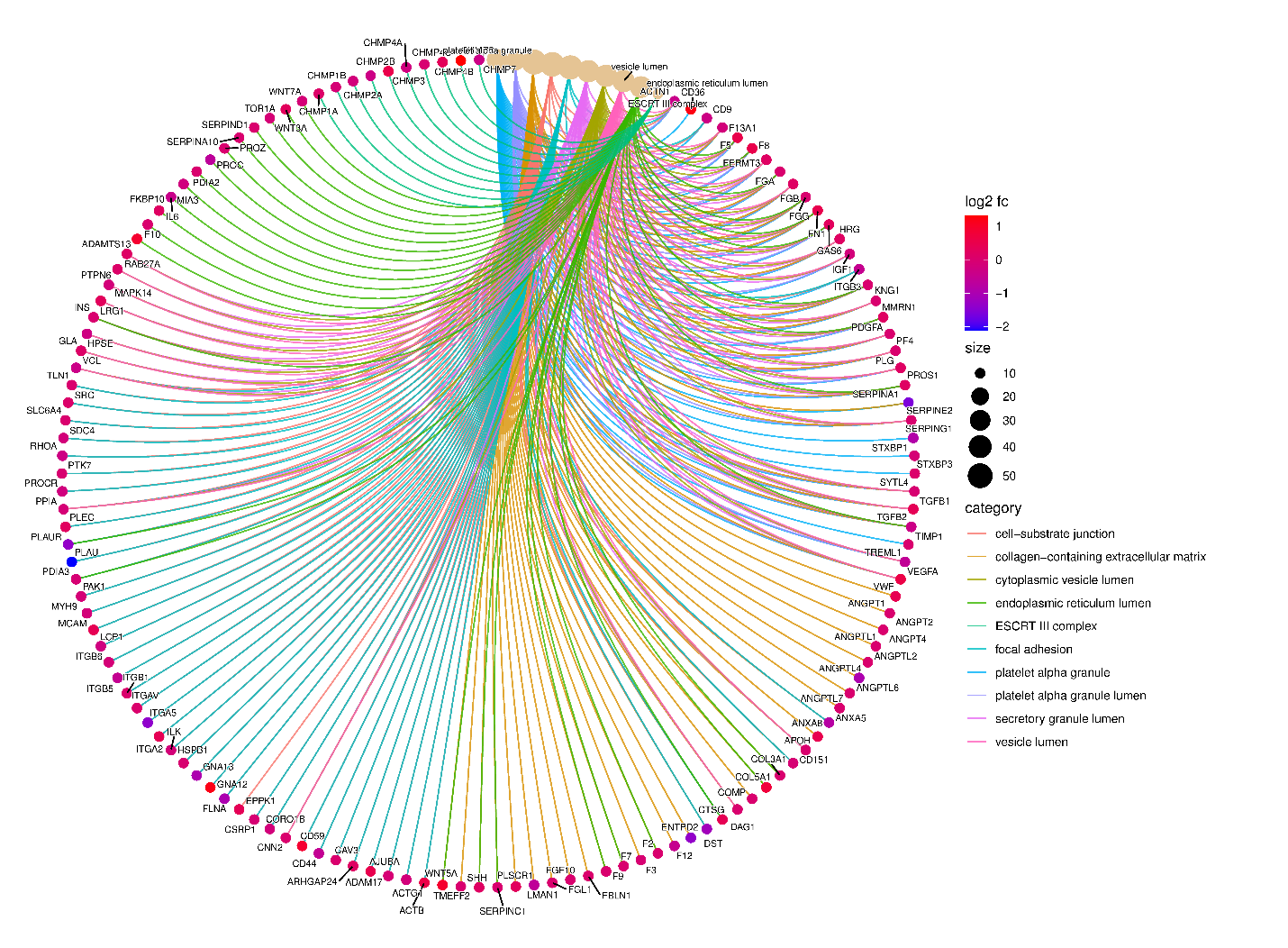
**

**Figure S25.** Cnet plot showing the enrichment in cellular component (CC, KEGG_GO) and its associated DEGs (Log2FC) expression when compared between DNGM + NIR *vs*. Control group, as predicted through GO via clusterProfiler and g.Profiler.

**
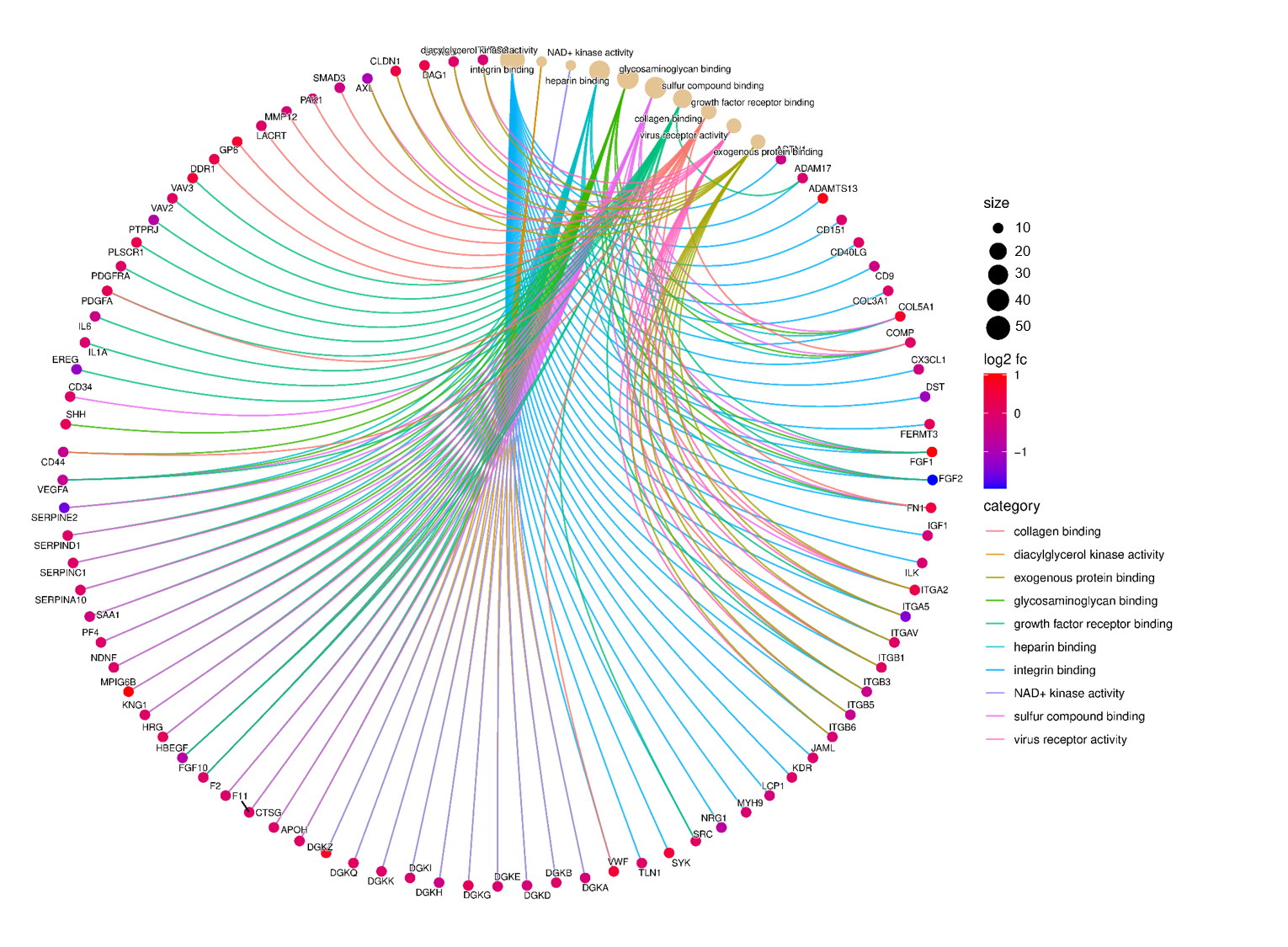
**

**Figure S26.** Cnet plot showing the enrichment in molecular function (MF, KEGG_GO) and its associated DEGs (Log2FC) expression when compared between DNGM + NIR *vs*. Control group, as predicted through GO via clusterProfiler and g.Profiler.

**
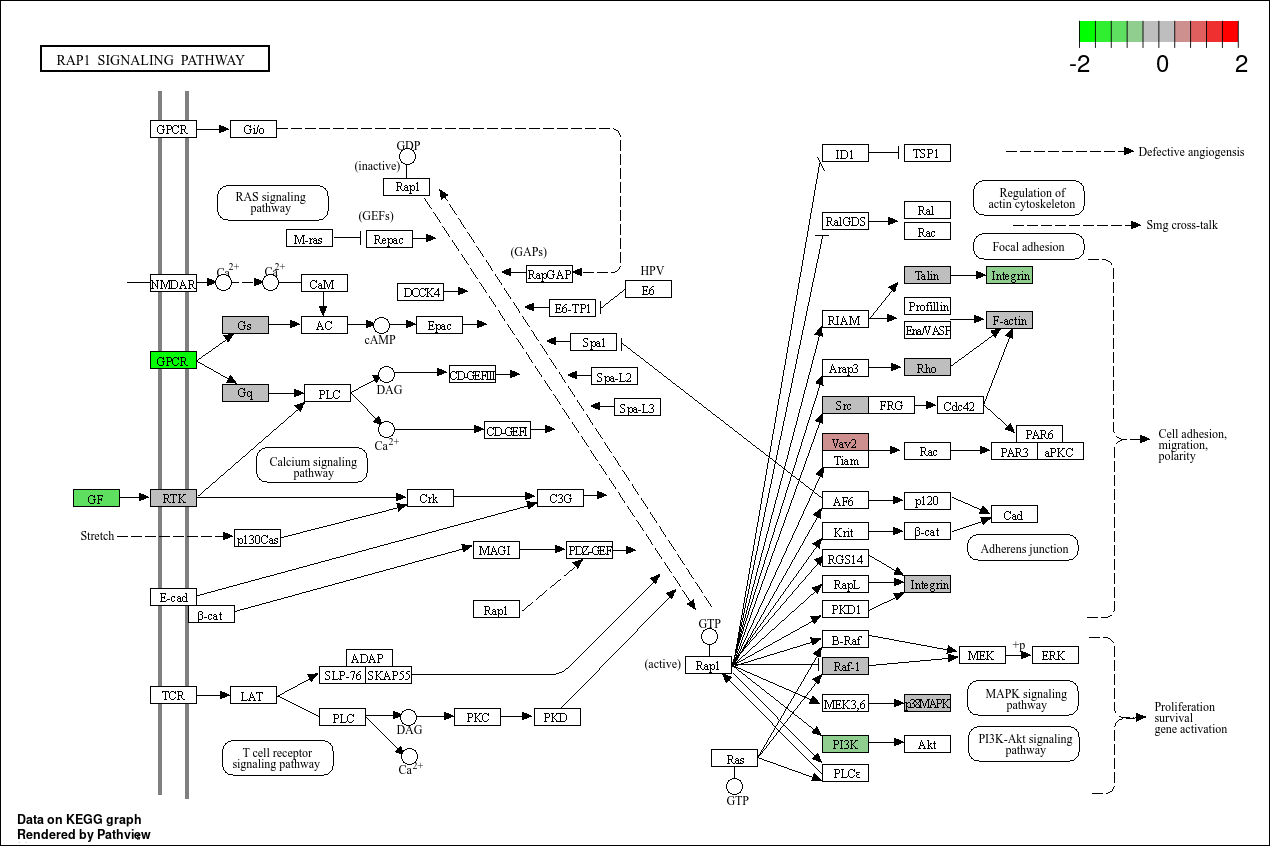
**

**Figure S27.** Representative KEGG pathway enrichment in ‘*RAPI signaling pathway*’ in DNGM + NIR *vs*. Control group after 7 days of treatment. The upregulated genes are represented in red, while the downregulated genes are represented in green color. KEGG pathway rendered by Pathview.

**
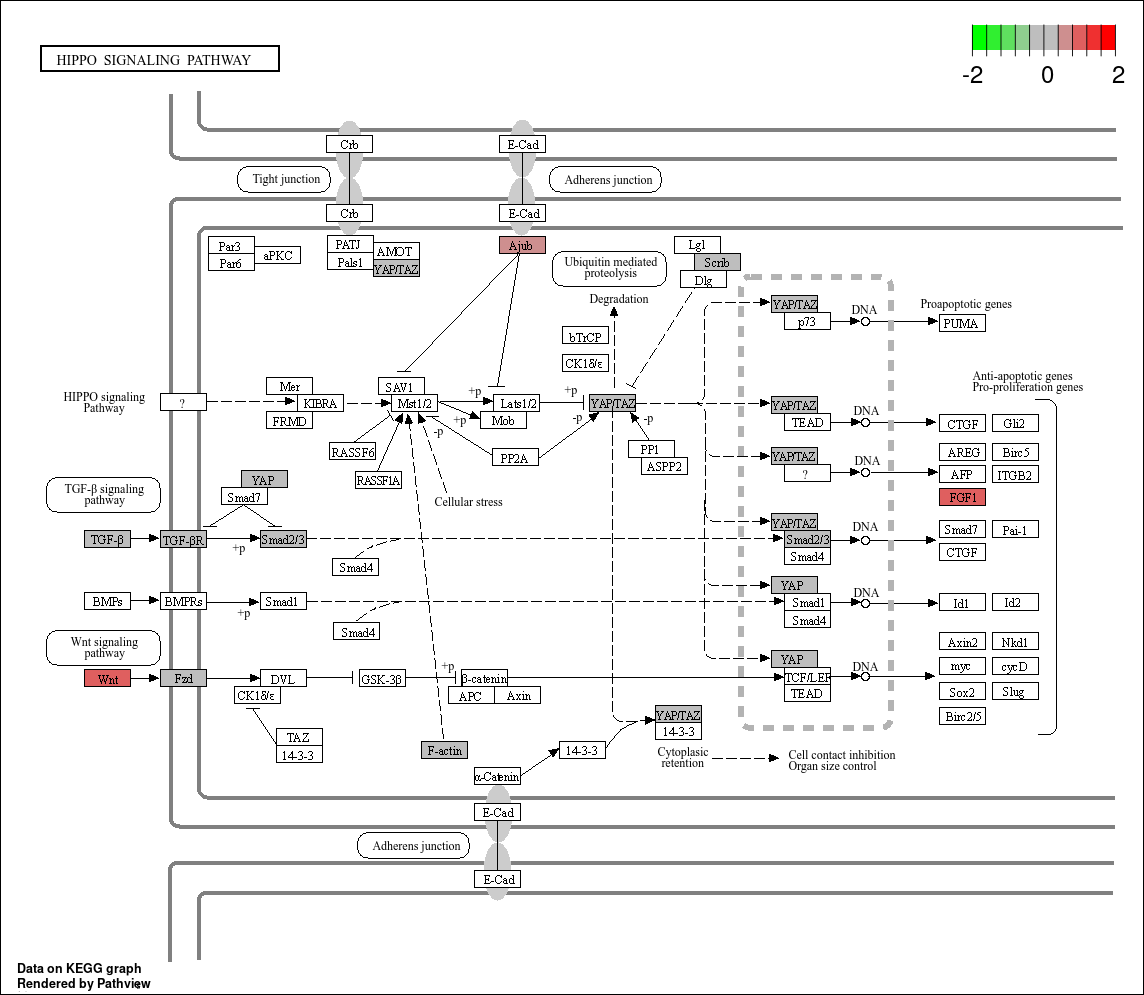
**

**Figure S28.** Representative KEGG pathway enrichment in ‘*HIPPO signaling pathway*’ in DNGM + NIR *vs*. Control group after 7 days of treatment. The upregulated genes are represented in red, while the downregulated genes are represented in green color. KEGG pathway rendered by Pathview.

**
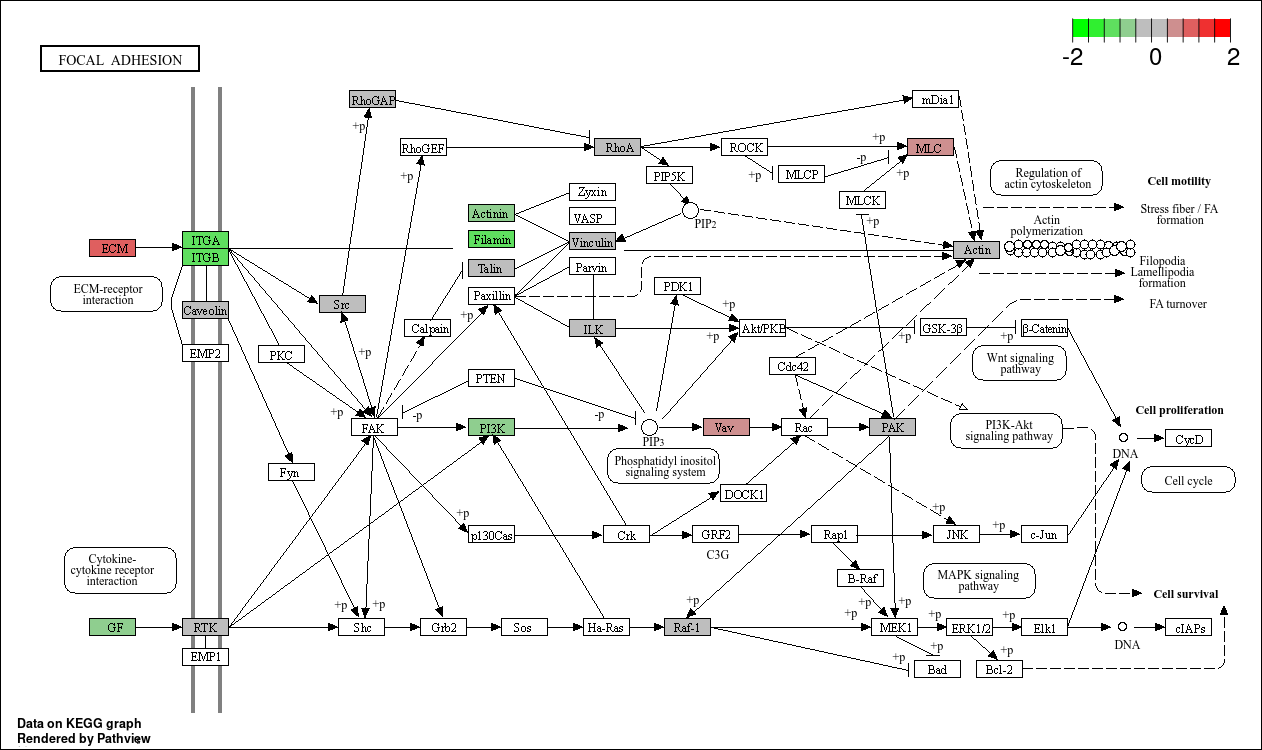
**

**Figure S29.** Representative KEGG pathway enrichment in ‘*Focal adhesion*’ in DNGM + NIR *vs*. Control group after 7 days of treatment. The upregulated genes are represented in red, while the downregulated genes are expressed in green. KEGG pathway rendered by Pathview.

**
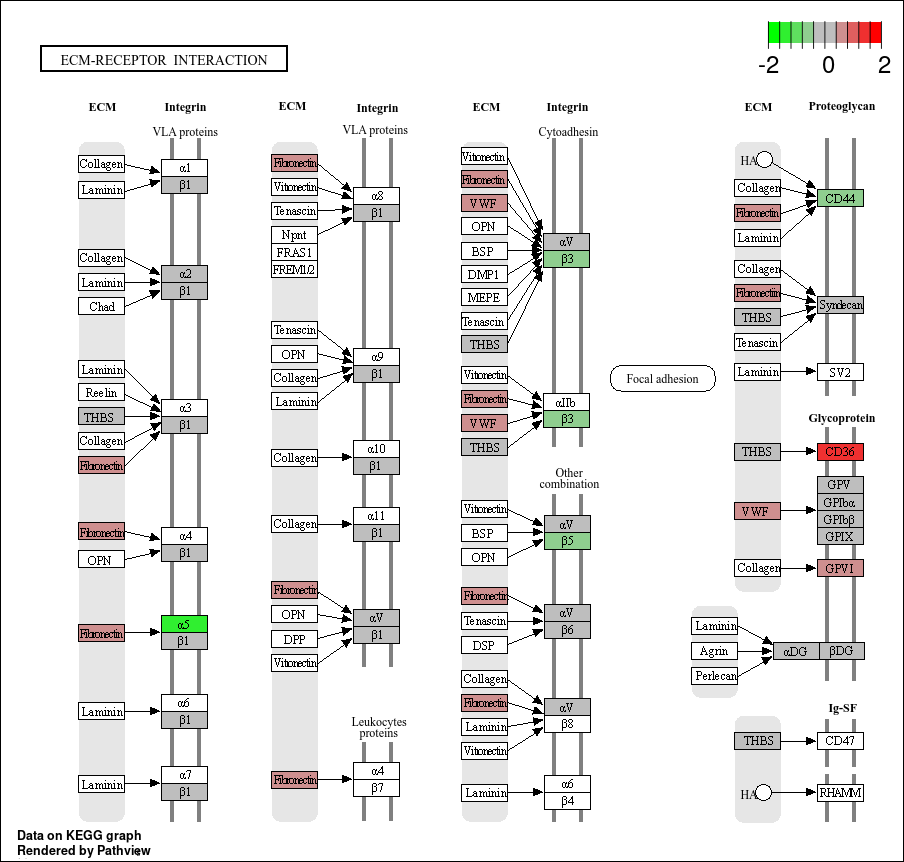
**

**Figure S30.** Representative KEGG pathway enrichment in ‘*ECM-receptor interaction*’ in DNGM + NIR *vs*. Control group after 7 days of treatment. The upregulated genes are represented in red, while the downregulated genes are expressed in green. KEGG pathway rendered by Pathview.

**
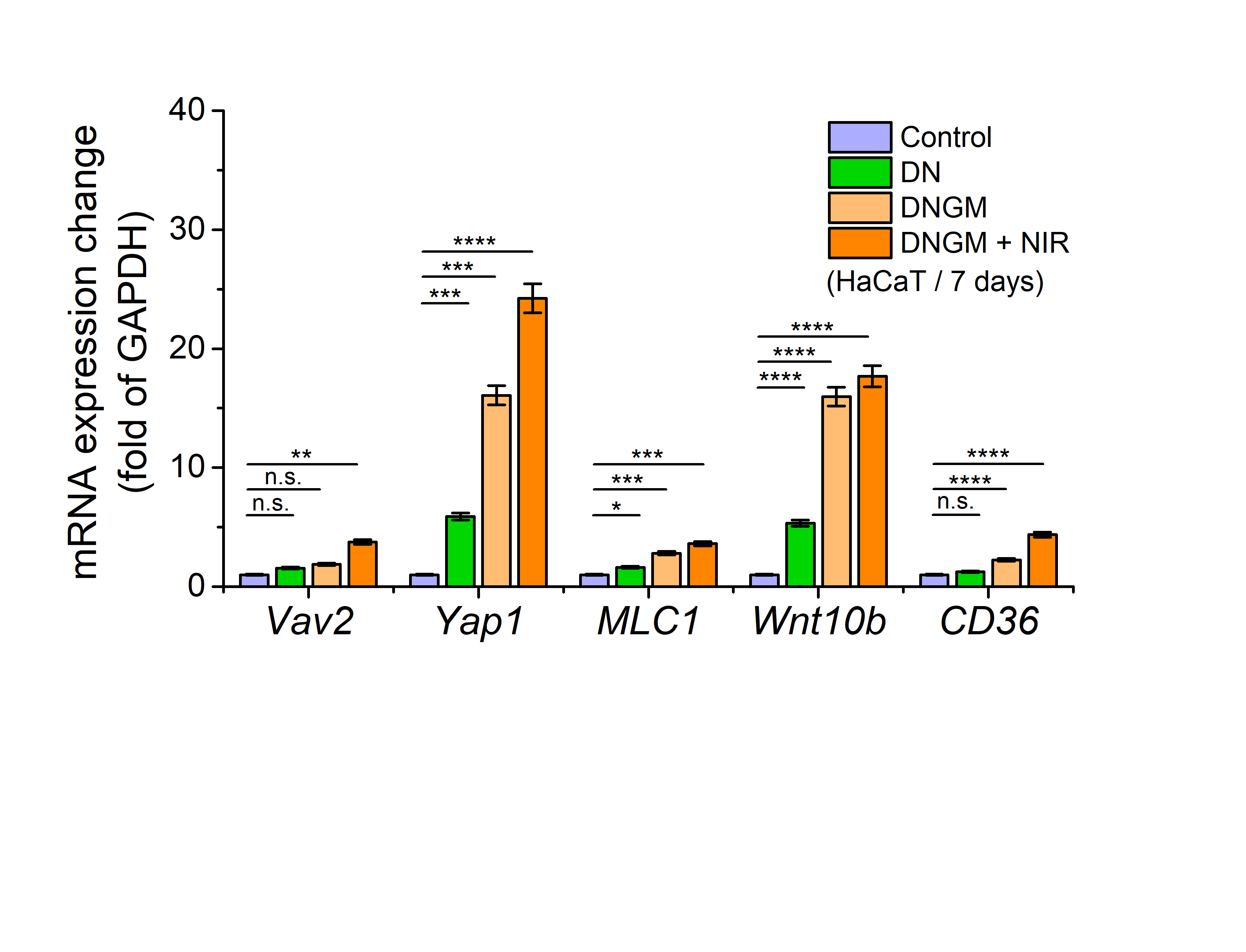
**

**Figure S31.** qRT-PCR validation of key gene markers expression associated with keratinization and wound healing in HaCaT cells as identified through bulk RNA-Seq analysis. Data reported as mean ± s.d. of triplicated (*n* = 3) experiments, statistical significance considered at ^*^*p* < 0.05, ^**^*p* < 0.01, ^***^*p* < 0.001, and ^****^*p* < 0.0001 (One-way ANOVA followed by Tukey’s HSD *post-hoc* test). The n.s. indicates no significant data. Acronyms: *Vav2*; Vav guanine nucleotide exchange factor-2, *Yap1*; Yes-associated protein-1, *MLC1*; Myosin light chain protein-I, *Wnt10b*; Wnt-like protein-10b, *CD36*; Cluster of differentiation-36.


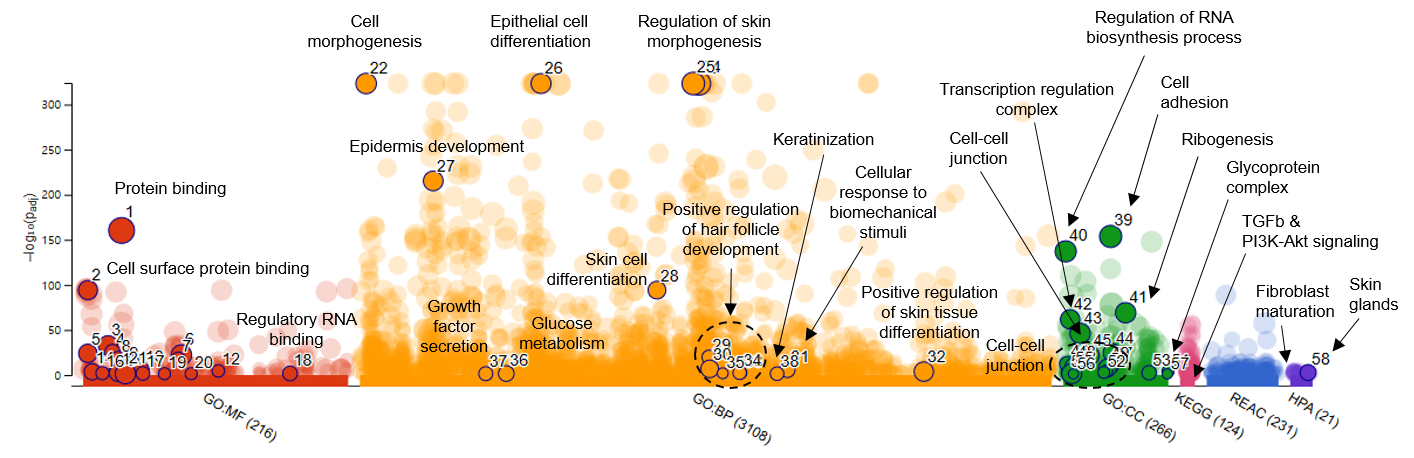


**Figure S32.** Comparison of gene sets in DNGM + NIR *vs*. Control group showing the statistically significantly (FDR < 0.05) enriched terms in gene ontology (GO: MF, CC, and BP), KEGG, Reactome (REAC), and human protein atlas (HPA) database rendered by g.Profiler.

**
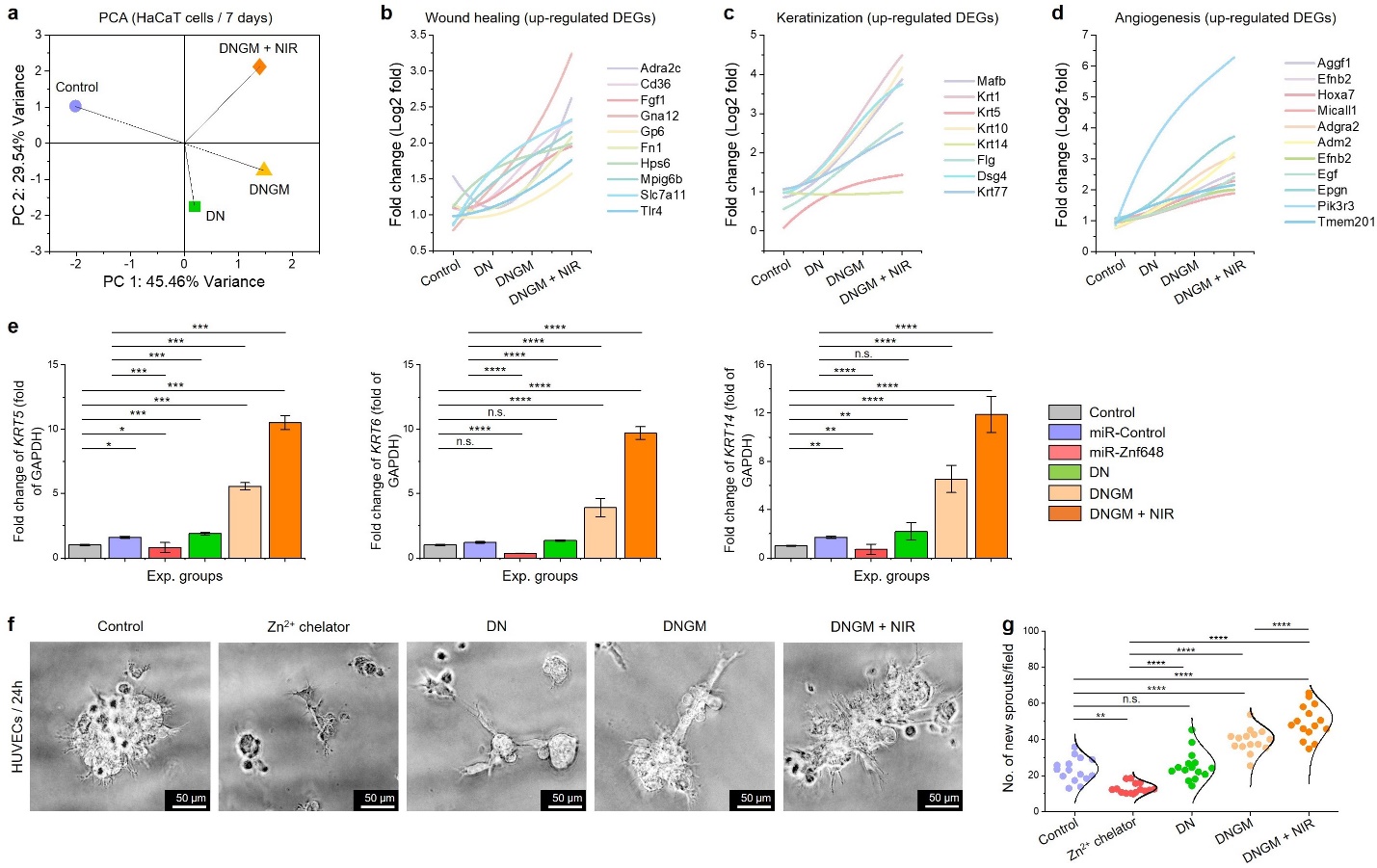
**

**Figure S33. (a)** Representative Principal Component Analysis (PCA) of top-up/down-regulated genes in ECM remodeling during the wound healing process. **(b-d)** Representative fold change (Log2 fold, ^*^*p* < 0.05) expression of key genes associated with wound healing, keratinization, and angiogenesis process in HaCaT cells after 7 days of treatment.

**
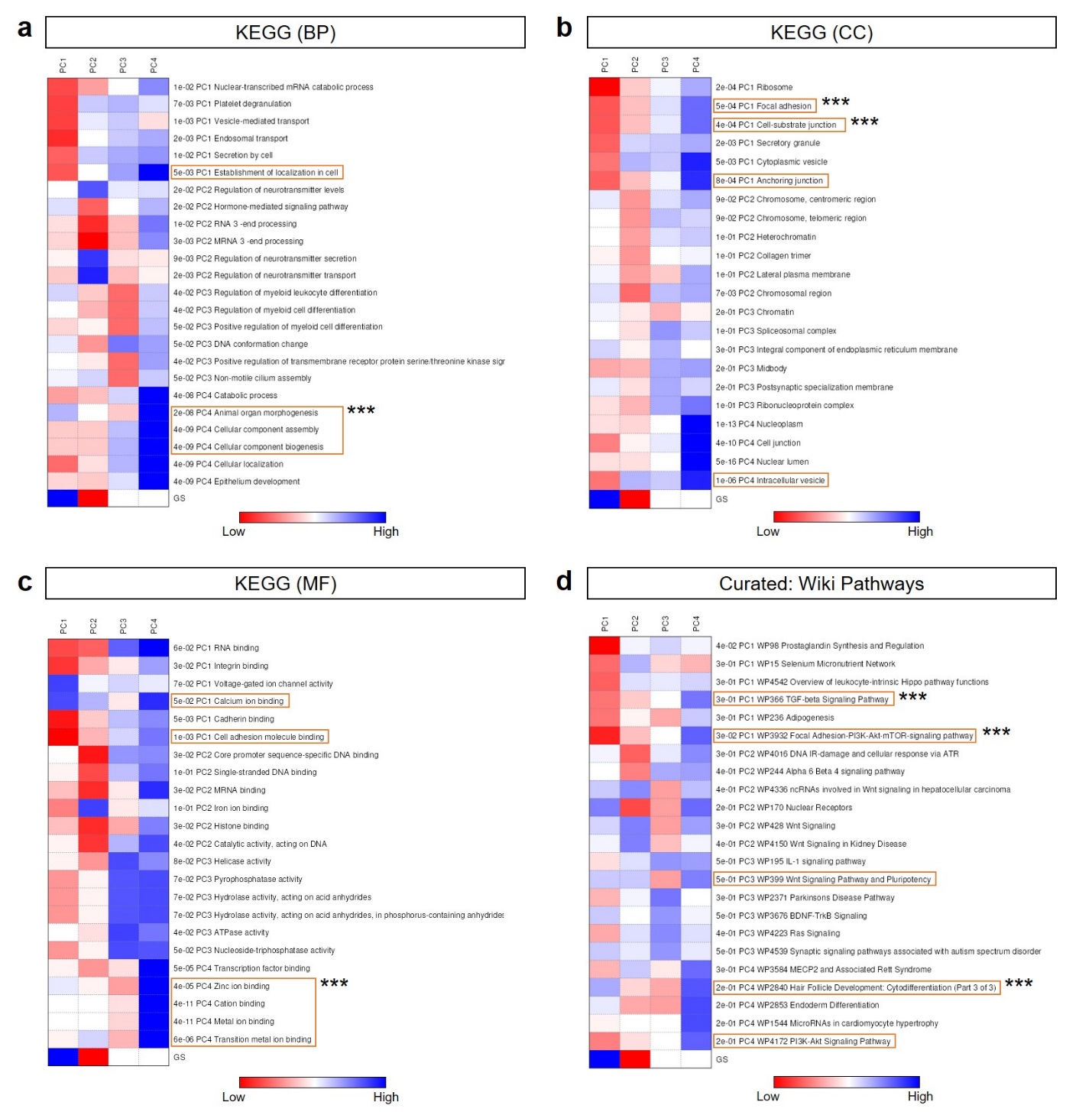
**

**Figure S34.** Multi-dimensional principle component analysis (PCA) of ECM remodeling related DEGs in DNGM + NIR *vs*. Control group showing **(a-c)** the KEGG and **(d)** Wiki pathway enrichment in HaCaT cells. Asterisks indicate the most significant terms associated with HaCaT cell remodeling when cultured with DNGM cryogel with NIR irradiation for 7 days.

**
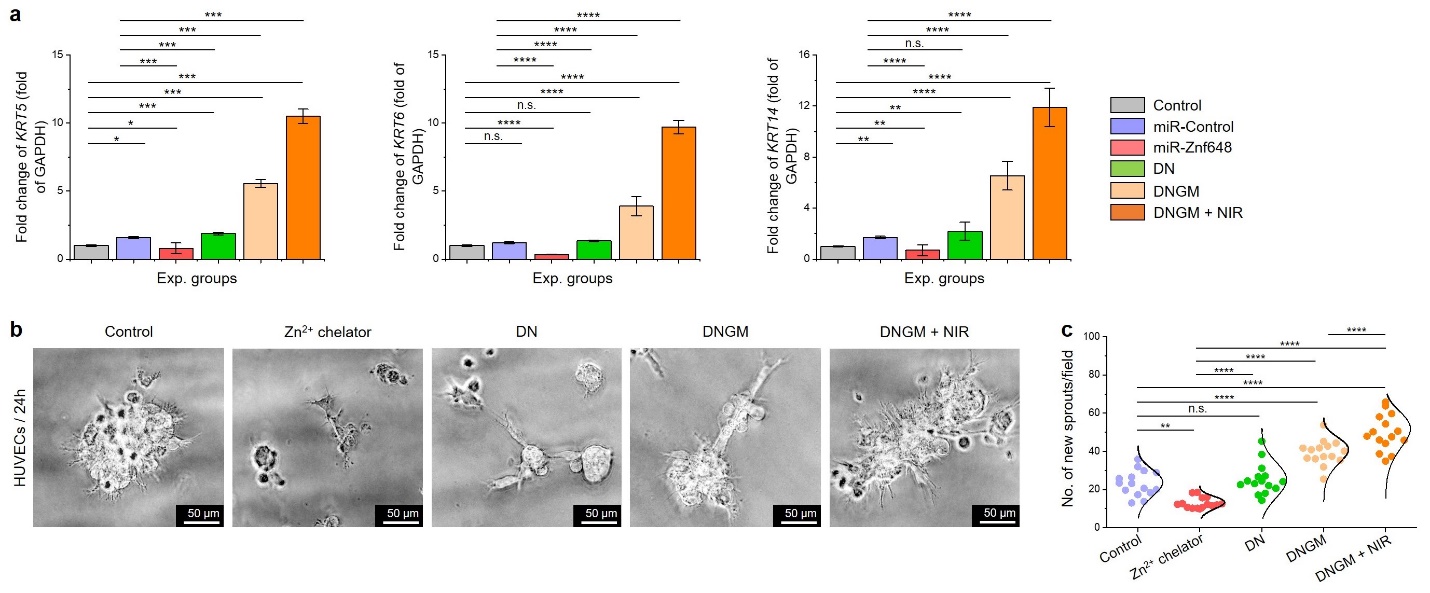
**

**Figure S35. (a)** qRT-PCR validation of RNA-Seq data for keratinization/wound healing gene markers expression in HaCaT cells at day 7. **(b, c)** Validation of RNA-Seq data for angiogenesis. Representative digital photographs of the 3D angiogenesis assay showing the effect of DNGM + NIR after 24 h of incubation. Matrigel was taken as control, while DETPA (Zn^2+^ chelator) was used as Zn^2+^ inhibitor to investigate the role of Zn^2+^ in angiogenesis. Scale bar: 50 µm. Data reported as mean ± s.d. of replicated experiments, statistical significance considered at ^*^*p* < 0.05, ^**^*p* < 0.01, ^***^*p* < 0.001, and ^****^*p* < 0.0001 (One-way ANOVA followed by Tukey’s HSD *post-hoc* test). The n.s. indicates no significant data.

**
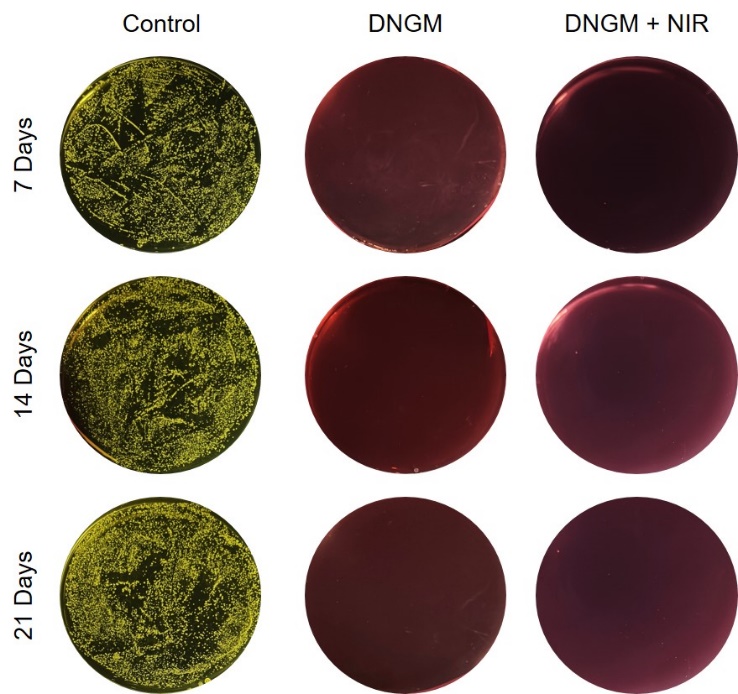
**

**Figure S36.** Representative digital photographs of the plate dilution assay of MRSA collected from the wound bed at day 7, day 14, and day 21 in various groups showing the excellent removal of bacterial burden.

**
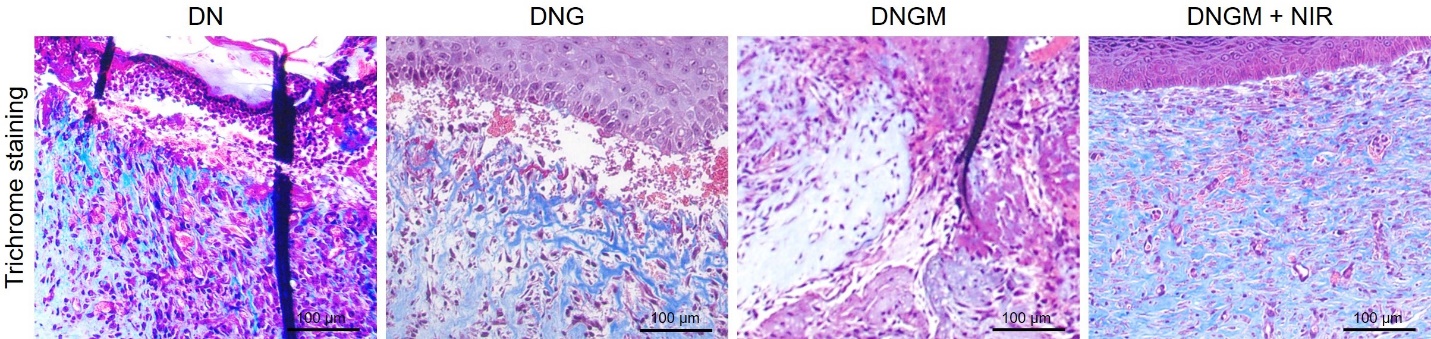
**

**Figure S37.** Magnified images of trichrome staining results from the wound bed showing the collagen alignment after 14 days of scaffold implantation in different groups. Scale bar: 100 µm.


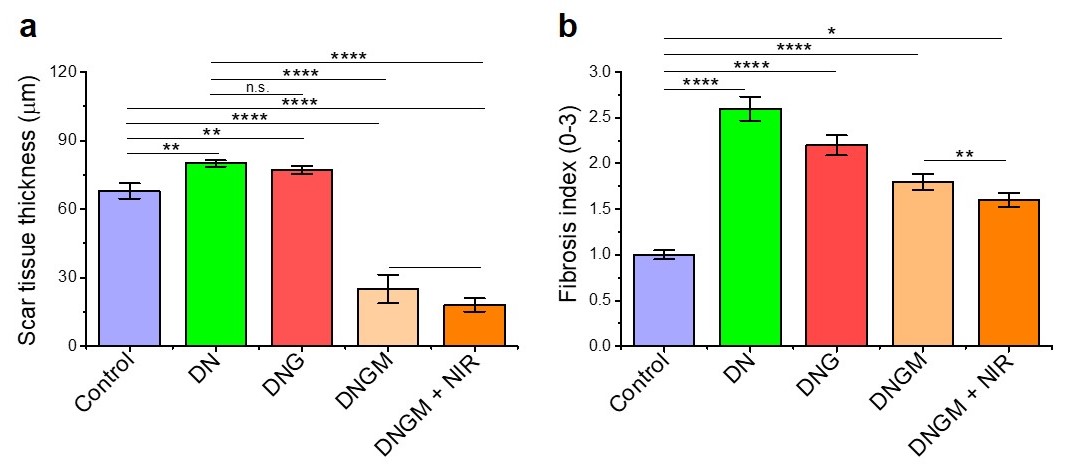


**Figure S38.** **(a)** Scar tissue thickness and **(b)** Fibrosis (scar formation) index of the wound bed tissue at day 14. Data are presented as mean ± s.d. from triplicate experiments (*n* = 3). Statistical significance is considered at ^*^*p* < 0.05, ^**^*p* < 0.01, and ^****^*p* < 0.0001 as determined by One-way ANOVA followed by Tukey’s HSD *post-hoc* test.

**
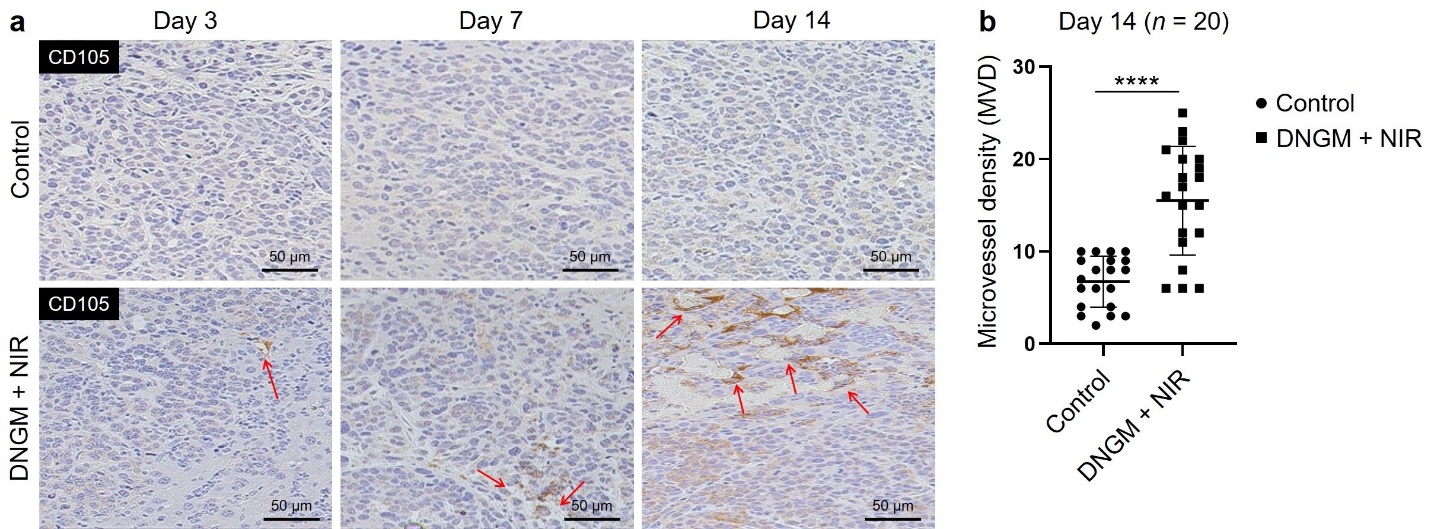
**

**Figure S39.** **(a, b)** Immunohistochemistry of wound tissue in control and DNGM + NIR groups showing CD105 (=endoglin) expression, indicating the presence of microvessel. Scale bar: 50 µm. Data are presented as mean ± s.d. from replicated experiments. Statistical significance is considered at ^****^*p* < 0.0001 as determined by One-way ANOVA with Tukey’s HSD *post-hoc* test.


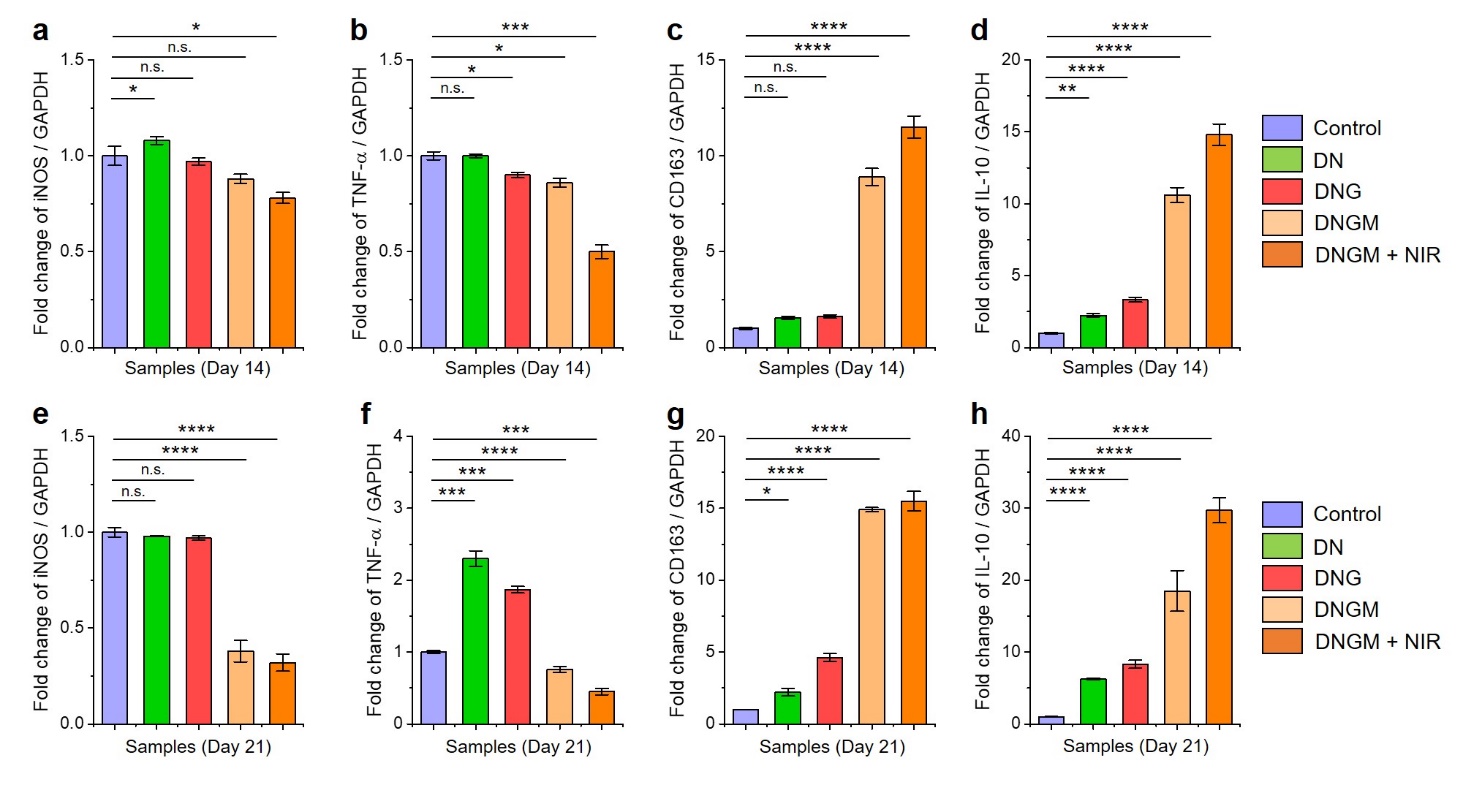


**Figure S40.** Representative qRT-PCR analysis of the wound tissue displaying the expression of genes related to the inflammatory response at days 14 and 21 in the wound bed. Data are reported as mean ± s.d. from triplicated experiments (*n* = 3); statistical significance is considered at ^*^*p* < 0.05, ^**^*p* < 0.01, ^***^*p* < 0.001, and ^****^*p* < 0.0001 (One-way ANOVA followed by Tukey’s HSD *post-hoc* test). Samples marked as *n.s.* indicate no significant data.

**Table S1.** A comparative study on various DNA-based hydrogels for wound healing.

| **Gel composition** | **Mechanical properties** | **Stimuli** | **G′ (Pa)** | **Antibacterial**  **performance** | **References** |
| --- | --- | --- | --- | --- | --- |
| dA_30_-dsDNA | N/A | N/A | 10^5^ | N/A | [[6](#_ENREF_6)] |
| Ac_4_GalNAz/DBCO-  cDNA | N/A | N/A | ~10 | N/A | [[7](#_ENREF_7)] |
| DNA/FKNa/Ag^+^ | N/A | N/A | 10^3^ | High (>90%) | [[8](#_ENREF_8)] |
| DNA/PEGDA | N/A | N/A | 10^3^ | N/A | [[9](#_ENREF_9)] |
| Y scaffold (L- and D-DNA) | N/A | N/A | 10^3^ | N/A | [[10](#_ENREF_10)] |
| tFNA/Apt02 | N/A | N/A | 10^-5^ | N/A | [[11](#_ENREF_11)] |
| phi29-DNAP | N/A | N/A | N/A | N/A | [[12](#_ENREF_12)] |
| Y1-L_4_/L_5_/IL-33 | N/A | N/A | ~10 | N/A | [[13](#_ENREF_13)] |
| 3D printed alginate  /SiO_2_/DNA | CS: 3.79 ± 0.9 kPa | N/A | 10^3^ | N/A | [[14](#_ENREF_14)] |
| Procyanidin B2/  DNA | N/A | N/A | 10^2^ | High (>90%) | [[15](#_ENREF_15)] |
| Polyacrylamide/DNA/  L-A2P | N/A | N/A | 10^3^ | High (>99%) | [[16](#_ENREF_16)] |
| 3D printed DNA@  PDA/acrylamide/  exosomes | EM: ~200 kPa | N/A | N/A | High (>90%) | [[17](#_ENREF_17)] |
| **DNA/MXene@ZIF8**  **/GelMA cryogel** | **CS: 16.9 ± 0.45 kPa**  **EM: 1.14 ± 0.61 kPa** | **NIR** | **10^4^** | **High (>99%)** | **This study** |

CS, compressive strength; EM, elastic modulus

**Table S2.** Description of the key protein identified from the HaCaT secretome after DNGM + NIR treatment at day 7.

| **Uniport ID** | **Name** | **Function (GO_BP)** | **Type** |
| --- | --- | --- | --- |
| P09341 | GRO-α | Extracellular region, CXCR receptor binding, growth factor activity | Chemokine |
| P10145 | IL-8 | Extracellular region, heparin binding, IL-8 signaling, angiogenesis, calcium ion signaling, cellular response to fibroblast growth factor stimulus | Chemokine |
| P03950 | ANG | Angiogenesis, cell differentiation, stress response | Cytokine |
| P01033 | TIMP-1 | Cellular Zn^2+^ binder, growth factor binding and inhibitor | Cytokine |
| O00300 | OPG | Extracellular matrix organization, response to xenobiotic stress, regulation of odontogenesis, RANKL/TNFSF11 signaling | Cytokine |
| P01137 | TGF-β1 | Extracellular region, collagen maturation, growth factor activity, cell morphogenesis, cellular response to mechanical stimuli, wound healing | Cytokine |
| P01133 | EGF | Extracellular region, angiogenesis, epithelial cell proliferation, ERK1/2 cascade, positive regulation of epithelial cell migration, receptor signaling via JAK/STAT signaling, positive regulation of Wnt signaling pathway | Cytokine |
| P80075 | MCP-2 | Extracellular region, protein kinase activity, cell-cell signaling, inflammatory response, positive regulation of cell migration | Chemokine |
| P22692 | IGFBP-4 | Extracellular space, IGF binding, MAPK cascade, regulation of cell growth, regulation of glucose metabolism process | Cytokine |

**Table S3.** Quantitative score of skin irritant (contact allergy) toxicity test.

| **Time (h)** | **Samples** | **Erythema** | **Edema** | **Positive ratio** |
| --- | --- | --- | --- | --- |
| 24 h | DN | 0 | 0 | 0 |
|  | DNG | 0 | 0 | 0 |
|  | DNGM | 0 | 0 | 0 |
|  | CDNB (Pos. Control) | 5 | 10 | 100 |
| 48 h | DN | 0 | 0 | 0 |
|  | DNG | 0 | 0 | 0 |
|  | DNGM | 0 | 0 | 0 |
|  | CDNB (Pos. Control) | 5 | 10 | 100 |
| 72 h | DN | 0 | 0 | 0 |
|  | DNG | 0 | 0 | 0 |
|  | DNGM | 0 | 0 | 0 |
|  | CDNB (Pos. Control) | 5 | 10 | 100 |

**Table S4.** List of antibodies used for ICC analysis.

| **Name** | **Dilution** | **Catalogue No.** | **Host species** | **Company** |
| --- | --- | --- | --- | --- |
| KRT1 (P) | 1:500 | sc-376224 | Mouse | Santa Cruz Biotech, USA |
| iNOS (P) | 1:500 | sc-7271 | Mouse | Santa Cruz Biotech, USA |
| CD163 (P) | 1:500 | sc-58965 | Mouse | Santa Cruz Biotech, USA |
| KRT5 (P) | 1:500 | sc-32328 | Mouse | Santa Cruz Biotech, USA |
| KRT10 (P) | 1:500 | sc-51581 | Mouse | Santa Cruz Biotech, USA |
| KRT14 (P) | 1:500 | sc-23878 | Mouse | Santa Cruz Biotech, USA |
| FBN (P) | 1:500 | sc-81769 | Mouse | Santa Cruz Biotech, USA |
| COL1A (P) | 1:500 | sc-59772 | Mouse | Santa Cruz Biotech, USA |
| DES (P) | 1:500 | sc-365130 | Mouse | Santa Cruz Biotech, USA |
| VIM (P) | 1:500 | sc-58899 | Mouse | Santa Cruz Biotech, USA |
| Anti α-SMA (P) | 1:500 | ab-5694 | Rabbit | Abcam, USA |
| Anti IgG H&L AF^®^-488 (S) | 1:500 | ab-150077 | Rabbit | Abcam, USA |
| m-IgGk BP-  FITC (S) | 1:250 | sc-516140 | Mouse | Santa Cruz Biotech, USA |

(P) primary antibody and (S) secondary antibody

**Table S5.** List of gene primers used for qRT-PCR analysis of HaCaT cells.

| **Genes** | **Forward sequence (5′**$\boldsymbol{\to}$**3′)** | **Reverse sequence (5′**$\boldsymbol{\to}$**3′)** |
| --- | --- | --- |
| *Fbn* | GCCATGACAATGGTGTGAAC | GCAAATGGCACCGAGATATT |
| *Col1A* | CTGACCTTCCTGCGCCTGATGTCC | GTCTGGGGCACCAACGTCCAAGGG |
| *Krt5* | AGAGTGGACCAACTGAAGAGT | ATTCTCTGCATTTGTCCGCTT |
| *Krt10* | TGGTGCCGGTAGTGGATTTG | CAGGAGGGCAGACAGGAAAG |
| *Krt14* | GATGATTGGCAGCGTGGAG | CAGAGGAGAACTGGGAGGAG |
| *Wnt10b* | GAGCCTACTTGCCCTTGATG | AACACTTGCTCCACAACCTC |
| *Mmp9* | TGAACATCTTCGACGCCATC | ACTTCCTGAGAAAGCGAGGA |
| *Timp1* | TGCTGTTCCCTAATCCCACT | AAGGACAGGGAAAAACTGGC |
| *Vav2* | GAGGAGATTCACGCACCTTC | CGTTCCCAGTCTGCTAATGG |
| *Yap1* | TTATTTTCCTCGCGGCTCAG | CCAACACGCGACCTACTAAG |
| *MLC1* | AGACAGGGCTGATGTCAGAA | CAACTGTGGGCTGACTTTCA |
| *CD36* | TGGGCAGAAGCGTGATATTG | GCGTTAGCTGTTAAGGAGGG |
| *CD31* | GGCTCAGACATCCACATAACC | CTTACCAGGGCGTTCAGGGAC |
| *GAPDH* | ACCACAGTCCATGCCATCA | TCCACCACCCTGTTGCTGT |

**Table S6.** List of gene primers used for qRT-PCR analysis of murine cells/tissue.

| **Genes** | **Forward sequence (5′**$\boldsymbol{\to}$**3′)** | **Reverse sequence (5′**$\boldsymbol{\to}$**3′)** |
| --- | --- | --- |
| *CD163* | GTGGTCAACTCCGCTTGGTA | CTTGGGGCACCATCTGTGAT |
| *iNOS* | GCACATCAAAGCGGCCATAG | CGGCAAACATGACTTCAGGC |
| *Arg-1* | AACACGGCAGTGGCTTTAAC | GTCAGTCCCTGGCTTATGGTT |
| *TNF-α* | CCCTCACACTCAGATCATCTTCT | GCTACGACGTGGGCTACAG |
| *IL-10* | GCTCTTACTGACTGGCATGAG | CGCAGCTCTAGGAGCATGTG |
| *Krt5* | AAGGTCAGCCCAAAGACAAC | CACGCAAATGGCTGAATGTC |
| *Krt10* | CTGTCATCACCTCTTCAGCG | GCCAATAATGAGAAGGCCGT |
| *Krt14* | GGTTCTTCTCCGCCATCTTC | TTAAGCAACCAATGGGCACA |
| *Wnt10b* | TCCAAGGGTGAAGCTGTTTG | AGAAGCTTCAAACACACCCC |
| *Mmp9* | GCAGAGTGAGTAGGCCAATG | TCAGGTTTTTGCTTCCTGGG |
| *Timp1* | CAGCTCCAGGGGATTTGATG | ACTGACATCCTTGGGCCTAA |
| *GAPDH* | CATACAGGTTTCTCCAGGCG | TTGTGATGGGTGTGAACCAC |

**Supplementary References:**

[1] M. Moniruzzaman, C. K. Maity, S. De, M. J. Kim, J. Kim, *ACS Applied Nano Materials* 2024, 7, 6636.

[2] S. D. Dutta, K. Ganguly, A. Randhawa, T. Patil, D. K. Patel, K.-T. Lim, *Biomaterials* 2023, 121999.

[3] M. Moniruzzaman, S. D. Dutta, K.-T. Lim, J. Kim, *ACS omega* 2022, 7, 37388.

[4] A. Randhawa, K. Ganguly, S. D. Dutta, T. V. Patil, K.-T. Lim, *Biomaterials* 2025, 312, 122713.

[5] S. D. Dutta, J. M. An, J. Hexiu, A. Randhawa, K. Ganguly, T. V. Patil, T. Thambi, J. Kim, Y.-k. Lee, K.-T. Lim, *Bioactive Materials* 2025, 45, 345.

[6] C. Lachance‐Brais, M. Rammal, J. Asohan, A. Katolik, X. Luo, D. Saliba, A. Jonderian, M. J. Damha, M. J. Harrington, H. F. Sleiman, *Advanced Science* 2023, 10, 2205713.

[7] H. Nam, H. Jeon, H. Kim, H. Y. Yoon, S. H. Kim, J. B. Lee, *Chemical Engineering Journal* 2023, 452, 139492.

[8] F. Gao, X. Ma, F. Wang, F. Zhou, J. Ye, D. Yang, M. Li, P. Wang, *Chemical Engineering Journal* 2023, 470, 144347.

[9] R. Ye, Z. Zhu, T. Gu, D. Cao, K. Jiang, Q. Dai, K. Xing, Y. Jiang, S. Zhou, P. Cai, *Nature Communications* 2024, 15, 5557.

[10] B. Zhou, B. Yang, Q. Liu, L. Jin, Y. Shao, T. Yuan, Y.-n. Zhang, C. Wang, Z. Shi, X. Li, *Journal of the American Chemical Society* 2023, 145, 8954.

[11] Y. Han, Y. Wu, F. Wang, G. Li, J. Wang, X. Wu, A. Deng, X. Ren, X. Wang, J. Gao, *Bioactive Materials* 2024, 35, 1.

[12] P. Gaspers, P. Lemke, A. Delavault, C. M. Domínguez, K. S. Rabe, C. M. Niemeyer, *Chemistry–A European Journal* 2024, 30, e202403047.

[13] Z. Wang, W. Li, L. Gou, Y. Zhou, G. Peng, J. Zhang, J. Liu, R. Li, H. Ni, W. Zhang, *Advanced Healthcare Materials* 2022, 11, 2200782.

[14] N. Kim, H. Lee, G. Han, M. Kang, S. Park, D. E. Kim, M. Lee, M. J. Kim, Y. Na, S. Oh, *Advanced Science* 2023, 10, 2300816.

[15] L. Zhou, W. Pi, S. Cheng, Z. Gu, K. Zhang, T. Min, W. Zhang, H. Du, P. Zhang, Y. Wen, *Advanced Functional Materials* 2021, 31, 2106167.

[16] L. Zhou, Z. Zeng, S. Liu, T. Min, W. Zhang, X. Bian, H. Du, P. Zhang, Y. Wen, *Advanced Functional Materials* 2022, 32, 2207466.

[17] L. Zhou, Z. Zeng, J. Liu, F. Zhang, X. Bian, Z. Luo, H. Du, P. Zhang, Y. Wen, *Advanced Functional Materials* 2024, 34, 2312499.
